# The chemosensory receptors of codling moth *Cydia pomonella–*expression in larvae and adults

**DOI:** 10.1038/srep23518

**Published:** 2016-03-23

**Authors:** William B. Walker, Francisco Gonzalez, Stephen F. Garczynski, Peter Witzgall

**Affiliations:** 1 Department of Evolutionary Neuroethology, Max Planck Institute for Chemical Ecology, 07745 Jena, Germany; 2 Chemical Ecology Unit, Department of Plant Protection Biology, Swedish University of Agricultural Sciences, 23053 Alnarp, Sweden; 3 Yakima Agricultural Research Laboratory, United States Department of Agriculture-Agricultural Research Service, Wapato, WA 98951, USA

## Abstract

Olfaction and gustation play critical roles in the life history of insects, mediating vital behaviors such as food, mate and host seeking. Chemosensory receptor proteins, including odorant receptors (ORs), gustatory receptors (GRs) and ionotropic receptors (IRs) function to interface the insect with its chemical environment. Codling moth, *Cydia pomonella*, is a worldwide pest of apple, pear and walnut, and behavior-modifying semiochemicals are used for environmentally safe control. We produced an Illumina-based transcriptome from antennae of males and females as well as neonate head tissue, affording a qualitative and quantitative analysis of the codling moth chemosensory receptor repertoire. We identified 58 ORs, 20 GRs and 21 IRs, and provide a revised nomenclature that is consistent with homologous sequences in related species. Importantly, we have identified several OR transcripts displaying sex-biased expression in adults, as well as larval-enriched transcripts. Our analyses have expanded annotations of the chemosensory receptor gene families, and provide first-time transcript abundance estimates for codling moth. The results presented here provide a strong foundation for future work on codling moth behavioral physiology and ecology at the molecular level, and may lead to the development of more precise biorational control strategies.

The codling moth, *Cydia pomonella* L. (Lepidoptera, Tortricidae), is an economically significant and worldwide pest of apple, pear and walnut. Air permeation with synthetic sex pheromone is used to disrupt pre-mating olfactory communication for areawide codling moth control. This demonstrates the potential of behavioral manipulation with semiochemicals, but current methods are not yet sufficient to contain high population densities^1^. Codling moth is also a model species for investigations of the role of plant odorants in sexual communication and host finding in insect herbivores^2^,^3^,^4^. A greater understanding of olfactory and other facets of chemosensory communication, leading to the discovery of key signals mediating reproductive and host-seeking behaviours, holds promise for implementation of sustainable insect control and for the study of plant-herbivore interactions. Additionally, consideration of chemosensory-based control of larvae has merit, as it is the larvae that damage the host fruit; however, the molecular determinants that govern larval chemosensory behaviors remain to be characterized.

The chemosensory system, through olfactory pathways, is involved in food, mate and host seeking behaviors, as well as predator and parasitoid avoidance; feeding and egg-laying, on the other hand, are influenced primarily through gustatory pathways^5^. Odorant perception largely occurs in the primary olfactory appendages, the antennae and maxillary palps, which are covered with porous hairs (sensilla). Olfactory sensory neurons (OSNs) project their dendrites into the olfactory sensilla, and odorant receptor (OR) proteins are located in the dendritic cell membrane of OSNs and serve as the molecular interface between the insect and its odor environment^6^. Similarly, the sense of taste is mediated by gustatory receptors (GRs) in the dendritic membranes of gustatory receptor neurons (GRNs), which project into sensilla on appendages with gustatory function, including proboscis, tarsi and ovipositor^7^. In both cases, binding of chemical stimuli from the environment activates receptor proteins, and ultimately leads to receptor-mediated neuron depolarization and transmission of the chemical sensory message to the brain^6^.

Insect ORs were first discovered through data-mining of the *Drosophila melanogaste*r genome^8^,^9^,^10^, and subsequently it was determined that ORs display a high degree of divergence, both within and across species^11^. A large family of insect GRs were discovered in *Drosophila* soon after the annotation of the ORs^12^, and it was demonstrated that both ORs and GRs comprise an evolutionarily related superfamily of insect chemoreceptor proteins (CRs)^13^. Finally, a second family of olfactory receptors, known as ionotropic receptors (IRs), was identified, also initially in *D. melanogaster*^14^.

In Lepidoptera, the study of CR families became possible when the genome of the silk moth, *Bombyx mori* was sequenced^15^,^16^. The advent of transcriptomic analyses further facilitated large-scale discovery and characterization of chemosensory gene transcripts^17^. A PCR-based technique was successfully used to identify putative pheromone receptor (PR) fragments from various Lepidoptera, including five from codling moth^18^. Subsequently, a transcriptomics-based approach identified a larger family of antennal-expressed ORs, GRs, and IRs in codling moth antennae^19^, including ORs belonging to the lepidopteran OR clade of putative PRs^20^. Recent reports have identified larger sets of ORs in three tortricid moths^21^,^22^ and a full set of 68 GRs have been identified in *B. mori*, including broadly conserved carbon dioxide (CO_2_) and sugar receptors that have not yet been found in *C. pomonella*^16^. Moreover, many previously reported codling moth OR sequences did not contain complete open reading frames (ORFs)^19^.

In this current study, we used Illumina-based RNA-sequencing (RNA-Seq) methodology to provide deeper transcriptome coverage of *C. pomonella* antennae and larval heads. Our goal was to provide a more comprehensive identification of adult expressed CRs, identify ORs expressed in larvae, and provide initial abundance estimates of CR gene expression. Here we describe 21 novel ORs, 19 GRs and 6 IRs, and additionally provide complete ORF sequence information for 20 previously identified ORs. Furthermore, we report several ORs displaying sexually biased expression in adult moths and identify larval CR transcripts for the first time in this species.

## Results

### Transcriptome assembly and overview

A *de novo C. pomonella* transcriptome was generated by an Illumina HiSeq 90PE approach. A total of 159,222,221 filtered sequencing read pairs plus 6,471,828 unpaired reads from male antenna, female antenna and neonate larval head RNA libraries were assembled with the Trinity assembler into 152,890 contigs (>201 nt). After removing redundant sequences, our transcriptome contained 108,218 contig clusters (Trinity gene level) and 145,710 total sequences, with a mean length of 675.44 nt, an N50 length of 1019 nt, and 26,135 sequences greater than 1,000 nt (Supplementary Data S1).

To assess the quality of the transcriptome, we ran a blast query against it with the *D. melanogaster* core eukaryotic genes dataset^23^. A total of 454 out of 457 conserved core gene homologues were present (blast e-value less than 7e-06, similarity greater than 47.72%). The nature of the 50 most abundant gene clusters was quantitatively examined (Supplementary Data S2) to provide a tissue-specific qualitative characterization (Supplementary Fig. S3). In the male antenna, 27 of the top 50 most abundant gene clusters were homologous to genes with putative chemosensory function (16 odorant binding proteins (OBP), 10 chemosensory proteins (CSP), and one sensory neuron membrane protein (SNMP), with an expression share of 41.7% of the summed fragments per kilobase of transcript per million reads (FPKM) value. In female antennae, 30 of the top 50 most abundant gene clusters were homologous to putative chemosensory proteins (20 OBP, 10 CSP), with a 27.3% share of the summed FPKM values. In larval heads, no genes with putative chemosensory function were observed in the 50 most abundant clusters; 39 of the top 50 gene clusters were homologous to either ribosomal proteins (n = 20), muscle-related proteins (n = 8) or mitochondrial related proteins (n = 11), for a collective 63.0% share of the summed FPKM values. The observation of enrichment of OBP and CSP expression in the male and female antennal tissues is consistent with a recent previous report utilizing similar methods^24^.

### Odorant Receptors

Through qualitative transcriptome analysis, 58 putative ORs were identified (Supplementary Data S4), of which 47 were determined to contain full-length ORFs, based upon the presence of predicted start and stop codons and 5′ and 3′ untranslated regions (UTR).

Further assembly of our transcriptome, combining incomplete transcripts with published data^19^, yielded 53 full-length OR sequences, including complete ORFs for 20 of 21 previously incomplete OR transcripts^19^, and 21 novel ORs (Supplementary Table S5). Six previously annotated ORs comprise 3 unique gene models, where N and C terminal encoding sequence fragments were reported as unique transcripts (previously OR7/OR41, OR27/OR29 and OR36/39). Additionally, through blast homology analysis, the previously annotated OR43 and OR44 fragments have been determined to represent 3′ UTR sequence and do not encode OR proteins.

**Figure 1:**
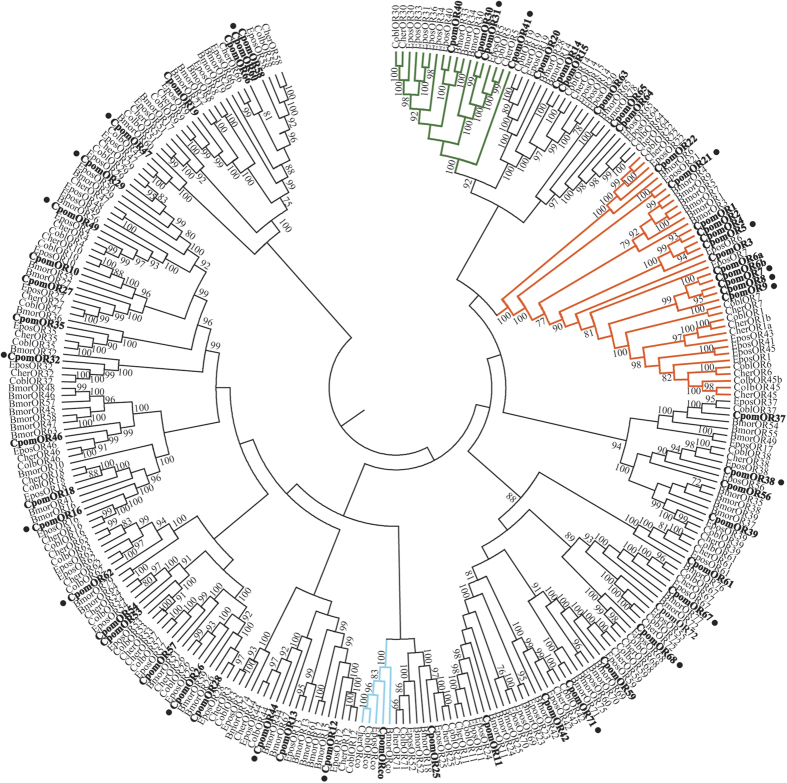
Maximum likelihood phylogenetic tree of candidate CpomOR sequences with other lepidopteran OR sequences. Unrooted. Includes sequences from *Cydia pomonella* (Cpom), *Epiphyias postvittana* (Epos), *Ctenopseustis obliquana* (Cobl), *C. herana* (Cher) and *Bombyx mori* (Bmor). Branches of the Orco clade are colored light blue; branches of the moth “pheromone receptor” clade are colored orange; branches of the secondary clade with sex-biased receptors are colored green; *C. pomonella* ORs are indicated with a larger bold font, and novel *C. pomonella* ORs are marked with a “•”. Node support was assessed with 600 bootstrap replicates and values greater than 70% are shown.

Based on our findings and recent transcriptome reports of OR repertoires in other tortricids^21^,^22^, we present a revised nomenclature of *C. pomonella* ORs (Table 1). Our annotations did not include an additional 13 partial transcript cluster sequences that contained protein-coding sequence fragments homologous to ORs and may represent novel, functional gene transcripts (Supplementary Data S6). The top insect OR blast hits for the translated protein sequences for each of these fragments had e-value scores less than or equal to 7e-08. The *C. pomonella* ORs are presented phylogenetically within the context of other tortricid moth species from which large OR repertoires have been published, along with *B. mori* ORs serving as a lepidopteran outgroup (Fig. 1)^15^,^21^,^22^.

For most ORs, only one unique ORF was identified. However, two protein encoding transcripts for OR6 were identical, except for their C-terminal ends (variant sequence after amino acid residue 398T). These putative variant transcripts have been confirmed through molecular cloning and sequencing, and are annotated as OR6a/OR6b. To provide a greater degree of confidence in the transcriptomic sequences, we cloned and sequenced complete ORFs of several other ORs, namely, CpomOR2a/b/c (see below), CpomOR5, CpomOR30, CpomOR39, CpomOR41 and CpomOR71. For each of these receptors, all full length ORF nucleotide sequences identified in our primary transcriptome displayed between 98.52 and 99.52% identity to a sequenced clone (Supplementary Data S7). Comparison of the 16 ORs that contained complete ORFs in both this transcriptome and the previously reported assembly^19^ revealed that nucleotide sequences were at least 98.2% identical within the protein coding region.

The OR2 locus was present as a complex cluster of sequences. Four variant transcripts encoded similar N-terminal fragments. Three additional transcripts encoded C terminal fragments; these transcripts contained identical C-terminal ORFs that overlapped with the N-terminal fragments, but were variant in their 3′ UTRs. The C-terminal ends of these fragments were nearly identical in amino acid sequence to three receptors (named CpOR1a, CpOR11 and CpOR11a) previously identified by 3′ RACE^18^. We obtained full-length ORFs by 5′ RACE using primers designed in the 3′ UTR, each of which contained unique nucleic acid sequence. These receptors have been annotated as CpomOR2a, CpomOR2b and CpomOR2c. Analysis of the full-length amino acid sequences encoded by the transcripts for CpomOR2a/2b/2c indicate three unique, but highly similar transcripts (Supplementary Fig. S8). Comparison of the nucleic acid sequences show that CpomOR2a is 92% identical to OR2b and 89% identical to OR2c, while OR2b is 89% identical to OR2c (Supplementary Fig. S9). The deduced proteins for the CpomOR2 group were also highly similar, with OR2a being 88% identical and 91% similar to OR2b, OR2a is 84% identical and 89% similar to OR2c, and OR2b is 84% identical and 90% similar to OR2c. Analysis of seven individual clones obtained from CpomOR2a revealed that there might be two forms of this receptor. Comparison of the OR2a clones revealed 18 distinct single nucleotide polymorphisms (SNPs) resulting in 6 amino acid changes (Supplementary Figs S10 and S11), with OR2a1 and OR2a2 being 98% identical on the nucleic acid level and sharing 96% identity and 98% similarity on the amino acid level.

**Figure 2:**
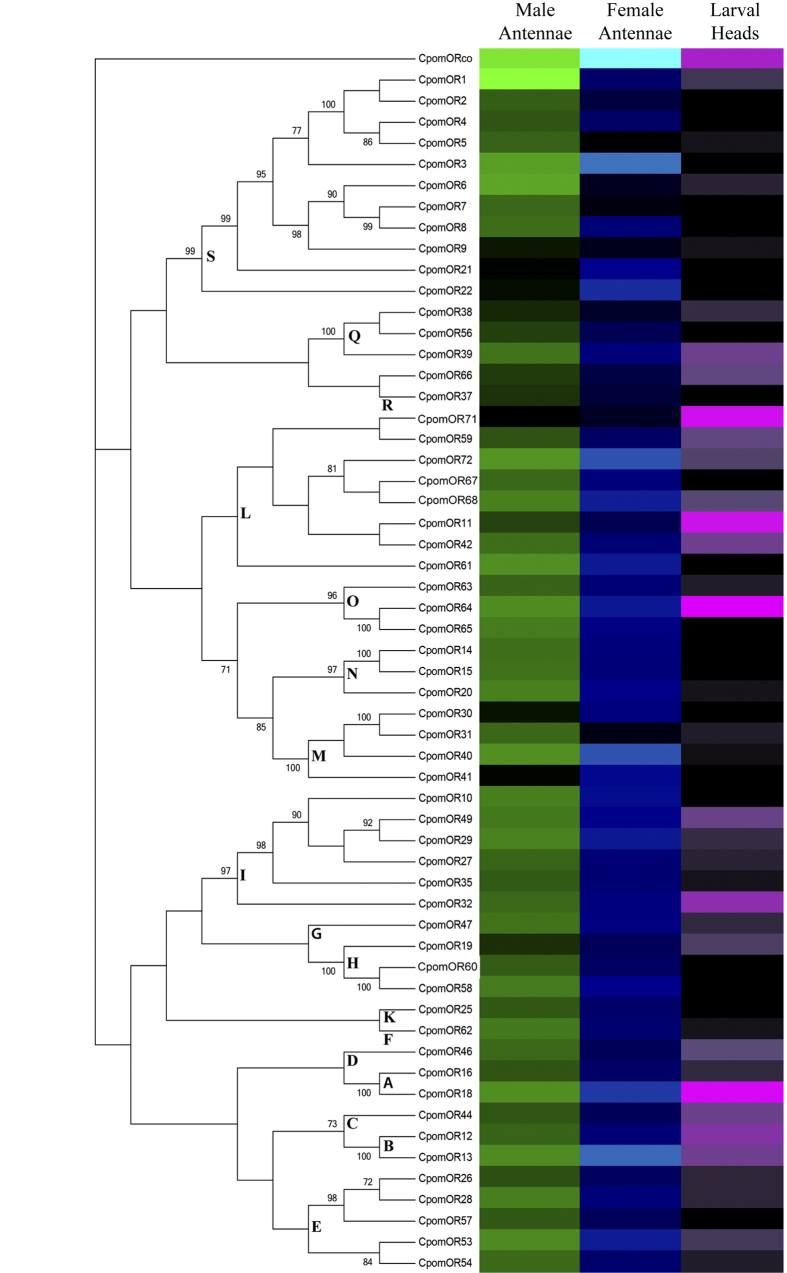
Heat-plot of relative expression values for CpomORs. Estimation of abundance values determined by read mapping. Black indicates low/no expression, dark colors indicate low/moderate expression, bright colors indicate moderate/high expression. Color plots represent binary log of FPKM plus one for each gene (See Supplementary Data S12 for raw data). Color scales for each tissue type are independent of other tissue types. Range of values for Male Antennae: 0.04–10.14; Female Antennae: 0.29–9.62; Larval Heads: 0.00–2.54. Letters are indicative of lepidopteran OR subfamily clade nomenclature as inferred from de Fouchier *et al*., (unpublished data, manuscript submitted).

For quantitative analysis of OR expression levels, we compared FPKM values for transcript clusters. For male antennae, estimated OR transcript expression levels ranged from 0.03 to 1129.88 FPKM (Fig. 2, Supplementary Data S12). OR1 (1129.88 FPKM), OR6 (83.68 FPKM) and OR3 (67.28 FPKM) were the most abundantly expressed tuning ORs in the male antennae, and all three of these receptors cluster phylogenetically within the lepidopteran pheromone receptor subfamily clade^20^. For female antennae, FPKM values of ORs ranged from 0.22 to 785.91. OR3 (105.54 FPKM), OR13 (96.59 FPKM) and OR40 (78.65 FPKM) were the most abundantly expressed tuning ORs. In the larval head, FPKM values for ORs ranged from 0 to 4.8. OR64 (4.67 FPKM), OR18 (4.39 FPKM) and OR71 (3.9 FPKM) were the most abundantly expressed in neonate heads. In all three tissue samples, the OR co-receptor, Orco^25^ displayed higher expression levels compared to most tuning ORs (male antennae, FPKM = 987.83; female antennae, FPKM = 785.91; larval heads, FPKM = 4.8).

**Figure 3:**
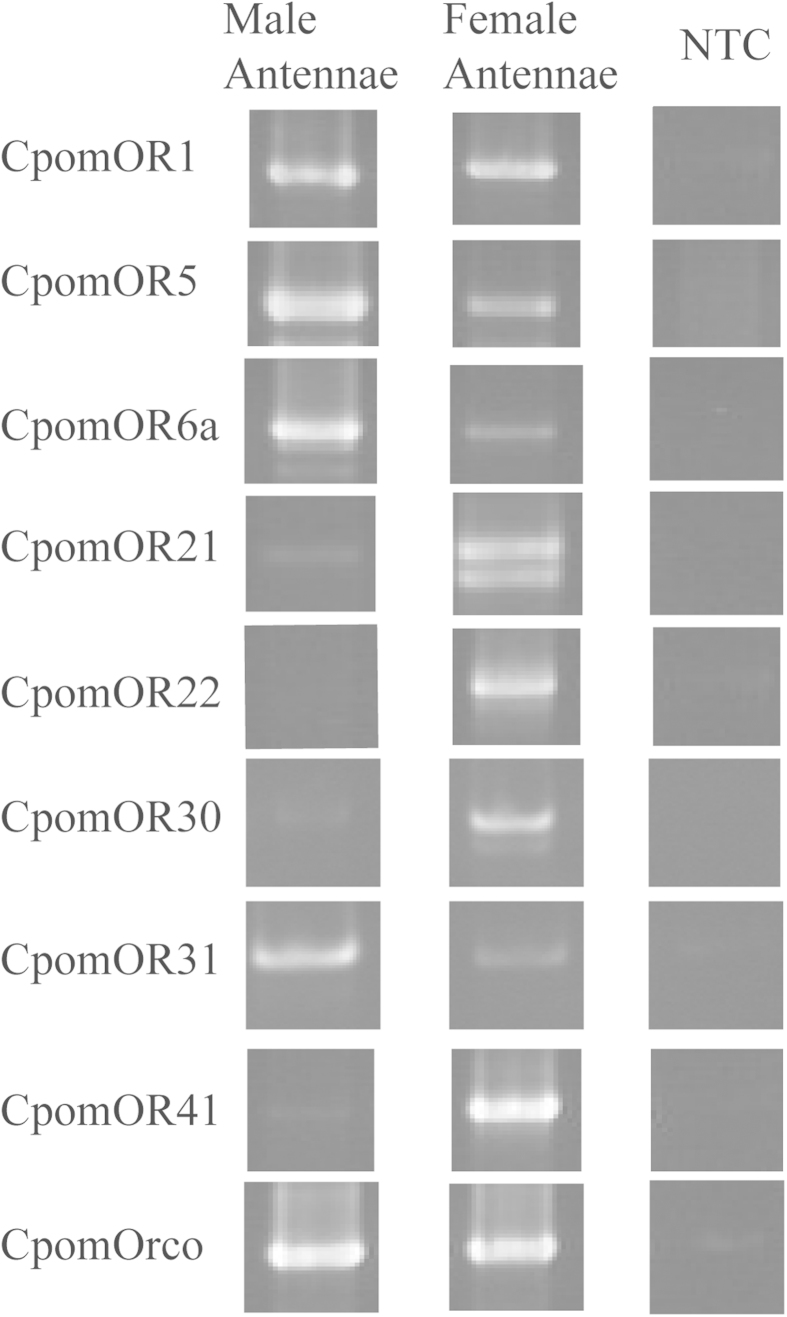
Sex-biased expression of CpomOR genes in *C. pomonella* antennae. Gel electrophoresis of end-point PCR products using antennal cDNA from adult male and female *C. pomonella*. Primers designed to amplify the complete ORF of putative CpomOR genes. Expected sizes are indicated in Supplementary Data S7; Uncropped gel images are shown in Supplementary Fig. S13. NTC: No Template Control.

Comparison of transcript abundance levels between male and female antennae revealed several receptors with sex-enriched or biased expression patterns (FPKM > 10-fold difference between sexes; FPKM < 1 in non-enriched sex). OR1, OR5, OR6, OR7, and OR31 were male-enriched, while OR21, OR22, OR30 and OR41 showed female-enriched expression. Sex-biased enrichment of ORs in male or female antennae was examined qualitatively through end-point PCR analysis (Fig. 3, Supplementary Fig. S13). Consistent with transcriptome FPKM values, male sex-biased amplification was observed for OR5, OR6 and OR31, and female sex-biased amplification for OR21, OR22, OR30 and OR41. We were unable to consistently amplify OR7 in either tissue, and amplification of OR1 was observed in both male and female antennal tissues, which is also consistent with FPKM values.

### Gustatory Receptors

**Figure 4:**
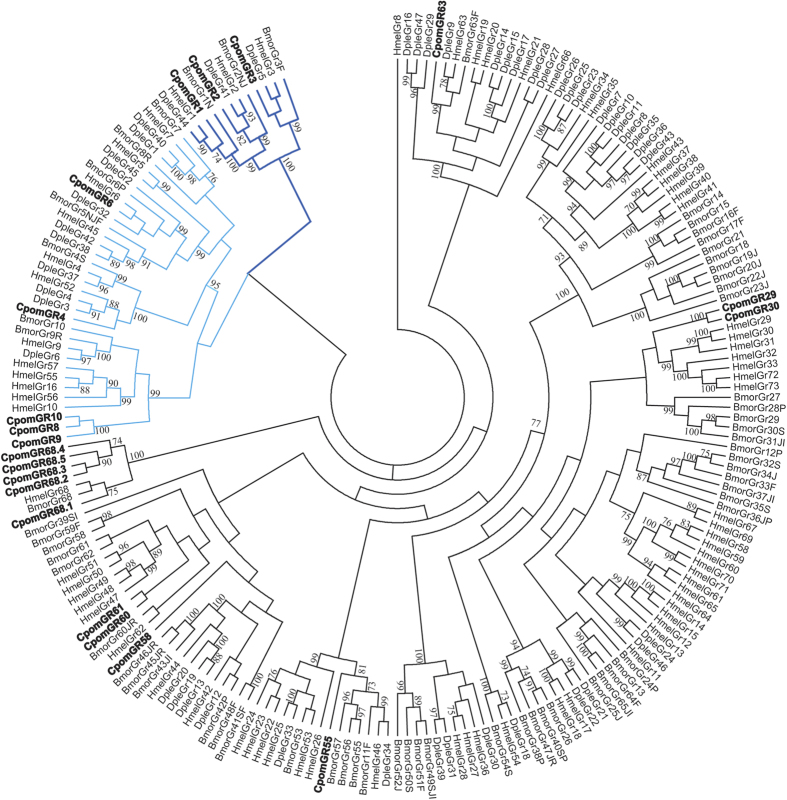
Maximum likelihood phylogenetic tree of candidate CpomGR sequences with other lepidopteran GR sequences. Unrooted. Includes sequences from *Cydia pomonella* (Cpom), *Heliconius melpomene* (Hmel), *Danaus plexippus* (Dple) and *Bombyx mori* (Bmor). Branches containing putative carbon dioxide receptors are colored dark blue; branches containing putative sugar receptors are colored light blue; branches containing putative bitter receptors are colored black; *C. pomonella* GRs are indicated with a larger bold font, and all *C. pomonella* GRs are novel, except CpomGR8. Node support was assessed with 600 bootstrap replicates and values greater than 70% are shown.

The primary transcriptome contained 20 GRs (Supplementary Data S14), including 19 novel gene models (Supplementary Table S5). Seven GR transcripts contained complete ORFs based upon the predicted presence of start and stop codons and 5′ and 3′ UTRs. The conserved C-terminal motif, “TYhhhhhQF” (h = hydrophobic amino acid R group), characteristic of GRs, was identified in 13 transcripts (Supplementary Table S5). CpomGR1 was identified as an incomplete fragment, but the full-length ORF was obtained by RACE to verify sequence. As the most complete moth GR repertoire was first reported for *B. mori*^16^, we adapted *C. pomonella* GR nomenclature to the nearest neighbor *B. mori* homologues where strong bootstrap support was available, including a revision of the previously annotated CpomGR4 to CpomGR8. Additionally, based upon blast homology, 13 transcript clusters contained putative GR fragments deemed too short to annotate with confidence (Supplementary Data S15). A phylogeny of *C. pomonella* GRs are shown together with the complete GR repertoires of *B. mori, Danaus plexippus* and *Heliconius melpomene* (Fig. 4)^16^,^26^,^27^. All candidate codling moth GRs cluster with CO_2_, sugar, and putative bitter receptor families.

**Figure 5:**
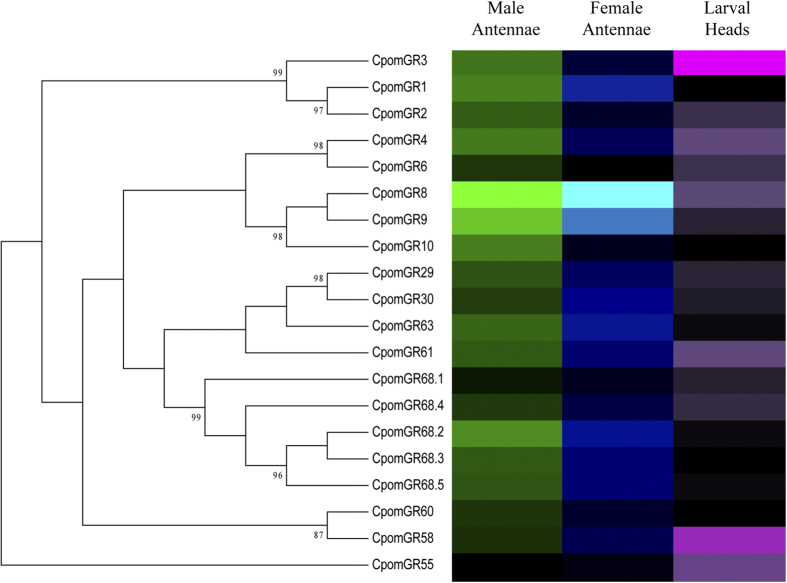
Heat-plot of relative expression values for CpomGRs. Estimation of abundance values determined by read mapping. Black indicates low/no expression, dark colors indicate low/moderate expression, bright colors indicate moderate/high expression. Color plots represent binary log of FPKM plus one for each gene (See Supplementary Data S16 for raw data). Color scales for each tissue type are independent of other tissue types. Range of values for Male Antennae: 0.08–4.99; Female Antennae: 0.21–4.95; Larval Heads: 0.00–1.90

For a quantitative analysis of GR expression levels, we compared FPKM transcript values. In general, CpomGRs were expressed at relatively lower levels than ORs (Fig. 5, Supplementary Data S16). In male antennae, GR transcript abundance levels ranged from 0.06 to 30.86 FPKM, in female antennae, GR FPKM values ranged from 0.16 to 29.91. In both sexes, putative fructose receptor, GR8 (30.86 and 29.91 FPKM, respectively) and a close homologue, GR9 (12.56 and 10.7 FPKM, respectively) were most abundant. In the larval head, GR FPKM values ranged from 0 to 2.73. The most abundantly expressed larval GR was GR2 (2.73 FPKM), which clusters within the carbon dioxide sensing GR clade^16^.

### Ionotropic receptors

Six novel IR encoding transcripts were found (Supplementary Table S5), in addition to the 15 previously reported *C. pomonella* IRs^19^. Complete ORFs, based on predicted start and stop codons, and 5′ and 3′ UTRs, were identified for 15 of the 21 CpomIRs, including 8 of the 12 previously reported incomplete IR gene transcripts (Supplementary Data S17).

**Figure 6:**
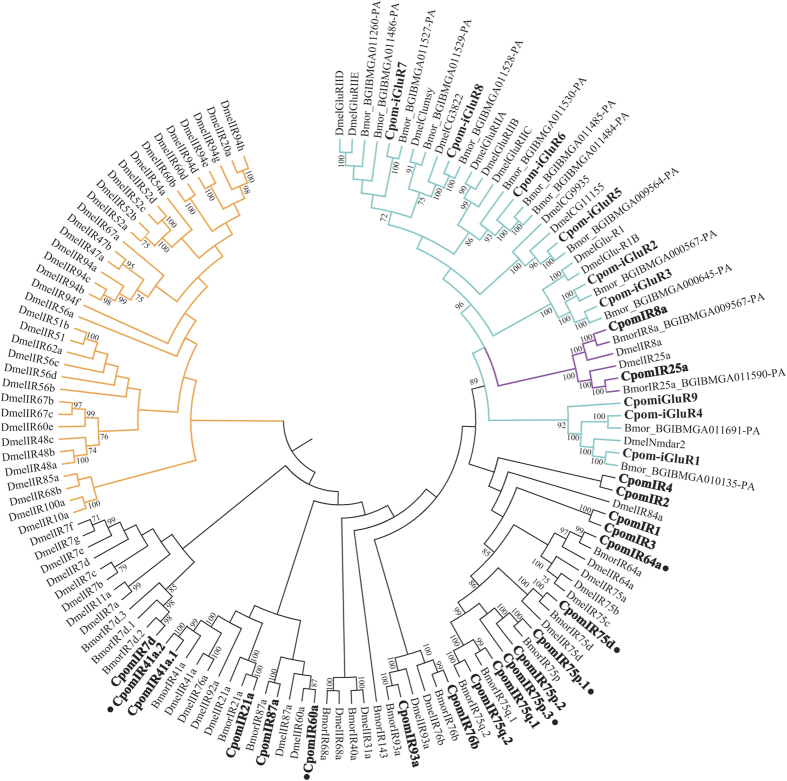
Maximum likelihood phylogenetic tree of candidate CpomIR/iGluR sequences with Bmor/Dmel IR and iGluR sequences. Unrooted. Includes sequences from *Cydia pomonella* (Cpom), *Drosophila melanogaster* (Dmel) and *Bombyx mori* (Bmor). Branches containing putative ionotropic glutamate receptors (iGluRs) are colored light blue; branches containing putative IR co-receptors are colored purple; branches containing divergent IRs are colored orange; branches containing putative antennal IRs are colored black. *C. pomonella* IRs are indicated with a larger bold font, and novel *C. pomonella* ORs are marked with a “•”. Node support was assessed with 600 bootstrap replicates and values greater than 70% are shown.

We compared our predicted IR protein products with IRs and iGluRs identified from *B. mori* and *D. melanogaster* (Fig. 6)^28^, as well as 8 novel CpomiGluRs. Determination of IR status has largely been inferred by phylogenetic analysis of IRs versus ionotropic glutamate receptors (iGluRs), which cluster separately from the IRs. Based on phylogenetic relationships between IRs across these three species, IRs previously annotated as CpomIR41a, CpomIR75, CpomIR75p and CpomIR75q2 have been re-annotated as CpomIR41a.1, CpomIR75q.1, CpomIR75p.2 and CpomIR75q.2, respectively.

**Figure 7:**
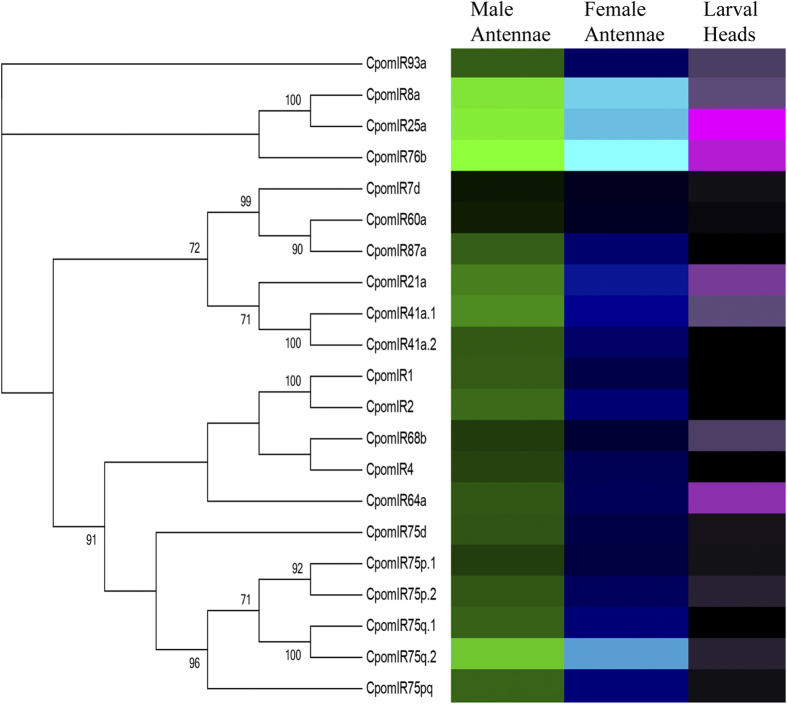
Heat-plot of relative expression values for CpomIRs. Estimation of abundance values determined by read mapping. Black indicates low/no expression, dark colors indicate low/moderate expression, bright colors indicate moderate/high expression. Color plots represent binary log of FPKM plus one for each gene (See Supplementary Data S18 for raw data). Color scales for each tissue type are independent of other tissue types. Range of values for Male Antennae: 0.67–8.59; Female Antennae: 0.99–8.61; Larval Heads: 0.00–3.92.

For an initial estimate of IR abundance levels, FPKM values were compared. For all tissues, three putative IR co-receptors, IR8a, IR25a and IR76b^29^ were the most abundantly expressed. FPKM values in male antennae, female antennae and larval heads, respectively, were 255.83, 278.75 and 1.18 for IR8a; 291.98, 243.76 and 14.17 for IR25a; and 383.43, 390.48 and 7.74 for IR76b. Excluding the putative co-receptors, IR transcript FPKM values ranged from 0.6 to 159.69 in males and 0.99 to 192.58, in females. In male and female antennae, IR75q.2 was the most abundantly expressed candidate tuning IR with FPKM values of 159.69 and 192.58, respectively. In larval heads, excluding the co-receptors, IR FPKM values ranged from 0 to 3.87, with IR64a being the most abundantly expressed IR transcript. (Fig. 7; Supplementary Data S18).

## Discussion

We identified 58 candidate ORs, 20 GRs and 21 IRs expressed in codling moth antennae and neonate heads. In addition, we annotated 21 novel ORs and refined the sequence information of most of the previously identified codling moth CRs. We also provided complete ORFs for 20 of the 21 previously incomplete OR gene products. All of the previously reported codling moth CRs were present in our transcriptome with the exception of one OR (previously annotated OR13), which was reported as a short, incomplete fragment^19^. To account for updated OR information, we present a revised nomenclature for codling moth ORs (Table 1).

Multiple transcripts encoding codling moth OR2 and OR6 were found. For OR6, two transcript variants were identical except at the 3′ end, possibly representing alternatively spliced gene products^30^. Little is known about structure/function relationships between insect ORs and their cognate odorant ligands, however, a recent report demonstrated that the C-terminus of an OR can be essential for enantiomeric selectivity of related compounds^31^. Functional studies are required to determine if this concept holds true for the CpomOR6 variants. The CpomOR2 complex, previously annotated as CpOR1a, CpOR11, CpOR11a and CpomOR5^18^,^19^, was assembled as a cluster of sequences without clear resolution of biologically relevant gene models. Cloning attempts targeting OR2 sequence yielded several different highly related transcripts with complete ORFs. Since the codling moth genome is not yet sequenced, it is unclear if the CpomOR2 transcripts are products of several unique, but highly related genes, or if they result from alternative splicing of a single gene.

A total of 11 ORs were identified that cluster within the lepidopteran pheromone receptor subfamily clade; all of these receptors contained the conserved PWE motif typically found in receptors within this clade^20^. Six of these eleven receptors display sex-biased/enriched expression. It has recently been demonstrated that RNA-Seq read mapping data showing sex-biased expression of moth ORs are reliable when compared to quantitative real-time PCR expression assays for the same transcripts in the same tissue types^22^. Here, we support these findings, demonstrating a general consistency of results for sex-biased receptors using both read-mapping and standard end-point PCR assays.

In male antennae, CpomOR1 was, by far, the most highly expressed tuning OR, and we predict that this receptor may be tuned to the main sex pheromone component, (*E,E*)-8,10-dodecadienol (codlemone). This is in line with the finding that the most abundant OSN type in male *C. pomonella* antennae is responsive to codlemone^32^.

As previously reported, CpomOR22 (formerly OR15) displayed female-specific expression^19^. Other tortricid OR22 orthologues from *C. obliquana* (CoblOR22), *C. herana* (CherOR22) and *E. postvittana* (EposOR22) display female-biased expression as well^21^,^22^. Interestingly, a *B. mori* orthologue to CpomOR22, BmorOR6, has been reported as a male biased OR^15^. While these ORs cluster, phylogenetically, with the PR subfamily clade, no ligands have been reported for any of them.

Strikingly, CpomOR3, though clustering within the PR clade, has been observed to respond to the host plant volatile, pear ester^4^. CpomOR3 is the most and third most highly expressed tuning OR in female and male antennae, respectively. This is consistent with the finding that CpomOR3 is tuned to pear ester, which is a strong bisexual host plant attractant^2^; the common evolutionary lineage of CpomOR3 with putative PRs is impressively corroborated by coding of pear ester in the macroglomerular complex of the male antennal lobe, which responds to sex pheromones^3^.

Beyond the PR clade, a second subfamily clade contained CpomORs displaying sex-biased expression patterns; CpomOR30 and CpomOR41 display female-biased expression, while CpomOR31 displays male-biased expression. This pattern is consistent with sex-biased expression of ORs within this clade from other species: BmorOR30, displays female-biased expression^15^, while the tortricid EposOR30/OR34, CoblOR30 and CherOR30 display male-biased expression^21^,^22^. Although attempts to characterize the odorant response profiles of some of these receptors have not been successful^21^,^33^, the sex-biased expression suggests possible involvement in sexual communication.

We provide a significant update of the GRs expressed in *C. pomonella* antennae, with the annotation of 20 GRs. Based upon phylogeny, this includes conserved putative CO_2_ receptors (CpomGR1/GR2/GR3), sugar receptors (CpomGR4/GR5), including the fructose receptor subfamily clade (CpomGR8/GR9/GR10), and more divergent putative bitter compound receptors (CpomGR29/GR30/GR55/GR58/GR60/GR61/GR63/GR68.1-GR68.5)^16^. CpomGR8 and CpomGR9 are the two most abundantly expressed GRs in both the male and female antennae of *C. pomonella*, and are members of the GR subfamily clade that contains receptors shown to be responsive to D-fructose^34^. The presence of abundantly expressed putative fructose receptors in codling moth antennae is consistent with moth antennal sensitivity to D-fructose^35^.

*Cydia pomonella* GRs expressed in adult antennal and larval head tissues likely represent only a fraction of the complete GR repertoire. In the silkmoth, *B. mori*, 68 GRs have been identified in the sequenced genome^16^, while the genomes of the butterflies *D. plexippus* and *H. melpomene*, encode 57 and 73 GRs, respectively^26^,^27^. As gustatory sensory neurons are primarily found in chemosensory sensilla on leg tarsi and ovipositors, it is predicted that examination of transcript expression in these tissues will lead to the identification of a greater number of GR transcripts.

Compared to ORs, taxa-specific expansions of a GR lineage appear to be more common^27^. Our observations are consistent with this. Five GR68 paralogues were identified in *C. pomonella*, whereas only one gene from this cluster was identified in the genomes of *B. mori, D. plexipus* and *H. melpomene*^27^. Diversification of GR gene repertoire is thought to be essential for host-plant compatibility, especially as it relates to selection of suitable plants for oviposition and larval feeding. Consistent with this, GR68 clusters within the broad family of putative bitter compound GRs that speculatively play a significant role in the detection of host-plant substrates^16^. Recent work on *D. melanogaster* GRs, describes the molecular and cellular mechanisms of bitter taste reception^36^. However, putative bitter GRs have not been functionally characterized in moths.

We identified 21 antennal IR gene transcripts, which is similar to observations in *Manduca sexta*^37^. As with the relatively high antennal expression of the OR co-receptor, Orco, the most highly expressed IR transcripts in both the male and female antennae were the putative IR co-receptors, IR8a, IR25s and IR76b. While antennal-expressed IRs from *D. melanogaster* have been functionally characterized and shown to respond to odorant stimulation^28^,^29^, moth IRs have not been investigated. However, since IRs display phylogenetic homology across insect orders, it is predicted that *C. pomonella* IRs mediate olfactory reception of acids and amines in the antennae^29^.

For all three CR families, subsets of gene transcripts were found to be expressed in the larval head, which was used as proxy for expression in larval olfactory tissues in the antennae and maxillary palps. In adult male and female antennae, all 58 CpomORs had FPKM values greater than zero, while for our larval head sample only 38 ORs displayed non-zero FPKM values. Similarly, in the larval head, positive FPKM values were detected for 16 of 20 GRs and 15 of 21 IRs. In order to provide more thorough verification of CR expression in neonate larvae, we screened a secondary *de novo* transcriptome generated from only the larval head sequenced reads for the presence of CR transcripts; identification of assembled CR sequences is presumed to be indicative of biologically meaningful expression levels. In this larval head transcriptome, sequences of 16 ORs, 8 GRs, and 9 IRs were identified (Supplementary Data S12, S16 and S18).

In our primary transcriptome, the pear ester receptor, CpomOR3, was not detected in the neonate larval head (FPKM = 0). However, FPKM values near the threshold of detection are not reliable, and a fragment identical to this OR in the secondary larval head transcriptome suggests its expression. This finding has been confirmed by independent end-point PCR analysis of expression in *C. pomonella* neonate larvae (Supplementary Fig. S19), and is consistent with a report showing that *C. pomonella* larvae are responsive to pear ester^38^.

CpomOR71 was one of the most highly expressed ORs in the neonate larval head. Larval enriched ORs may be responsive to host plant cues that signal qualitative suitability or unsuitability of the larval host for initiation and continuation of feeding^39^. Interestingly, CpomOR71 is a putative homologue to the *S. litura* receptor, SlituOR12, (49% identity/67% similarity), which is responsive to (*Z*)3-hexenyl acetate^40^. This compound is an abundant compound in apple volatile headspace and may be an important cue for larval host-fruit seeking behavior of codling moth^41^.

We provide here a more complete account of the *C. pomonella* CR repertoire. This is a solid foundation for future research on chemical signals mediating codling moth behavior, as well as a better understanding of its chemosensory system. This know-how will further augment the efficacy of control based on behavior-modifying odorants, and thus help to reduce the use of environmentally harmful chemicals in fruit orchards. As an example, we identified pear ester as the ligand for CpomOR3, which is a pheromone receptor subfamily clade OR^4^. Pear ester is indeed a powerful, bisexual adult and larval attractant^2^,^38^. It will be exciting and rewarding to study other highly expressed CRs, especially those showing distinct sexual bias. Ligands for these receptors are good candidates for further development of sustainable codling moth control.

The highly selective and sensitive response of CpomOR3 to the plant compound pear ester compares to the response of PRs to sex pheromone ligands. In combination with behavioral and physiological studies^3^, it highlights the dual role of plant signals in habitat selection and in premating sexual communication, as well as their interaction with social signals during the phylogenetic divergence of insect herbivores. Moreover, CpomOR3 is rather exclusively tuned to pear ester, which is behaviorally active on its own, and which is specific for apple and pear, the main host plants of codling moth. This challenges the view that moths employ blends of ubiquitous plant volatiles, in host specific ratios, for host finding^42^,^43^. A dedicated receptor enables codling moth to pick up even small amounts of the host-specific pear ester signal, at a resolution that compares to perception of pheromone^44^. This finding illustrates that the codling moth chemosensory transcriptome is a rich substrate and provides inspiration for studying the chemical ecology of host finding in codling moth and other insect herbivores.

## Methods

### RNA sequencing

Samples of first instar larval heads, adult male antennae, and adult female antennae were prepared for RNA sequencing (see Insect Rearing and RNA Extraction; Supplementary Materials and Methods) at the National Genomics Infrastructure sequencing facility (Uppsala, Sweden). RNA libraries for sequencing were prepared using TruSeq Stranded mRNA Sample prep kit with 96 dual indexes (Illumina, CA, USA) according to the manufacturer’s instructions, with the following changes: the protocols were automated using an Agilent NGS workstation (Agilent, CA, USA) using purification steps as described^45^,^46^. Samples were clustered using cBot and sequenced on a HiSeq2500 (HiSeq Control Software 2.2.38/RTA 1.18.61) with a 2 × 126 setup in RapidHighOutput mode. Bcl to Fastq conversion was performed using bcl2Fastq v1.8.3 from the CASAVA software suite. The quality scale is Sanger/phred33/Illumina 1.8+.

All sequence read files were delivered to our project account on the UPPMAX Computational Science server (Uppsala, Sweden). For each sample, two fq files were produced, one containing all left-pair reads (sampleX_1.fq) and one containing all right-pair reads (sampleX_2.fq).

### Bioinformatic pipelines - pre-assembly, assembly, post-assembly

Initial quality control measures were undertaken prior to transcriptome assembly. For this, Trimmomatic software (version 0.32) was utilized to remove reads in which the sequencing adapter information was present, and also trim low quality bases from the 3′ end of each read. For this, starting from the 3′ terminal nucleotide and moving in the 5′ direction, each base having a PHRED score lower than 20 was removed until a base is encountered with a PHRED score greater than or equal to 20^47^. For execution of the Trimmomatic software, the ILLUMINACLIP (sequencing adapters file: TruSeq3-PE.fa:2:30:10) and TRAILING:20 commands were used. The output of this was two fq files for each input fq file, as described above, with trimmed paired and unpaired reads (sampleX_1_paired.fq and sampleX_1_unpaired.fq).

Trimmomatic-processed reads from all of the sample fq files were assembled, *de novo*, into one transcriptome with Trinity software (release version r20140717)^48^. The Trinity perl script was executed with the following parameters specified: –seqType fq –JM 30G –CPU 16 –bflyCPU 3. The output transcriptome file from this process was the Trinity.fasta file. To facilitate deeper analysis of transcript expression in the neonate larvae, a secondary transcriptome was generated from the Trimmomatic-processed larval head sequence files only using the same procedures described above.

In order to facilitate unambiguous read mapping of individual sample reads back to unique locations on assembled transcriptome sequences for downstream quantitative analyses, the software cd-hit-est (version 4.5.4-2011-03-07) was used to identify and remove redundant sequences that share 98% or greater identity with other sequences^49^. The Trinity.fasta file was used as input, program parameters -c 0.98 -n 10 were specified and the output file was named Trinity98.fasta. In cases where sequences shared greater than 98% identity, but were of different sizes, the largest of the sequences were retained in the fasta file.

To assess completeness of the transcriptome, a *D. melanogaster* CEGMA transcript file, consisting of transcripts of 457 genes that are highly conserved across all eukaryotes^23^, was blasted against the Trinity98.fasta transcriptome. For this process, a BLAST nucleotide database was generated from the Trinity98.fasta file, and a tblastx query was performed (blast version 2.2.29+) with an e-value threshold of 1e-5 required for reporting of blast hits^50^.

### Chemosensory transcript annotation procedures

Text files were compiled in fasta format with the protein sequences encoded by previously characterized transcripts of *C. pomonella* ORs, GRs, and IRs^19^. Blast nucleotide databases were created from the Trinity98.fasta files and were queried by the protein sequence fasta files for each of the CR gene families. For this procedure, Blast version 2.2.29+ was used to perform a tblastn query and a minimum e-score threshold of 1e-05 was required for reported hits, additional parameters included num_descriptions 50 and -num_threads 16. Blast output files were generated with output format six with the following parameters: qlen, qseqid, slen, sseqid, evalue, bitscore, score, pident, indent, ppos, positive, sframe. For each of the previously annotated chemosensory gene transcripts, the top blast hit transcript cluster was extracted from the Trinity98.fasta file with an in-house command line script. Relevant transcript sequences were translated into protein sequence with the ExPASy web Translate tool^51^, and the protein sequences were aligned to reference annotations with the ClustalOMEGA web tool (http://www.ebi.ac.uk/Tools/msa/clustalo/)^52^.

Novel CR genes were identified and annotated in an iterative search process. First, all sequences that were reported in the original blast searches that did not correlate to previously annotated genes were examined. In order to reduce the possibility of mis-annotating two uncoupled fragments of the same gene as distinct genes, fragment sequences with ORFs shorter than 50% of the average length of a gene in a given gene family (OR = 408 amino acids, GR = 428 amino acids, IR = 674 amino acids) were generally excluded from annotation and further analysis unless they displayed high blast homology against unique homologous genes. The protein sequences of novel gene candidates were incorporated into fasta files for each gene family and an additional tblastn query was performed against the Trinity98.fasta nucleotide databases in order to determine if any further gene candidates would be identified.

### Quantitative analysis of chemosensory gene expression levels with RSEM

Prior to quantitative analyses, Trinity Sequence clusters containing annotated CR transcripts were edited as necessary (see Manual Editing; Supplementary Materials and Methods). Read mapping of individual sample reads to the *de novo* generated transcriptome and subsequent expression level abundance estimations were carried out, as described^53^ with the Trinity perl script “align_and_estimate_abundance.pl in the r20140717 release version of Trinity, using version 1.2.12 of RSEM^54^, version 0.12.6 of Bowtie^55^ and version 0.1.19 of Samtools^56^, with parameters –est method RSEM and –aln method bowtie. The Trinity98.fasta file was used for reference transcripts input (command “prep_reference”) and the trimmomatic processed fastq reads were used as mapping input. The manually edited gene_trans_map file (see Manual Editing; Supplementary Materials and Methods) was used as input for determining FPKM values^57^ of each Trinity gene-level cluster, as a basis for estimation of transcript expression abundance levels. The parameter “coordsort_bam” was also selected in order to generate the appropriate files for visualization of read mapping in the Integrative Genomics Viewer (IGV; see Manual Editing; Supplementary Materials and Methods)^58^.

### cDNA synthesis and PCR assay of OR expression

For these experiments, we used the same RNA samples (at 250 ng/μL) that were sourced to the RNA-Seq analyses mentioned above. For the adult male and female antennal samples, 2.88 μg of total RNA were input towards cDNA synthesis with the RevertAid H Minus First Strand cDNA synthesis kit (Life Technologies, Carlsbad, CA, USA) according to standard manufacturers protocol. For these reactions, cDNA synthesis was primed with oligoDT primers and a final reaction volume of 20 μL was diluted with water to 30 μL after synthesis.

For PCR assays, Dream Taq DNA polymerase (Life Technologies, Carlsbad, CA, USA) was utilized. 1 μL of cDNA template was input to a 25 μL mixture with 2.5 μL 10× Dream Taq Buffer, 1 μL each of 10 μM gene specific forward and reverse primers (Supplementary Data S7), 0.5 μL 10 mM dNTPs and 0.25 μL Dream Taq Polymerase (5 U/μL). In case of the no-template negative control, 1 μL of deionized water was added in place of the cDNA. For all assays, thermocycling was conducted as follows: Initialization–94 °C 2 min; Amplification–31 cycles of 94 °C 30 s, 55 °C 30 s, 72 °C 30 s; Final Extension–72 °C 10 min. 5 μL of each PCR product were examined on a 1.5% agarose gel after 35 minutes of standard electrophoresis at 100 V and 15 min of staining with standard application of Gel Red (Biotium Inc., Hayward, CA, USA). For each gene, technical replicates were performed in triplicate to verify consistency of amplification.

### Phylogenetic analysis of chemosensory gene families

For the qualitative report of CR transcripts, published sets of CRs from different species were used for comparison with our data. In the case of ORs, the available repertoires of the tortricid moths, *E. postvittana*^22^, *Ctenopseustis obliquana* and *C. herana*^21^, and the silkmoth, *B. mori*^15^, as outgroup, were compared with the new set of *C. pomonella* ORs. Regarding GRs, the available sets from the monarch butterfly, *D. plexippus*^26^, the postman butterfly, *H. melpomene*^27^ and *B. mori*^16^ were used for comparison with *C. pomonella* GRs. For IRs, comparisons were made between the new set of codling moth IRs and the reported IR sets from *B. mori* and *D. melanogaster*^28^.

Amino acid sequences of ORs, GRs or IRs were aligned using MAFFT online version 7.220 (http://mafft.cbrc.jp/alignment/server/phylogeny.html) through the FFT-NS-i iterative refinement method, with JTT200 scoring matrix, unalignlevel 0.3, “leave gappy regions” set, and other default parameters^59^. Aligned sequences were used to calculate the evolutionary history of receptors of each gene family with MEGA7 software^60^ in command line, with the following parameters: Maximum Likelihood Tree Method with the JTT-F’ model, uniform rates, use all sites, nearest neighbor interchange heuristic method, very strong branch swap filter and default automatic NJ/BioNJ initial tree. The boostrap consensus of each phylogenetic tree was inferred from 600 replicates. Output consensus Newick format trees were compiled with MEGA5 software^60^ and edited with Adobe Illustrator.

Additional trees containing only codling moth receptors were constructed using the same procedures to facilitate quantitative analysis (See next section below). For the CpomORs, OR subfamily clade nomenclature was adapted from de Fouchier *et al*. (unpublished data, manuscript submitted). For this, an alignment and phylogenetic tree was made for ORs from *C. pomonella, S. littoralis* and *B. mori* (data not shown) with the same parameters indicated in the previous paragraph. With *S. littoralis* and *B. mori* receptors as reference, letters were assigned to CpomORs according to phylogenetic clustering into specific subfamily clades. For all previously defined subfamily clades (de Fouchier *et al*., unpublished data, manuscript submitted), bootstrap support was greater than 52%.

### Heatmap presentation of transcript expression

Heatmap plots were generated for the binary logarithm of raw FPKM plus 1 values. These plots were made using the conditional formatting function in Microsoft Excel, with a three-color scale. For each plot, the minimum value was set to number type, with a value of zero, and displayed as black; midpoint was set to percentile type, with a value of 75, and displayed as dark color; maximum was set to highest value type, and displayed as bright color. For ORs, GRs, and IRs, the range was specified for each tissue type independently, such that the color gradient was set based upon the highest FPKM values within each tissue, not across all tissues. After heatmap plots were generated, number values were wiped from visibility through excel cell formatting of custom category with formatting code“;;;”.

## Additional Information

**Accession Codes**: Transcriptome raw reads sequence data are available through the NCBI Sequence Read Archive (Accession Numbers: SRX1082029, SRX1082030 and SRX1082032)). Chemosensory Receptor sequences identified from the primary *C. pomonella* transcriptome assembly are available through NCBI. Novel sequences are included in a Transcriptome Shotgun Assembly project that has been deposited at DDBJ/EMBL/GenBank under the accession GDKB00000000. The version described in this paper is the first version, GDKB01000000. Previously identified sequences have been updated, where appropriate, within Genbank deposits reported previously [19]. All coding protein sequences are available as part of the supporting data.

**How to cite this article**: Walker, W. B., III *et al*. The chemosensory receptors of codling moth *Cydia pomonella* - expression in larvae and adults. *Sci. Rep.* **6**, 23518; doi: 10.1038/srep23518 (2016).

## Supplementary Material

Supplementary Information

Supplementary Data S1

Supplementary Data S2

Supplementary Data S7

Supplementary Data S12

Supplementary Data S16

Supplementary Data S18

## Figures and Tables

**Table 1:** *Cydia pomonella* Odorant Receptors: Revised Nomenclature and ORF Status in comparison to Bengtsson *et al*.^19^.

Current/Updated Nomenclature	Previous Nomenclature	Previous ORF Completion Status	Updated Status
CpomOrco	CpomOR2	Complete	*
CpomOR1	CpomOR4	Complete	*
CpomOR2	CpomOR5	Incomplete	Incomplete^3^
CpomOR3	CpomOR3	Incomplete	*^a^
CpomOR4	CpomOR6	Incomplete	Incomplete^b^
CpomOR6	CpomOR1	Incomplete	Complete^2^
CpomOR10	CpomOR28	Complete	*
CpomOR11	CpomOR11	Incomplete	
CpomOR13	CpomOR8	Incomplete	Complete
CpomOR14	CpomOR14	Complete	*
CpomOR15	CpomOR20	Complete	*
CpomOR18	CpomOR10	Complete	*
CpomOR19	CpomOR19	Complete	*
CpomOR20	CpomOR18	Complete	*
CpomOR22	CpomOR15	Incomplete	Complete^c^
CpomOR25	CpomOR21	Complete	*
CpomOR27 1	CpomOR27/OR29	Incomplete	Complete
CpomOR28	CpomOR26	Incomplete	Complete
CpomOR30	CpomOR30	Incomplete	Complete^c^
CpomOR35	CpomOR35	Incomplete	Complete
CpomOR37 1	CpomOR36/OR39	Complete	*
CpomOR39	CpomOR38	Complete	*^c^
CpomOR40	CpomOR33	Incomplete	Complete
CpomOR46	CpomOR16	Complete	*
CpomOR53	CpomOR9	Incomplete	Complete
CpomOR54 1	CpomOR7/OR41	Incomplete	Complete
CpomOR56	CpomOR37	Incomplete	Complete
CpomOR57	CpomOR31	Complete	*
CpomOR58	CpomOR34	Complete	*
CpomOR59	CpomOR12	Complete	*
CpomOR61	CpomOR17	Incomplete	Complete
CpomOR63	CpomOR23	Incomplete	Complete
CpomOR64	CpomOR24	Complete	*
CpomOR65	CpomOR22	Incomplete	Complete
CpomOR66	CpomOR32	Incomplete	Incomplete
CpomOR72	CpomOR40	Incomplete	Complete
¶	CpomOR13		
#	CpomOR43		
#	CpomOR44		

^1^Single genes that were previously annotated as two separate ORs.

^2^Two complete splice variants identified and confirmed with cloned sequence.

^3^Unresolved cluster of sequence identified in transcriptome, but 3 isoforms confirmed with cloned sequence.

^*^Sequence not found and thus not re-annotated. This sequence, while annotated, was never deposited in an on-line database, and has been replaced in nomenclature by a different gene entirely.

^#^Sequence was determined to represent non-coding 3′ UTR sequence and thus not re-annotated.

^*^Status not updated as it was previously reported as complete.

^a^Sequence cloned and published in ref. 4.

^b^Sequence incomplete in transcriptome, but complete ORF identified when merged with sequence from ref. 19.

^c^Complete ORF in transcriptome, sequence confirmed with cloned sequence.
