# Supplementary material for: The chemosensory receptors of codling moth Cydia pomonella–expression in larvae and adults
Source: Sci Rep. 2016 Mar 23;6:23518. doi: 10.1038/srep23518 (PMC4804390; doi:10.1038/srep23518)
Supplement: Supplementary Information [file srep23518-s1.pdf]

The chemosensory receptors of codling moth *Cydia pomonella* – expression in larvae and adults

William B Walker III, Francisco Gonzalez, Stephen F. Garczynski, Peter Witzgall

Supplementary Materials Table of Contents

**Title Page**	**pg. 1**
**Supplementary Materials and Methods**	**pg. 2-8**
**Supplementary Figure S3**	**pg. 9**
**Supplementary Data S4**	**pg. 10-20**
**Supplementary Table S5**	**pg. 21-22**
**Supplementary Data S6**	**pg. 23-24**
**Supplementary Figure S8**	**pg. 25**
**Supplementary Figure S9**	**pg. 26**
**Supplementary Figure S10**	**pg. 27-29**
**Supplementary Figure S11**	**pg. 30**
**Supplementary Figure S13**	**pg. 31**
**Supplementary Data S14**	**pg. 32-35**
**Supplementary Data S15**	**pg. 36-37**
**Supplementary Data S17**	**pg. 38-45**
**Supplementary Figure S19**	**pg. 46**
**Supplementary Data S1**	*
**Supplementary Data S2**	*
**Supplementary Data S7**	*
**Supplementary Data S12**	*
**Supplementary Data S16**	*
**Supplementary Data S18**	*

*** submitted as separate excel file.**

Insect rearing

Codling moth last-instar larvae were obtained from Andermatt Biocontrol (Grossdietwil, Switzerland) and maintained under a 16L:8D photoperiod, at 23 +/- 1 °C and 70 +/-5% relative humidity. Eclosing adults had access to water and 10% sugar solution. First-instar (neonate) larvae and 2 day-old male and female moths were used for dissections and RNA extractions.

RNA extraction

Larval heads were dissected from 1000 specimens. For adult male and female moths, antennae were dissected from 160 and 150 individuals, respectively. Larval heads and adult antennae were dissected with forceps and transferred into 1.5-mL microcentrifuge tubes (Eppendorf, Hamburg, Germany), which were chilled in liquid nitrogen. Directly after dissections, 500 µL of Trizol (Life Technologies, Carlsbad, CA, USA) were added to the Eppendorf tube. Individual samples were stored at -80°C until all samples were ready for processing.

Total RNA was extracted and purified with a combined approach of Trizol-based extraction followed by spin column purification. First, frozen samples were thawed on ice and then homogenized in Trizol with a pestle, by hand. Homogenized samples were then snap-frozen by placing the sample tubes in liquid nitrogen, and then allowed to thaw at room temperature. Samples were again homogenized with a pestle by hand. After adding an additional 500 µL of Trizol reagent, each sample was briefly vortexed and incubated at room temperature for 5 min. 200 µL of Chloroform (Riedel de Haen, Seelze, Germany) were then added and samples were vortexed for 20 seconds and incubated at room temperature for 15 minutes. Samples were then centrifuged at max speed at 4°C for 15 min. The upper aqueous phase (ca. 600 µL) was then pipetted off into a clean 1.5-mL microcentrifuge tube. An equal amount of 100% isopropanol (Sigma Aldrich, Saint Louis, MO, USA) was added, as well as 3 µL of 5 mg/mL glycogen

(Life Technologies, Carlsbad, CA, USA). Samples were then mixed by gentle inversion several times and stored at -20°C overnight. To complete the isopropanol precipitation, samples were then centrifuged at max speed at 4°C for 15 min. Supernatant was decanted by pipetting without disturbing the pellet and excess liquid was removed by inverting the sample tube on a paper towel. 1 mL of ice cold 70% ethanol was added and samples were centrifuged at 7500 RCF at 4°C for 10 minutes. Supernatant was decanted as above; tubes were inverted on a paper towel and allowed to air dry for 15 min. 100 µL of RNAse free water (Life Technologies, Carlsbad, CA, USA) was added and samples were immediately purified by spin column filtration with the RNeasy Mini Kit (Qiagen, Venlo, Netherlands).

For this, 350 µL of Buffer RLT and 250 µL of 100% ethanol were added to each sample and mixed thoroughly by pipetting. Samples were transferred to RNeasy spin columns and RNA was fixed to the filter membrane via centrifugation at 10000 RCF for 15 s at room temperature. Subsequent to this, RNA purification was completed according to manufacturer's protocol for RNA Cleanup, including an on-column DNase digestion, performed with the RNase free DNase kit (Qiagen, Venlo, Netherlands). RNA was eluted with 40 µL of supplied RNase Free water, and immediately assayed for quality and concentration with a Nanodrop 1000 spectrophotometer (Thermo Fisher Scientific, Waltham, MA, USA). For each sample, total RNA quantity was normalized to 250 ng/µL and the 260/280 ratio was 2.17 for male antennae, 2.17 for female antennae and 2.19 for larval heads.

Cloning confirmation of CR sequence and expression

Chemosensory receptor fragments previously identified amongst codling moth expressed sequence tags [1] that were not present in our primary transcriptome were amplified by 3' RACE, 5' RACE or both to confirm their expression. Insects were reared and obtained from a colony maintained at the Yakima Agricultural Research Laboratory (Wapato, WA). Total

RNA extraction, cDNA syntheses and RACE procedures were done as previously described [2]. OR specific primers can be found in Supplementary Data S7.

Several CR ORFs were cloned and sequenced in order to verify the authenticity of sequences identified in the primary transcriptome assembly. For this, OR specific primers were used to PCR amplify the complete ORF from cDNA generated as described in the previous section. For PCR assays, the Advantage 2 proofreading Taq polymerase system was used (Clontech, Mountain View, CA, USA). 1 µL of cDNA template was input to a final volume of 25 µL with 2.5 µL 10X Advantage 2 Taq Buffer, 1µL each of 10 µM gene specific forward and reverse primers (Supplementary Data S7), 0.5 µL 10 mM dNTPs and 0.25 µL Advantage 2 Taq Polymerase mix. For all assays, thermocycling was conducted as follows: Initialization - 94°C 2 min; Amplification – 31 cycles of 94°C 30 s, 55°C 30 s, 68°C 30 s; Final Extension – 68°C 10 min. The entire contents of the PCR reaction was run on a 1.5% agarose gel, and PCR bands of expected size were cut out and purified with the GeneJET gel extraction kit (Thermo Fisher Scientific, Waltham, MA, USA) according to manufacturers protocol. Gel purified DNA was then cloned into the Topo plasmid vector with the pCR8/GW/Topo kit (Life Technologies, Carlsbad, CA, USA) according to manufacturers protocol. Cloned plasmid was transformed into Mach1-T1^R cells with standard heat shock procedure, cultured with 250 µL SOC media and grown overnight on LB agar plates with spectinomycin selection. For each construct, 4-8 colonies were screened with colony PCR for presence of CR sequence, and one colony was selected and cultured in LB media with spectinomycin selection; plasmid DNA was purified with the ZR Plasmid Miniprep Classic kit (Zymo Research, Irvine, CA, USA), according to manufacturers protocol. CR inserts were sequenced from the plasmid preparations with the pCR8/GW/Topo GW1 and GW2 primers at the Eurofins Genomics DNA Sequencing facility (Ebersberg, Germany).

For verification of CpomOR3 expression in neonate larvae, total RNA was extracted from 30 mg neonate larvae using the RNeasy® Plus Mini Kit (Qiagen) following manufacturer's protocol. One µg of total RNA was converted to cDNA using SuperScript® III First Strand Synthesis Master Mix according to the manufacturer's protocol using supplied oligo dT as primer. End-point PCR with Titanium Taq (Clontech) was used to amplify the ORF of CpomOR3 transcript in neonate cDNA using the primer pair CpomOR3ENTR Fwd1: 5'-CACCATGTTTAGTTATGAAAATGAAGACAGCC-3' and CpomOR3 Rev2 5'-TCAAGTCATTTCTTCAGTAGAGGTCACC-3' and the following conditions: Initial denaturation; 3 min @ 94 °C followed by 40 cycles of 94 °C 10 sec, 60 °C 10 sec, and 72 °C 2 min and a polishing step of 72 °C for 5 min. The resultant PCR reactions were separated on a 1.2% agarose gel containing ethidium bromide and amplified products were visualized on a UV lightbox.

Analysis of the 50 most abundant gene transcripts

For each tissue sample, total RSEM gene level read-mapping data was sorted in Microsoft Excel in descending order from highest FPKM value to lowest. The 50 most abundant gene transcripts with the highest FPKM values were chosen for further analysis. The sequence clusters containing these transcripts were extracted from the primary transcriptome and used in a blastx query against the National Center for Biotechnology Information (NCBI) non-redundant protein database. For each transcript, the best blast hit was used as a basis for gene product categorization. In some few cases, the best blast hit was indicated as an unknown or hypothetical protein, but the second or third best blast hit was more informative as to the nature of the gene product. In cases where there were no blast hits found, the transcript sequences were used in a blastn query against the NCBI non-redundant nucleotide database. The Universal Protein Database (Uniprot) was used as a resource for identifying correct gene product category descriptors [3].

An assessment was made of the relative weighted expression of the 50 most abundant gene transcripts by gene product category. For this, the summed FPKM value for the top 50 gene clusters was determined. Then, the FPKM for each of the gene clusters within a category was summed and divided by the total FPKM for the top 50 transcripts to give abundance weighted percentage representation.

Manual editing of chemosensory gene transcript sequence information for downstream quantitative analyses

Each sequence in the Trinity98.fasta file is given a unique sequence identification name, in fasta format, as follows: cX_gY_iZ, where c=component level, g=gene level and i=sequence isoform level. After transcriptome assembly and annotations of the CR gene transcripts, it was apparent that it would be necessary to manually edit transcriptome sequence clusters to facilitate appropriate quantitative analysis of annotated CR genes. In some cases specific gene transcript sequences were scattered across different trinity components or different genes within the same component. Alternatively, in some cases, gene level clusters contained sequences unrelated to the CR gene in question. Additionally, in other cases, ORFs of two different gene transcripts were physically connected in the same isoform sequence, producing chimeric fusion sequence [4]. In order for accurate quantitative analysis of gene expression, it is necessary that each gene level cluster contain sequence information for one and only one gene.

In order to accomplish this, chimeric sequences were first strictly defined as those where multiple ORFs were present in one sequence and there were distinct patterns of read mapping of the input sequence reads to the different ORFs in the assembled sequence transcripts. In such cases, the read mapping patterns were typically identified by the presence of few (e.g. less than 10) reads with minimal overlap (e.g. less than 40 base-pairs) connecting two

unrelated ORFs, as identified with the visualization of sample reads mapped to the transcriptome, with IGV software [5]. For these chimeric sequences, ORFs were split in the Trinity98.fasta files and given arbitrary unique sequence identification tags appropriate to their location within the transcriptome. Additionally, a gene_trans_map file was generated with RSEM software (version 1.2.18) [6], using the "extract-transcript-to-gene-map-from-trinity" perl script, for editing, in order to manually define the cX_gY models for downstream quantitative analyses. For this, components and genes were redefined to reflect the splitting of chimeric sequences, as well of the incorporation of all isoforms relevant to one gene under the same subcomponent (e.g. cX_gY).

Supplementary References

1. Garczynski S.F., Wanner K.W. & Unruh TR. Identification and initial characterization of the 3′ end of gene transcripts encoding putative members of the pheromone receptor subfamily in Lepidoptera. *Insect Sci.* **19,** 64-74 (2012).

2. Garczynski S.F. et al. Application of Cydia pomonella expressed sequence tags: Identification and expression of three general odorant binding proteins in codling moth. *Insect Sci.* **20,** 559-574 (2013).

3. UniProt C. UniProt: a hub for protein information. *Nucleic Acids Res.* **43,** D204-212 (2015).

4. Yang Y. & Smith SA. Optimizing de novo assembly of short-read RNA-seq data for phylogenomics. *BMC Genomics* **14,** 328 (2013).

5. Thorvaldsdottir H., Robinson J.T. & Mesirov J.P. Integrative genomics viewer (IGV): high-performance genomics data visualization and exploration. *Brief Bioinform.* **14,** 178-192 (2013).

6. Li B. & Dewey CN. RSEM: accurate transcript quantification from RNA-Seq data with or without a reference genome. *BMC Bioinformatics* **12,** 323 (2011).

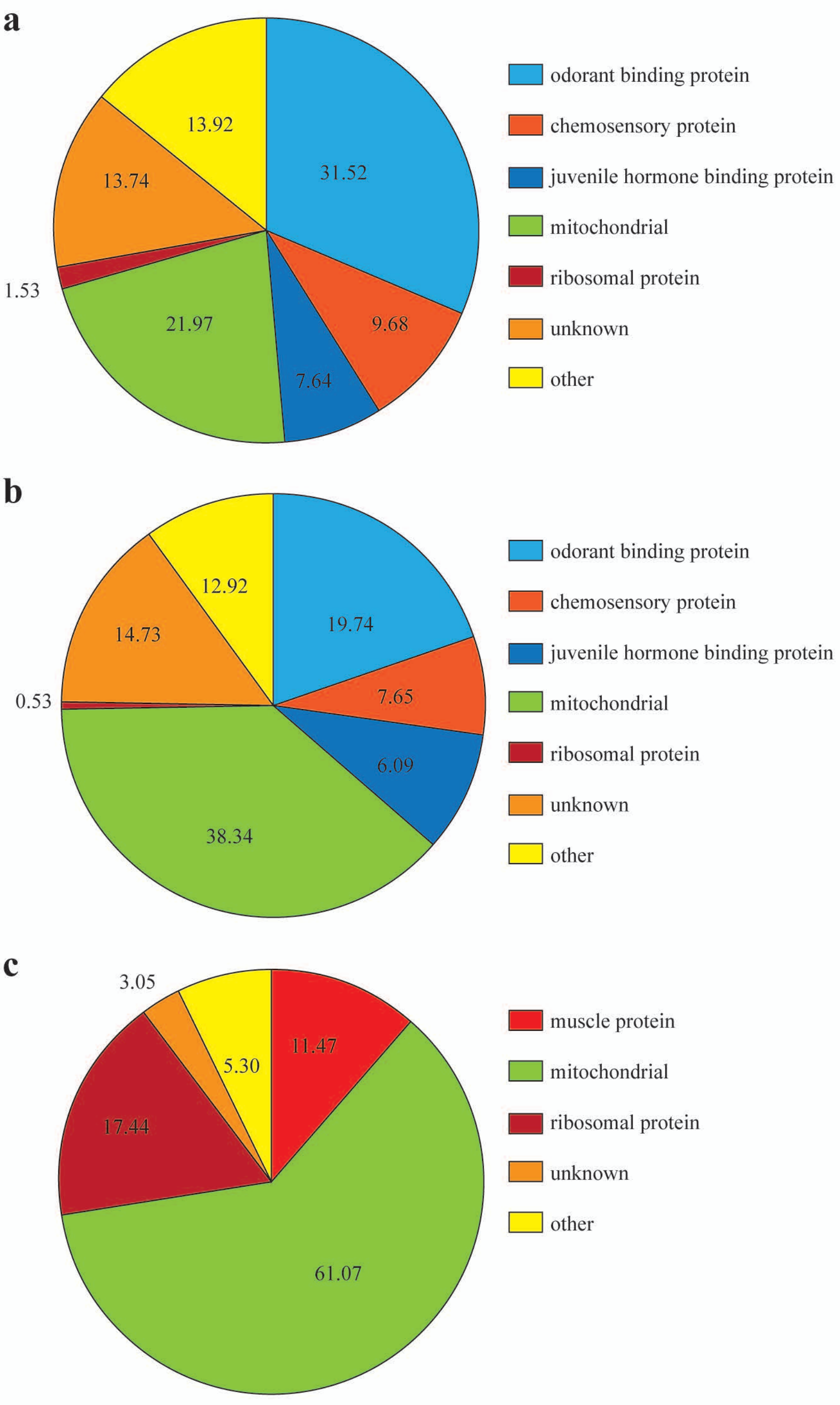

Supplementary Figure S3. Weighted expression values of the fifty most abundant genes in each tissue. Numbers depict percentage share of summed FPKM values for representative genes of each functional category in the fifty most abundant genes relative to the summed FPKM of all of the fifty most abundant genes. Functional categories informed by Uniprot database. a) Male antennae, b) Female antennae, c) Larval heads.

Supplementary Data S4 - CpomOR Protein Fasta Sequences

>CpomOrco
MMGKVKSQGLVSDLMPNIKLMQMSGHFLFNYTEETGGMSLLLRKIYAAMHAFLILLNFVCMGINMAQYSE
EVNELTANTITVLFFAHTIIKLAFFAINSKSFYRTLAVWNQSNSHPLFTESDARYHQLSLDKSRRLLYFICGTTCLSV
VSWVTLTFFGESVRLIADKESNDTLTEPAPRLPLKAWYPFDTMSGSMYIMAFVYQIYWLLFSMLIANLLDVMF
CSWLIFACEQLQHLKAIMKPLMELSAALDTYRPNTAELFRASSTEKSEKVPEPTDIDIRGIYSTQQDFGMMLRG
AGGRLQNFNSTNPNPNGLTQKQEMLARSAIKYWVERHKHVVRLVASIGDTYGTALLFHMLVSTITLTLLAYQA
TKIDGLNVYAFSTVGYLRYTLGQVFHFCIFGNRLIEESSSVMEAAYSCQWYDGSEEAKTFVQIVCQQCQKAMSI
SGAKFFTVSLDLFASVLGAVVTYFMVLVQLK*

>CpomOR1
MSLKSRVWEKLKKKFDDGEVDSPLKYTYVKQVSCLMSSVGAWPHRQFGRQKLHTLLSIYNLGLHGVCAGMF
ILGLLYWRQRRHNMSFFDSGHIFLCMLFDLLVLLRFMVTQTTKYQETIKAFLLEFHLFYFKDRSPYAAKVHTQV
HTISGMFSFYVICQMAHGMALFVLMPCYSNLRKGMFGKNRPENSTFENSAYYYLPDACYTTLRGYWILLAFN
AFSSYIITIGLFEFDLMISMMVFQIWGHLKILKNSLLTMPLPVNSKDGLYSPEENIKIKALLKEIIEHHTLIIKFVDG
CSNTFSEYLFTFYLFMQFITCILLLEVSSFTADALGKYGPLTIGMHQQLIQVSILFEVLNTKSNELIDAMTQIPWE
HMNTSNRRTVLFLICRIQIPVSLKAGGMVPVGVNTMQAVLKGSVTYYMMLKAFAAEG*

>CpomOR2a
MIIKQVYDVLKKRFDDGYVNTPLDFKYVAQLQFVLTTVGSWPYKQFGRNRLAATLSTYNAFLILVSTNLCVLDLI
YIRVNRVKLSFFDLGHNILCLIFTFLYLQRLLTARTSKYQEVIKDYLLDFNLFYLKGRSPYAAKVQAQTHIISGMFTI
YVMWQMVIGVSLFIFMPWFNNYNRGMFSENRPQNSTFEHSVYYYLPDAVYTTEEGYWILFVFNIPISYVTTI
GLCVFDLLLILIVFQIWGHLRILKHNLQNIPLPENSIMYSVEENNNIRMLLKENILHHNIIIQFVDRCSDAFSEYLF
AFYLFMQFITCILLLEVTTFTANSLAKYGPLTVVMHQQLIQVSILFEMLNTKSEQLIDAVYAIPWEHMDTKNRRT
VLFFLHRIQTPVSLKAAKVVPVGVNTMSAILKTTFSYYMMLKALAGER*

>CpomOR2b
MILNQVYDVLKKRLDDGYVNSPLDFKYVASLQFVMTTIGSWPYKQFGRNRLAAILSTCNAFLILLGTVLCVLGLI
YMRESVVKLSFFDLGHNILCLIFDTMYLQRLLTARTKKYQEVIKDYLLHFNLFYFKGRSPYAAKVHAQIHIISGMF
TIYVMWQMFIGLLLFIFMPWFNNYNRGMFGENRPQNSTFEHSVYYYLPDDIDTTEEEYWLLFIFNMLLSYM
TTICMCVFDLLLILIVFQIWGHLRILKHNLQNVPLPENSIMYSVEENNNIRMLLKENILHHNFIIQFVNRCSDAFS
EYLFAFYLFMQFNTCILLLEVTTFTADSLAKYGALTVVMHQQLIQVSILFEMLNTKSEQLTDAVYAIPWEHMDT
KNKRTVLFFLHRIQTPVSLKAAKVVPVGVNTMSAVLKTTFSYYMMLKALAGER*

>CpomOR2c
MNQVYEVLKTKFDDGYVDSPLDFMYVVRLQFLMNTVGSWPYKQFGRNRVAAILSTYNAFLILVGTTVCGLGL
IYMRVNIVKLSFFDLGHNILCWLLEILYLQRLITARTNKYQETIKGYLLDFNLFFFKGRSPYAAKVHAQIHIISGMF
TIYAMWQMVIGVSLFMFMPWFNNYSRGMFGENRPQNSTFEHSVYYYLSPGIYTTEEEYWLLFIFNFSLSYVT
TIGMCVFDLLLILIVFQIWGHLRILKHNLENIPLPENSIMYSVEENNNIRMLLKENILHHNLIVKFVDRCSDAFSE
YLFAFYMFMQLITCILLLEVTSFTADSLAKYGPLTVVMHQQLIQVSIMFEMLNTKSEQLIDAAYAIPWEHMDTK
NRRTVLFFLHRIQTPVSLKAAKVVPVGVNTMFAVLKTTFSYYMMLKTLAGER

>CpomOR3
MFSYENEDSQITSPKDLSYIKQVATTLNRVASWPVLESKNKKYTFHVKWNIICFLFMSIIFILQIWYIRCNISTTSF
VIMGHNYITLAMNIICLQRLTMPWMAEYRQVIKEFLENIHLFHLKDKSEYANKIYKKIEKICYIFTVFVHIQLYCGI
LLFNVTPTYKNFRAGMYGSNKPVNATYETAVYISLPFDYVTDIKGHIFVSFMGWSSTCIGSTSFCLWHLLLSLIVF

HLWGNLKILEHNLDNFPKPANHQMKSIGIAWYTEEESKMISTLIVELVNHHRNIMGFISKTSSAYSFFLLLNFSF
YQIVGCIILLECSTLDTEALGNYAPLTVVLFQQLIQISVVFEILGSQSEKIIDAVYDLPWECMELKERKLVLFFLQNV
QEPINLKACGMIPVGVQTMAAILKACCSYFIMLRTVTSTEEMT*

>CpomOR4
MSPLNFTYVCILSSMLSSIGSWPHKQFGRRRLDFILSMYNILLIVVAIALPSLGATYIWNKRQTISFFDVGHLLLC
MFLEFLFLQRLFMPWTAKYQVIIKEYLLKFHLFYFRNQTQYASKIHTQIHNISVIFTLLMACQMFCGVSLFTFMP
WYNNYNNRMFILDRPANRTFEQAVYFYCFTEDVYTTIKGYWVLFVFNIPTTYNTSCVVAFDLLLSLIVFQIWGH
LKILKHNVLSIFPKEGMYSPEENMKVREILKEIIEHHKFIIKFVNKCSDAFSEELFVFYLLMQVITCTSLLEVSALTA
DALAKYGPITLVVHQQLIQVSILFEMISSKSEQLIDAVYAMPWQSMDTSNRKTVMILLQRSQTPIALKAAKMV
PVGLQTMAAVLKTSISYYMILNTVAGER*

>CpomOR5
MAPPRVFRSLKRWFDDSDAKHPLEFNYVRHLIFLLSFIGSWPHKQFGRDRLHFVLSIFNLFLIVVGITISIAAVGY
IWSKRETISFYDMGHVILCILLETLFLQRLMTGRTEKYGEIVKDLLLNFHLFYFQSRSQYASKVYKQVQFASKIFTI
YVTCHLITGFTLFSFMPWYNNYKNGMFSPDRPPNRTFEHSLYFYCFTDEVYTTLKGYWILFSFNVPTSINTSSGI
LTFDLLLSLIVFQILGHLMIMKHDLLSIPPTTDKYSPEENMRVKETLKGIIDHHNIIINFVDKCSDAFSEYLFMFYLL
MQLLTIVVTVDLSTFTADALAKYGPLTIAIYQPLIQISILFEMISTQSEKLVDAIYEIPWECMDTSNRRTVMFFLLR
AQTPVTLKAAKMVPVGVMTMTAVLKTTFSYYMLLNAVAESAEQ*

>CpomOR6a
MQTKRQTSEPKVFSLDYMKSLRFTLETIGQWPNRSLGDLSRRAVMLATYHKFLICTFCFTEILAFSYMIKHRKTI
RFIDMGQIYTNLFLTGLFLQRASLPFQKNYKKCVKKFVLEFHLMHHEHLSEFAAKELRKVNKICKIATKVIYLQLA
CGMLAYNLSPLFRNYYEGMFAGELPENKSFVHSVDYLLPFDAYRSFKGYLVIFIWNWFPTYNIPTAMGIYDLLV
FVMVFHMVGHMNILLNSLKEFPRPQEDGLPSTREYNEEIFGLLKNVIRHYQIIKDFMGDMTAAFDLTLCCYLA
FHQVMCCLMLLECSTLEPEALVKYGMLAAVIFQQLIQTSVAFELIKSKSKRLGDEVYAVPWEYMNVKNRRIFVL
FLRNVQYPLGLKAGGMVPVGVMSMSTIIRTSISYYIMLATFAD*

>CpomOR6b
MQTKRQTSEPKVFSLDYMKSLRFTLETIGQWPNRSLGDLSRRAVMLATYHKFLICTFCFTEILAFSYMIKHRKTI
RFIDMGQIYTNLFLTGLFLQRASLPFQKNYKKCVKKFVLEFHLMHHEHLSEFAAKELRKVNKICKIATKVIYLQLA
CGMLAYNLSPLFRNYYEGMFAGELPENKSFVHSVDYLLPFDAYRSFKGYLVIFIWNWFPTYNIPTAMGIYDLLV
FVMVFHMVGHMNILLNSLKEFPRPQEDGLPSTREYNEEIFGLLKNVIRHYQIIKDFMGDMTAAFDLTLCCYLA
FHQVMCCLMLLECSTLEPEALVKYGMLAAVIFQQLIQTSVAFELIKSKSKRLGDEVYAVPWEYMNVKNRRIFVL
FLRNVQYPLGLKAGGMVPVGVMSMSTVILYMLAYDGWLST*

>CpomOR7
MTTQTRDRALDLDYMRVIRLYLDTIAHWPNEKFGPKTMRTRILSIYHISILTMLVAVVAAEILALFFVRGRMPT
HGFIDLGQDYLSILIGCVMIPRLTLILQEKYCSHIKTFVSKFHLLEHKHESGFAAKEYQKVNKICRIATMIILLECLLG
QMMFNVVPLYVNIQAGLFTSRDNRPQNVTFVHSLNYYFIIDQYNDAIGYGIASFINAYVSYMCGVEFCGIDLLI
YIMVFHILGHFNILVDKMRNFPRPVNFPDESQKYSERQYNEEALKVLKNLIQHDQLIKEFMNNTSKTFSITLCIC
LLFHQVSGCISLLEISPMTAEALTRYGPLILVLFNQLIQMSVIFELISSKSNKLSDEVYALPWELMDAKNRKTMLL
FLVNVQRPRGLKAGGLVSVGVLTMAQIIKNSVSYFLMLRTLGNF*

>CpomOR8
MLQKMDTGATDVFDVAYMRMIRFSLSSIAQWPYNSFGRKSIRTRIMSVYHYIMISVSTFLEISCVFYVRNNQD
KEFIVLGHDYFTLLMGVVIIQRMTLSFQKRYCLLVKNFVSKFHLVNHQYKCEFAAMELRRITRICNIAAVIIHIQIF

FSMMFFNMVPLWKNIHAGMFSDHRPENGTFVHSGNYLSFVNQYTDIKGYFIVFFLNFYPSYNAAVTFLCMD
LLIFIMVFHIAGHLNILVHDLRYFPRPNEIEQCLETGSKKYNEEVFVRLKDLIDRDQTIKEFMINISETFGISLCIYLA
FHQVTGCVLLLECSPMTPEALGNYGFLTLMMFQQLIQTSIIFEFISTKSDMLADEVYSLPWELMDVRNRKAVL
LFLKNVQPPRALKAGGVVSVGVLTMSTIIKTSCSYFLMLKTLTVEE*

>CpomOR9
MPSYPKDVFSLTYMNRIRFLLNMIASWPNQEFGGSKIKWQAASLYRCLLITFVIFNMTTTISYLQKYVNHDLA
HSYVNMMLASVYLQRLFLPFQKKFCLMIKRFVLEFHLIHQKHKTENTAQVYERVNRICAIVTAVSVTHTVGLPL
FYNGIPLYNNIKAGMFTKHRPANGTFQHSVYFDLPFDQYATLDGYLIVFFYNIYVSYNACIGICMYDALVFSIVF
HIWGHINILIHKLKQFPPTTPQAFTTTVPAAGRQVTQEDMFVRLKDIIRYHQMIKEFMRCTSEAFSISLCCYLLF
HQLSGCVLLLKCSSLDPIALGRYGLLTIMVFQQLIETSVIFELVNSKSDTLADHVYGLPWEDMDLRNRRVALILL
HNVQKSLALKAGNMVPVGVLTMSTVWLGLRR

>CpomOR10
MVSVQKIISLAKRLEDPKHPLLGPNLKGLYVYGLWQSGSKFRNTCYNVIHFCAFLFVISQLIELWIIRHDYLEALH
NLSLTALGMVCIFKAVSYVMWQSDWKKLVEGISAEEISQSDSLNDACIELKQKYTNYVRIVTYLYWNVVVSTNI
TMVSAPFLKYATSSEYREQISNGTEPLPQIFSSWFPFDKTTMPGYSLAIFIHILINIHGGGVIALYDSNAVAVMVF
IRGQLGMLREKCKHIFDEYELVNQEIILGRIKECHRHHNFIMRHSSLFNSLLSPVMFLYVLVCSGMICCSVIQFTS
EEATAAQKVWVLQYTTALVSQLFLYCWHSNEVVVECQHVDGGVYDSEWWKGDTHVRKQLAMLGGKLTHNI
VFSAGPFTTLCVPTFIDVIKGSYSFFTLLTQMQE*

>CpomOR11
MSLLFDESLKSIDYIFKFVGIYLDRTILNTAEHIIKFRSLYVINFLWLNTDVIAEILWIIQGARHGKSLIELTYIAPCTTF
CILANIKALSLLLNDDKVKQLFKQLRDMENNINIGDEIVKKKIVAEEKKFLRAVIKALSVVNALTLILFSLSPVLFM
GLEYKKSGQIELVLPFLIVYPFNPYDIKYWPFVYMHQIWSANLVVTQFAGTDCLFYTCCTSICTQFRLLHHDIETI
IPERNFGENEFLEKFKKLATRHEGIMQSVIQLESIYTKSTLFNFVSSSFLICLTGFNVTAIGDIGFMLSFLSFLLTSL
MQIYLLCFYGDMVMTSSMEVSNAMYNSKWYTVSARAAKHLYVGQMRAQKPSKLTAFGYADVNLNAFRKIL
STACSYFALLQTMESPTQA*

>CpomOR12
APKLSDNSKPTPSFKQSDSFKQNRFCWTVFGLWPGKIPEKYYKVFSFIYLIISYVAYNALLTLNLYHTPRRIETLIRE
IIFTFNETVVACKLSMILYKRKKIAAIFEMLDCEEFKGNDEVGREIVAKHNGYYKKYLLFNTVLSNFTYFSQVLFP
VFGFWIFGNALDLPICKYYFLSDQTRNDYFTSLFLYQSFFMYGHMMYNVNIDTLIAGFMVLAIGQVKVLCHDL
ENLKMEKSIGDQSTTDLKQQYKLRKVLNHYELLLEYCDKFQDVIGGTMFVQYGIGSGIICVVMCGLLLPSSLET
QMFMVGYFMVMNLQIFVPAWLGTQLTYKSEELTTAAYKSEWLPCSKRCKSSIKLLMERAKSPVIITGLKIFPLSL
ATYIQIMKTAYSCFALLRIIQDRQEQAAP*

>CpomOR13
MRPLRQIDCFKVNMKFWKLLAVWPPNDLQSYYRYYQMFFTAFILLNNLLATVNFIFLPRQLDMFIDEMIFYFT
ELAVTSKFLTFLFMHEKIVKILSVLESDMFQPESENGLKTIDKAKKFNVRYFKIVAAVSATAHISHIVPPILLHFILH
VKLELPVCNFSFLSDDTKQKFIYPLYEFQALYMQSQVLFNISIDTFFLGLLIYAIAQLDILNDNFRKVTGKNQIVTR
ADDSIERAEKENTIKKLNDSIIHYGELWQFCFLVQDVFSITLFVQFSVASCIICVVLFRFTLPAPWQYFIFLGSYMFI
MILQILVPCWFGTRIQDKSQQLSQAVYDCDWTAESRYFKSSLRLFVERANKPLSITAGKMFPLSLTSFTSIMNSS
YSFFTLLRHMQSRQN*

>CpomOR14
MDATKAFRTSDSSAVLAPVFEDMEFKPFRETYKIITFNMIVGMLYPTPETAVCRLLGIVLVLISISPAALIALLDV

WHSWQRGDIINIVRHITVLGPCLAAIFKMMLFYYTRDEAWRIIRKIDADHARYNILSESHKEIARRHIQNTQYY
SEKCWAITVAVTVLTFPLTAVVLNFYNFVFKEEPVKYMIHDLEKPFSPPEDRFASPYFEIMFGYMAYCSLWYIISFI
GFDAFFGVTINHACMKLELACKIMEDAMLEEDRDSRQSRMKEVISEQNDFFSMVELIQETFNFWLGLIVIAT
MCQICNCMYQIIEGYGIDPKYIIFILGTIAHIYLPCRYAAKLQVTALDVATHLYCCGWEHVNDERARKMVAFMIA
RAQVPLKITAFNMFYFDMDLFVSILQTSYSLFTLLRS*

>CpomOR15
MKSFRRKESSTLPLVPVTEELEFKPFRETYKIITFTMIVGMLYPTPNTEVCRVVGILTILVTMSPVCIVALLDMWN
SWFRGDIINIIRHTTVIGPFLGAIFKMMLFFYSRKEAWSIIKKMDSDHARYNTLPEQHKEIARRHIQNTQYYSEK
CWSITVATCVLTFPLTAVVLTFYNYTFKENPVKYMIHDIDKPFSPREDRFTSPYFEIMFFYMGYCSLFYIISFTGFD
AFFGITINHACMKMELACKTMEDAMLERDRDSRHRRMLDVISEQNDLFRMVELIQETFAIWLGIIVIATMLQI
CNCMYQIIEGYGIDPRYLVFIVGTIAHIYLPCRYAAKLQVSALEVATHLYCCGWERVNDERARKMIVFMIARAQI
PMKITAFNMFDFDMELFVSILQTSYSMFTLLRS*

>CpomOR16
MVKNKINIEDLYLSRAKFVMSFLGVWMPPPNESIFQKYFRFFMLSLQYTFLLFQVIYICQVLGDLEEISQSSFML
LTHACLCCKITVFHVNIEYFRELLAQMNSEIFMPQTEGHDKILKLQASRIKRLLMGFMVSSQTTIILFAIRALFDD
ANRYFPFKMWMPVSPDHSPQYELGFLFQFITLSMSAFMYFGVDSVCLSMVIFGCAEIDIIKEKIMNVKPIAERL
VNRSITTKNVLDEHYKVLIECVAQHQAIVKFVKQVEDTFHLYLLFQLSAGVGLICMSALRIVVVDWKTIQFMSL
MMYIVVMISQLFLCCWSGHELTATSLELHTVVYECCWYEQDVRFKRALLFTMLHLGRPMEFRAGGYVTLSRQ
TFVAILRMSYSYFAVLQQTNSRNEALELEN*

>CpomOR18
MTVSTVDNVTLFLNRPRNILLYLGIWLKPANYVSLYVAYAIIVMLTQYSFVFFEFIYIALAWGDMDAVTEASFLLF
TQASVCYKVTRFMINKDNLVFLLSFMEEEVFQAQNERHVRCLLNQSIMIRRLCLFFLGSALTTCTLWGLMPVV
DSTGGERIFPFLIWMPVGPEKSPQYELGYFYQMVAIYISAFLFIAVDSVALSMIMFGCAQLEIIMDKVQQIKRV
PMSGKVKKQDREQLIQENKVLFVECLKHHQAVIRFIESAEDTYHANIFFQLSGSVAIICIIGLRITATTPGSVQFIS
MLNYMVTMLSQLFLYCWCGNELTIRSEILREVMYLCPWHEQSNSFRRLLWVAMERMKRPIIFKAGHYIPLSR
PTFVAILRSSYSYFAVLNQTRNKEK*

>CpomOR19
MKNYFILKNLCRKIYLVGAGDFWFEEGEISKGKSLRYQFLCFVLFSIYIFMTVLEIIGVFFGDMPKDERSDCTTFA
VSHTIVLGKMFSVILNRKRVKELNRKLVEICANHEDEHRVAENYRIMKINIWAFAVSVYGSFIFFLFEGIRKMMS
GSHFITIVTYWPFYEDNSIIAVSFRFFTTLVLAVMMATMICIDSFAMIILIMYKYKFITLRYYFEGLRERFDRNNYT
GNEEYATELLHAGFIEGIVMHSNLTRLSKDIDRSVGTVLALQVCLSSGSAVSLLLQLALSKDLTVAAQLKIIMFVIA
VFFLLALFLCNAGEITYQASLLSDSIFYCGWDASSMRRDLRRLVLFSCAAAQRPIVMKAFNMLELTYGTFIQVV
RGTYSVFALISAQNESLAQ*

>CpomOR20
MNQSNCLKYKSFNETFKFCSFALALGLIYPNRKNVCLRTTIFFFVLLFNFGTLFWFIWYTVKCLWELDIYNSTRNI
TVGVIILLFVFKTIYVNLKTDMFASLLEQITKDLLKGNNMDEDYQEIYDYYIKQGLFGQKCYVWIPLILTSIFPTYA
GISMTYGSLRSDDFKKVMLHEMDLKYIEDKQYDFPYFELVFAYYFLGIYILIPNFAGFDGSFCIATSHLRMKIKLM
THGVQRAFTDSKDILELKARLKTCVKDHQEALEFYTLIQRLYGGWLFAVFLLTSFLISCNLYQIYLTGIDPRYTMFA
ATGVFHMYTPCYFASCLIELGEQTCTDIYCAKWESWADPTVTKFLIFIMARAQKRLLLNGLGIVFFNMESFVSL
MQTSYSFFTLITSK*

>CpomOR21

MDTPERRAPRASPLDALSIGYIKILKAFLTISASWPYLTVGGKVHPVYKYYVRCIIPFGLTAISLEVWFLIDHFNVL
SLFEVGQMYLTCFFAALSIARMFLPFCSQYGEIVERFLLSFHLIHFKHKGSYHLKIYEKLEWLSHRVVIITLVLGMF
CAMAYNMMPIINNISSGAYKDDNKTVELAVYFSYPGFDPQDHYKFATVFNFYSVFECAILIAGIDILMSLFVMQ
IIGHIEVLKNSLLTFPEPKKSTNIINADFGRINQFAVLRAPMFTEEENLIIKEKIKDCVKHHLFIVSFTDDMSALFGP
VLAIYLLFHQVSGCILLLEISAGGPDAFTKYGPLTVTIFGQLIIISTIFEIVNTKSELLATTAYSMPWECMNVSNRRS
VCILLRRLQR

>CpomOR22
MKFEEADLIKHVNNNVDIKENLKFEYYLLPPKQQIFYQKLAFAMNVFRMGNQTWWGFPPIHKIFICNTWMV
LIFSPMCLILQFVYMYKNFDDLNFRTLGTMFSIIPATAVVVAKIFICMIPAYPQIMKELMDKIHLNNFIDDEDLFIK
KKLIQVERYTRWITLCLVTFILFDWLLWIFVPLMNNIKNKELIEKRLVRMETCLYLWMPFDYGYDYNTWAITHA
MNVYLVGTGCCVFALYDSINFIFIFHFLSHIDVLRYKIKTYFATKLDESQTKRRIVDIIKYHSFILSTFKDIQAAFGLN
VAINYAHNLIVDSLLLYQIMIGDKANRLSYVIMMQFHMGGLILMSLALEQIHIKTDDLPLLLYSVPWEKMSVPN
QKLLLPILRRMQTPLVFKASGGLRAGVRPLASILKSTFSYYVMLKSSIE*

>CpomOR25
MFGSLLELSDDFFAYNLKYLFLVGLWPDDAWAKTHPSLYKIYENITHVLSIIFLITSGIGTYQIKDDVVLLMTNLDK
CLVAYNFVAKVGIFVWKRRQVEILISEIVNSGDQLTEERKKMMLMIIIVVTGLSTSIVGAFSALALYHNELSVEA
WMPFDPMESKMNLLTASQLLAITFVVPVIWRAIAMQGIVCSLIMYLCDQLVELQDRIRSLEFTSMTERVVREE
FKNIVNKHVRLMGYTQDMNKIFEEYFLIQNLAVTLELCLNALMATMIGFEQKTLLATFFAFLCVALMNAYIYCY
LGNEMIIQSGNLALAAYESSWISWPLDLQKDLVILLRVAQKPLYLSAGGMVAMSIQTYSQTLYNGYSIFAVLND
VVA*

>CpomOR26
MAEYDGELLSYLSLTPHLKVLRNCGIFPLDSTSPNIKKRLHGVYICISFCLIMLYTLLQIIHVFQVRTDIEKVMDAM
FLLLTFLDCIFKQVMFMKKPHKILEILNIMKGPSFNQGLAEHRPLLVRTINHARFLLRLFNKLCILTCFLWITLPIYL
HLNNEIVEFTIWVPFDTNENSKFYIVISYVWMQTTWLGLNNSTMDIFIVYLFAQIKTQICILRLNLENLVSRCQE
EARNTSHSFTQLLELRFRGIIYHYNQIIKFSKINEEIFSNAILFQFLVSGWIICTTAYRTINMNPLSGEFLSMILYMIC
ILSELFLFCFYGNEVAHESQRLMESAYCMQWEELPVKYRRYLIIFMERIKCSILPKAGKIVPLSINTFAQIVKTSYTF
YTFLSKSNAN*

>CpomOR27
MLNKYVARLEDPNHPLLGPTLWGLQRWGMWQPNSGSRRIIYNLIHVAAILFVVTQYVELWFIKADLELALRN
LSVTMLSSICIVKASTFVVWQTYWQDVVQFVSTLERSQLEKKDKTTCTIIERYTKYSRNVTCFYWGLVVATGLM
VIFAPLGVFLSSSELRELMLNGTIPFPEMVSSWVPFDKTRGFGYWFQIVEHSAICFYGSGIVASYDVNTVALMS
FFCGQLEILVANSKKLFSEDGKLVSYSEAMERIKQCHKHHLSLIKYSKILNSLLSPVMFLYVVICSLMICASATLLTK
EGTTTMQRMWVAEYLAALIAQLFLYCWHSNEVYFMSESVDRGIYESEWWQCGVGLRRCVVLLGGQLRKTII
FEAGPFTNLTVATFVAILKGSYSYYTLLSNNEG*

>CpomOR28
MRVWARRKSATLAGASVVSLLRTLRWCGFCRLPPSAASGPQTSLLTSIARATHDVYCGFALVVTSTYLVQELIYA
YLERGDMDTLARVMFLLLCHVTSIAKQIVFMARASRIAKLVQDFDDMAYNPEETTRENLLIERAQGASRLGAA
YAGTAALTCALWTIFPFLARLGGTRVIFALWVPFGYYSWPEFLIVLLYTYYVTSLVGIANTTMDAFIATILGQCKT
QLTILKMDFESLAERANERARETGEQVGAAATALLVRCIKHHHKICDTSREVQAIFGGAVLLQFAIGGWILCMA
AYKIVGLSVASLEFVSMVMFLMCILTELFLYCYYGNEVAVESAQVSDAVYGMEWVGPNGVGKEVRRALPFVIC
CSVGAARRPLRPAAVFIPLSLETFVTIIKSSYTFYAMLRQTQH*

>CpomOR29
MIKEFLENIEDPNRPLFGPNYWLLNKIGLLLPKNRLDRILKIIIHEIATFFVLSEYMELYVIRSDLDLVLTNLKISMLG
IVIVFKSNTFVFWQGNWRQVIDYITEADKFERDNQDEAKGNIINTYTRYCRRVTYFYWVLVFTTFITTMATPL
MKYYSSETFREGFHNGTEPFPHIFSSWMPFDKENSPGCWITVMWHTGICAYGAMIMAAYDTSVVVILVYFG
GKLDLLRIRCREMLGTEEKGVSNENADKVVQQLHQIHVLYLKHSRLFNSVLSPVMFCYVVMCSLMICASAFQL
TSATNTTQKLLMAEYLVFGIAQLFMFCWHSNDVIHKSQAVMNGPFESDWWAANLQQRNNVLILQGQMGI
VHIYTAGPFTDLTLATFVAILKGAYSYYTILRK*

>CpomOR30
MSKILDEDMNFDKIFWIATTAMRLNRSHPYVARDKIWRNQFIAILLLSLFCFIFLLYSILFHDIQCGLFADASKNA
IMAIVAFTITYKYYILLRYQDSVTELIRIVDDDYELAKEFCEEEQRIVLHYSKRGVKVCQYWFISACSTSAIFPLKAL
MLMGKSYMAGEFQLVPLFELTYPWILEDYKTVPIIFITLFGLALFFDVYATSMYVGFDPIVPIFMLHLCGQLDILN
LRISKMFSDTEHSDEAVRKNLTEIILKLQDVYKFIQVIKTNFTVLYEFMMKTTTFLLPLTAFQITESLRNGEINIEFI
GFFTGVILHFYIPCYYSDLLMETGEKFRLAIYSCGWEKHSDMRTLPTILFMLTRAIKPVVISTIFCAICLDTFAQM
CREAYSIFNLMNAAWA*

>CpomOR31
MSKILDENMKFDNIFWIATTAMRLNRSHPYIPRDKNWRTQFTAILILSSFCCMFLLYSTFFHDIPCGAYADACKS
TIMAIVAFTITYKYLIMLRYQDSITDLIRIVDEDYELAKGFCEEEQRIVYKYSKRGVKVTQYWFVSACSTSAIFPVK
AFVLMGKSYLAGEFQLVPLFEMTYPWILNDYKNVHVVFVMLFGLTLFFDLYATSMYVGFDPIVPIFMLHLCGQ
LDILNLRISKLFSNTQDSAETIRENLRRIILQLQDIYKFIEIIKNNFTVLYEFIMKTTTFLLPLTAFQITESLRNGEINLE
FIGFFTGVILHFYIPCYYSNLLMETGENFRLAIYSCGWEKHWDKHVMRTILFMLTRALKPIVISTVFCAICLDTFA
QMSREAYSIFNLMNAAWA*

>CpomOR32
MMKQIFMNLLSRYIPVWNQKNPSIANTALRLICNTGIWHYQSLGLHWVAKFAIICFISTNLTQVATLLIERDDST
RMFETFSVLSFCGMGTLKLFNLYTNRKRWTSIISQLQCIEHEQLHGKLLSCIDSDIEEDYSPQIIAKYTQRHTFISS
VLLRLYSITAIVFIATPFVEYAVTADASYFPHILPGWAPLDNIGFAGYFLTLIFEIVASVYCVFIHVAFDCTSVGIMIFI
CGQFSLLRRKTEDIAGSGEDCMPSTMRDVRAHLKIIESHGTHIALRTVIKELDTVLRGILGVYFLVATLTVCSVAV
RLNSESLSFMQLVSLLQYMAGTLTQLFLFCRYGDAVFHESSFNMGEGPFGAAWWSLCPRMRRQLAMLGAG
MMQPRSLHAGPFNRLDLPSFVQIVRAAYSYYAVLGQTSK*

>CpomOR35
MSIEGYKLVRNKKISRFSLQNMLRCLEDPKHPSAGPYLRFLNLTGNWHPNMELKSTRFKQLIYYMTMAFFFSQ
YLKCVISLNLSAVLFILQTAPFHMGTPKTIYFRKDYHLWEKLIDYISRTELRQLSDGDVEVIDVMDEYIKKGRRVIY
PFWFMVICCNISIFTEPYQKNQMVENGTDIYVPLFHFYVPFNQDIPPGYYYSMVLQTILGNIMSSYIISWDSLVI
STFIFFTGQLKISRVYCTKIIDPESKERSHENIIKCHRFHTALIEHQKLFQKLISSVMFLYLIVISINLGSCIIQISNASG
DLPVMMGAMLFVFGILTQLLIFYWFSNQVTVESLSVSSGIFESKWTTMDAKTQKEVALLQLTTSKRLCFRAGP
CNEMSLDTFIAILKTSYSFFTLLKETK*

>CpomOR37
MECFSRAQDGFHRLKKRLRENSFDNLVCLVMVMPSLVGFEITRKKIFVPFWIIHLSLLTYVYGVGSLVYQAKHA
RVASDFIKSFVNVSILVLTVNNSYWWLTHRDLLRNVLRKANASDKMTIQAGLFVDKHQHSLSIIKRILIIFYFINLT
NEFTSYLPKRAELNEKTFSMTPCVGIKPLTSSPQREVCIVLTSLQELTIVIVVLNFQTMMLLLIAHTSTMYQLLSD
EIMTFNTILTNPSNYDLLKERLGVIIKRHILTLDIIKDIRVLYSIPMGINFGSNAVCMCFFFFLEPEEYFNFMPISMY
CFIVFFLYCFLGQRLTNAAEVFSRAVYSCGWELMDIKEQRAISIMLLQSQKEVDLLAADLIPVNMLTFASTSQGI
YKFVTVFKL*

>CpomOR38
HHHDLPTMLWNADVLLRVMALDVDGRNNKRIPIILYLTSAIVLVLYFYTYHLSTYWYIFWRGGGDMLFIILLVSL
SISSSIGVVKLLYMYFNGTKLKKLVLEYLECDAALAPDSRMFRNVNATLRSVKKRAIIFWMIIIGNGVVYVGVPLL
KPGRHLTEDEQILLGLEPMYESPNFELANIALFAGVFLTVYAPANITGFIIIIVGYSEAQMLALSQELLHLWDDAH
THYKQIQSHSTDDPDCSNGISRELEAIKKNKLRMVNIYVKNRLVSIMKSHATNINLINQVESIFKSAIAVEFALLS
CGLIAELLGGLENTYIEVPFALVQVSMDCITGQRLMDASKAFEDAVYACKWEHFDVRNRKLVMLMLQNAQK
TLRLSAGGLASLSYTSLMSVIKSIYSAYTALRSTMNK

>CpomOR39
METLDKFGLAHCDLPTMMWNVAVMLRVVAVKIEGGATSIPIFFYLLATVAIVLYFFNYYASMLVFVLVGCRETG
DILAGIMVLSISMNSLIGINKLFYIYRHQDKVQSLVADYVAYDRIAPWPGTSALMAELMRSVKKRLILFWTVTM
GNAFIFNLQPLVMPSRLNLYDKHVVYGLKFILGITNYPIAIVNIVACTFFICYITSSIGGLLIVTTGYSEGRLLALSQE
MRDLWSDAHKHYSENFEGDSEDKKAAKELNNYVKYRLQVIVKSHAINIDVIKKLEGIFRNAIAVEFILLTAGLSID
LLGGLEDTYIILPFSLMQVGMDCYLGQKLMDACKVFEDAIYDCKWENFDVKNRKTVLLMLKISQRTLSLSAGG
VATLSFECLMAMYKAVYSAYTALRSTME*

>CpomOR40
MLNLDYDKMFKISILALKLNRSYPTIPKDKFWFCSIIPIHAYFSLSFCLILYSMFFHDLKNNDFAAACTNGILSVLYI
AVTFKYVVLLVKVEDITFAMNKVKGDFAAAKHLCSDEQAITTEYAYKACWVTKVWLLTASSVFCVFPIQVIVLSI
YNYAIGDFQFVHMYQMTFPEAIETRKYETNMYLFLLILQTYFGVYVLLMFAGFTPLGLIFMLHVCGRIEIVKYRI
SKLFEGEHYDPREIHQRLKNIVTPLQDALDFVDLIKKTFRLVYEVYMKFTTIVIPIASYEVLESLKEGRLSIEFMTFI
VAGAVLCFAPCYYSDLLMEKGLSLRMSVYTSGWEAYPDSAMRRTLCIIMCRLERDVAIRTLFQTVNLDAFSELC
HQSYALFNVINTAWS*

>CpomOR41
MDVRFDNTFKLTSAALYLNLAHPFLTRNLQWAIRIFIFYVFYTPSFIFLIYSSFYDTFTKICTNLSISVLYCINLFNYGL
LIIYKKPFMDMIKIVEENLRNSRELIDEDEKTVKEFTAKGIKAAKFWTFCCVLVGVMFSSKAVIGTSYSAFTGNFK
PVAIHELTYPAYIEERKNGFLMYIVIFGFHTFYIVFTILMDAGFSPLGPIFILHACGQIQVAIQQVERLFLDNDIDV
DDILNKLKNITRRLQNIYSFVDQIQYTHRLLYEMCLKASTIFVAISLFAIIESYKEGSLNFDLLCYSFSALLLCGIPCYY
CEALLGKGAELRVAIYECGWERFWVPKSRSIILVLLTRTVRPLGIKTVFCTLSLEAFGDVINQVYTIFNVMNAAYN
*

>CpomOR42
MSTPTFYEVFRQIRINLSVMGIQEGKSRTGVIFYIFYAMLFTMVSSEVVFFTANMAPENFLELTGLAPCICVGILS
LLKIAALAWKKETVFSLAHKLERLSTETLKDPIKTDIVSPDINLLKTLIKYYFILNAVLICVYNFSTPFYILYHYLTTNE
EIFILPYAVTVPFSTEAWPGWTFVYVFSVICGFICVLFFTAVDALYFTLTSYVCTIFAVLSNEIICLNQPTGDILDQIIK
KHQNVLELAEDLEDIFTLPNFFNVLVGSLEICALGFNLMIGDWNNVPGCMLFIMSVLFQLFMMSVFGEKLIGS
SIKVGESAFLCDWYKMNQKTQKVLLVLITRTRKPTRLTAFKYSVICYEGFTKIISNSWSYFTILRTVYSPEDQ*

>CpomOR44
MKILSEYMREKLSFLTPCLPYGVLESWEDLNPRLYHAVHIYWLKFYGLWYNTHPKTSLLFWAHIVYAVVVLWLV
CFLPGIGEVFYLLKRRDNIGDIAEGLYLFLSEMYTYIKLSVFWLKRKEIMALLEYLHMDEFKVKEPEHRQILRKSIK
RARFVMTYYSSMCVGAVSVGILMPLAEQFEVLPTNVEYPYFDVYKSPAYEIIYIHHIYYKPATCIIDGVMDTILAA
FIASAIGQIDVLAFNLRNFNLLAQRRREMLPFTTVNNMSPKHMPLVDHEKTRHCVRAVFKDIIKHHNSIIKYVS
LIESAFSLASAVQLMLSVMVLCLVGIQFLSIEEPSSHPIQIAWMAIYLTCMLIEVFIICWFGDELIWKSWELHQA
AFDSPWPSTDPKTAMFIVIFMERCKRPLRVTAGKIFTLSLDTYTNLINWSYKAFAVMRKMKK*

>CpomOR46
MGIIEKNVNFSVSVSITALKLFGFWAPEGLTREQNILYNCYGFLSFMFLLGTYLIIQVVDLFLIWGDVALMTGTAF
VLFTNLAQTTKIVAVVARAGTLRPLVSSADTLLAAETGPGKEIVDSCNRETWQQQLVYFCLTTVTVAGWAGSA
EKNQLPLRAWYPYDTSVSPAYELTYLHQVGALFAAAYLNVGKDTLVTCLIAQTRCQLQLAALRLASLTDDLTPTA
QGTLTAEQEAEAWSRLEVAARRHQEALHAAAQLQACFSAPVFAQFGVSMLIICVTAFQLTAQNGNLVRLASM
GTYLLNMMFQVFIYCYQGNQLSGESMDIATAAYSCGWTACGVRLRRGLLLVMVRARRAARLSAGGFATLSLA
SFMAIVKTSYSLFTVLQQADEQK*

>CpomOR47
MTNIVSSDRPSRYFGVHYGLLRFLGLGWWHHPDEGDFRNFPSWYLYYSILTQVVWVAGFVGLETIDPFVGEK
DIDRFMFSLSFVITHDLTCIKLYLFFFKNRAIQEIVRTIEIDVYDYYQNVDKNRRTIRITRIMTASFVFFGWITIGNT
NVYGTIMDLRWKREVALLNGTALKPPRTLPQPIYIPWAYQSDESYIATFVLETVGLLWTGHIVMTIDTFIGSLILH
MSSQFSILQEAFMTAYDRALSQLISDMPLDIDTQDELINRNIYILKHRLDEIEAKVKSFYAEIQIESAIEKSVKSCLR
QHQLLISCVEKFRVTYSYGFMTQLLSSMAAICVVMVQVSQDASSFKSIRLVTSLAFFMAMIIQLAIQCFTANEL
TLQAERVSDAVMQSKWERMSPRVRRYLLMAMMRAQRPLRLSAAGFAYMDNRCFLAIMKAAYSYYAVLSQK
EV*

>CpomOR49
MIKYILKKLENPKRPLLGPNVKALQFWGLLLPENVIMKYVYICLHISIIFFTATEYVDIWFIKSDMNMLLENLKIT
MLASVSVVKVSTFLIWQNSWRDIIDYVTEADLNQRKTTDETKLTIIKKNTKYSRKITYLYWSLMYTTVVVVMV
QPIIKYVFSQTYRDNIKNGEESYIQVVSSWVPFDKSEVIGYLAACAFQSYAAIYGGGWITSFDTNAIVTMVFFKG
ELQLLRRDSAEIFGIENNPVSREEAEKRLKECHRRHVNLIKYSSLFDSCLSPIMLFYMFVCSVMLCVTAYQIRYGT
SMMQTILQVEYLVFGVSQLFMYCWHSNDVMYTSENVIHGPYESRWWSENALRKDLVILLGQYRKEIVFSAG
PFTNLTLPTFISILKGAYSYYTLLTKSRTDI*

>CpomOR53
MKLTQCFKVSYFWLILGGVWYPRSLDNSRMLYAVNFYRVFATIFINVGMLAIQFIYFFTVVGKDLDKTVDATAL
FTFVGYLYKAITVIKNRQRINKLLDIIDNETDKDDLLRNMAANINFVSFFYNGWACLTAIMWNLIPFTKATLTLPF
YYPDLTPSSPWFVTWYIYQATILIINGVAQTSADHLFGGLMAFAATQLKLLQHKLEAIGTKTDSTMEIDAQRQQ
EDYEETVSCVEYHLKIIWFVDELTDIFGGAAFGQFLLAAPLICLSMFIIMTSSDVTEIVTRILYFGCLSGQLFIYCFC
GNLIKTQSDLVATAAYKSHWTSTSVRTQKALHLLIIRGQKTMSVVAGNLFELSLVTFGALLKSSYSFFAVLSKQRD
E*

>CpomOR54
MKNSDCLASSIAVMKYTGVWMPDNLTYGGRMTYMVFRCVTQTFIFVFIILAEIAYVYKHRHDSERMVDAAVL
LLSHLVQAVKLMTIIVRQERIKRLISLGDGPAFTPTEPKLKAQLERAVKLTGLIGNLVLWSACITGVFWFVVPALK
DVLTLPLKITFPFDISGQYIFAVMYVYTSLSVLTCGVGDAAENFLVSGVLTLASTQVGLLHEQLLDLKTDGKDGCY
KKAVLCVKFHQRIIEYVEEVAKIFGLPIFCQCVTSSIVVCMTVYKITITQEPVEMVTLVFYLICVMMELMMYCYP
ADVLLNKSLQVSDAAYPEWSGNIKTAQVLLLTTLRAQRALVVNAGGMFKISLPTAAAVVQTSYTYYALLQQKLK
KE*

>CpomOR56
MERWRTYSLEYSDMPTMVANVADLLKHLGLNIDGKVRVIPILSVIWTVISVVLYFYVYVFSILWYVFWRGTGD
PVVDAVLLSLACACIIGLLKLFVLHFNRKSLQYTLISYLSYDKRLCRESRMYRRLVKNLRVIKRRASAIWILLVVNG
AFYCFMPLLLPGRHLAEDMQVIYGLEPMFESPNYEIAHILFWITIMVTVYCSGSIAALLITLSGYIEAQMIALGD

EILDLWPDAQALTCETGQDEDKLRNLYIKHQLEYIVQAHAANLALLRLTESIFSNAIAVEFCLLGLALIAELLGGIE
NTYIEIPYALNQVSMDCFTGQRLMEASLRFSDAVYDCKWENFDASNMKTVLMILKNSRTMVLSAGGVAELR
YTSLMSVIKSIYSAYMALRSTVD*

>CpomOR57
MTTTYSTFEAFRPHFNALAYVAYFKIIPKPSSGVKHTLHTVYRAVVWFLVIIYNLQHVIRVIQARHSTEQAVNTLF
VLLTTINTLGKQVAFNSRVERVDRLVATIEGPLFMARNTYDEKVLRSNAWIMSRLLMMYHGSIYLCGAMWGI
SPLVSKLSGEVELTGYFPFDTSGWLGFGIAVAFNTIVITLQGYAHVTMDCTIVSLHAQTKVQLQMLRNSLEHLT
DSVGKTGREICVQSTVYKDIEDTAFGVVLKKRLTRCVEHYKLIVWFHSEVEAVFSEAMIVQFFVIAWVICMTVY
KIAGLSLVSAEFFSMFVYLGCMLGQLFIYCYYGTQVKAESEFINYSIYRCDWVSLSPRFRALLLILMSRGMRPVA
PRIAYIIPMSIETYISVLRSSYTLLTFLERK*

>CpomOR58
MMPIKPFQNNRTSDLFHTICKIIYLSCATNFWFEDIDYPAIFMKIYNSTSRVLEVTVAVLIISDWGAFWTQPNLT
EKQSNDRMLFAFSHVVLYSVYCSVIYYKREIRELVMTLTVRLKEVCYDGSIEKMMLRTTFRYTTAFVFVCSSTLFS
FGIGSGFQALTTNATFTTIIPIWPDVEDRRLVAGAARIILYIVWWIFLVRFISVYIILLISTIGIAHQFKNLCKYFEDLT
DIFEGSGSQEEKERRYENAFKVGIKMHSITLWCMRQIQLVGGVAFSGQVIINVSVLGLLMIQMMFTERTLIAV
MPIIFMVSSVLVGTGVFLWNAGDVTIEASRLPAAMFHSGWHNCTRQSSVRVRKLVTIAIAQAQKRVRIKGLGF
IELSYESYVTIVKSSYSLFSVIY*

>CpomOR59
MNTNAEARREIGATLTLCTFSMQCIGLSFERPDGTARLLRQKLMFVVSVCTIVYHVFSEIVYIGLTLSNSPRVED
VVPLFHTFGYGALSIAKVFALWSKKNVFTEHLDELSGIWPMEPLDEDARNIKEKSLTALRLVHQWYFSINVGGV
LFYNVTPICVYMYQLWQGQDAVVGFVWMSWYPFDKYKPINHVFVYIFEVFAGQTCVWIMICTDLLFSGLAS
HIAMLLRLLHKRLETLAETEKSQEEYYQEIVANIKLHQRLIRYCNDLEEAFTIVNLINVVFSSLNICCVVFVIVLLEP
FMAVSNKLFLGSALIQIGMLCWYADDIFHSNADVALAVYNSGWYRTDPRCRRALIFLIRRAQKPVAFTAMKFT
NLSLVTYSSILTRSYSYFALLYTMYNDS*

>CpomOR60
MKYKPKISNSSTRYFQKLTQFVFIVTATNFWYKDVKLPNRFVKIYSHVSKFLEAIIITFVITGFGTSYTQKNLTPKQ
SADILMKSVSSFFVYTMYGFIVYNKEEIKELLFSLTVSLMEIYNDKIEKKMMMKIRIYVAGLMFVSSCPMIAYGV
EGAFHVLTSNATFTTVIPIWPDLEDRRLVAGFARILIYIIWLLLIAHVIATYCLMICISICLSYQFANLCEYFLHLNNIF
NGEGSQEYQEKRYEKAVKVGIKMHNTILRCVNQLQSSCEVVYGGQILINVCVVVLLMVQMMQSDRSLVQLA
PIVLSVTGVLVTSGLFIWSAGDITFEAERLPTAMFHSGWHNCRRQSSVRVRKLITFAMIQAQHVVIIKGLGVIEL
SYDSYIAIVKSSYSVFSIIY*

>CpomOR61
MSSSAKEEFLAGMDYLSVITSRIFLYPFLGRSKTKLLCYYFICSLIIFASFQQFVFLCVSKLNSFLDIVNIAPNIGVCA
MSVTKYIKVNSNKELYNLIFVHFRTDMWDIVSEKCQENVKILKRYQKIIHFITIWFVYYVVPLILIVTSFPILIMYY
DNMVLGKELEHRYPFEAWYPFDKVKWYYAAYAWESFITGLVVCIYTFSDLINVSYVAYICLELKLLGTHLKELIGA
EDIKQLKSSQNATAIHYKIRQKLRGYIIKHNFLANISSQLDIIFGDIMLVNYTFGSVFICLTAFTFTVTDELYSTLRCF
FFLISLVISMLNQCVIGQCVSDHSEQLTQALYDSKWTYGDRQTRQLVLMLIMRMQKPFQLTAKGYIAMNLDTF
TTICSTSYQFFNLLRTMYDPKAN*

>CpomOR62
MVFLTSLWRAITHTKALEESSGEMETTFFETVYRITYIAGLSRSDHSFFYKLYSNTVKLMIATFMLGEVWYMLTY
VSSLDIVIEQMNVIVIQGMALFRYRYMRMHERVYKRLATSMQISNLDTSTPARKALLETWMKRSETYLKLML

GLGSLTLAAWYVYPLVDDIEYNLTVGLRLGVDFSRPSRYPIAYTIHIVAFHYTAFFIIVNDVIMQAHLIHLVCQYTV
LADCFENILVDCEKHFKGLTRDQLVRDSRFREVYISRLGLLVGQHKKILMHTMELRKTLSPPMLGQVAASGLQI
CFAGYQVAMTLTVSFTKFFMSLLFLGYNLFELFVVCRWCDEIKIQSENISNALYCSGWECGVATMPGVRARFLL
VLTRASKPLVLTAGGITDLSLNSYSNLVKTSYSALTVLLRLRHE*

>CpomOR63
MLEKLKRLYNKYDFDYSTGQVDPYKFHSTFYFILKAFTVIDEPLPMWSYVSLAVNVFDATVAVFFAGIATVHGIS
LLDISITTEAGVYCIVLIYKCLILTCTQLDKAHYHCFLRVLREDFRYVCAEGAKYRERFFENQLETWKVSLCSVIFTF
GIAVGMASFALVSLLFYLMTRTPGDGSQRPLLVPFWFWDLDFGKTPIYEIALNFSNFCFVTYAYNYVFMIQTQV
VWVRQIATKADLVIWAIQDLLQDIHPATNEKEKVHYAELIKYRMREIVSQHHSMYTLMEAYAGVYKKLLMFEQ
KLCGPVVCLTAYCTAEKLDEGEFNAILVLLCIATVTLVYIPCYLCTFLGLKVRSVSDACWNISFWNAGREIRPYLVLI
MQRSLRPLPLQAPGFEEISIQTFSTKMTNAYSLFNMLRQTNI*

>CpomOR64
MKAFVSTAKTFLYKNDFEWNKEITLQNFHPQLQIFLAINGVFFNNRESKIRFILPVLSTLITLVAVAFEIFFIWHGI
SMNDYGFATECFCYFFILGSVGIVYSSVLLNRVKVFKLLHNMNNDFLFICNLKAEYRDTFLTGQLLIWRLCWSW
IVFISFVSLLYISNTLLYLLYQSTLATQDEHMIRPLIFPMWLPEDDPYRTPNYEIFLALEVILIFVVLVTFGLYVYILFH
LLLHYYNLMDVILIALDDLFDGLDESVVALNRGDPRRQAVQDELNIRMGQIVRWHLSVFDSVDDISSVYGPTL
VYQVMFSSIVICLMAYQVAEQLSEGKVDYLFGILGIGACLQLWIPCYIGTLLRNKGFFVGDRCFYCGWHETPLS
RLLRPDLIIFIQRTQRPVAIKFTGLPHLQLETFSSIMSNAYSLFNMLRQYK*

>CpomOR65
MTLLTIFRDIKSFVNKDGYDLERPDVTLQNFHPQLEVFFAIKGIFFNNCSSKKRFIWPALSSFMASVATGFELLFI
WRALTIKNYAMATESFAYLIILGSVILTYLGVLTNRTTILTLLSEMSKDFRYICNLASNYRKSFLDGQLLIWKLTMT
WGIFVICVAILYVLNTLLLLLYQSLFATLDEHYVRPLIFPVWLPHDDPYRTPNYEMLLVFDIAIILVAMASFGLYVPL
SLHLFMHYYKLLDMILIAIDELFEELDESVVTLPVTDQRRLDVKAELSRRMGRIVRWHQSVFDSVGAITSIYGP
MLVYQVMFSSVIICLMAHQVAIQMADGKFNYLFALLTFGAILQLWIPCCMGTLLQTKALSLGERCFYSGWYKT
PLTQLVRQDLLIFITRTQVPVEIKFTGLPEMELHTFSSIMSTAYSYFNMLRQYN*

>CpomOR66
MESREYRKNKTTELFHNLDKVIYVFSGMNFWVDDNNVPKLVFNAYKRISKVINVAIVLFLMAEIGSFFTQNNL
TEKQKADRVMMTFSHIILYYFTHSLIHRKETVTEILYTLAVSLKKDFNDEETERLMLKRTKIYMCVFVALCCISFVF
YGVEGLARVLFSGKAIYTILLTNINTSNLSSDKKIVYRSYVFQTNAT

>CpomOR67
MMWLKSHPVITSTFTGHLTFYHTMEHLKDFASEFSKPFAICFDLLAKSNISIYNESKIRGKLRILALVVFYFTFYSSL
VVSFKKVFTGELGFYELANLLPIFIVATQGAMKGIVIVSNLSKAKTVIDELGSMWRTTGLTKTQLMKKGVMLKR
LNLCNAVFYWMNITGTWQYILVPLFETLFRNFVLGQDQLLFPFLCSLPYDAKRNWMVYLGTYFWESYSMLHL
IYMYLGVEFLMITLCSHLATEFELLREEMLHAKPILEHSENTSYKDHIGRTLSYSNNEDDDAIDYFEEDSNENEV
RADENGPNIQEVIRRHQTLIKLSELLDDIFNRMIFFNLLFATITICFFGFVAKIARDLPEMANNFVGVVASMIPIF
NLCYYAELLSGASAGVADSAYHNLWYEGDTRYQKIIIFIIVRSQQPCCLTSMRYAQVTLNTFTTVLSTTWSYFSLA
ISVYET*

>CpomOR68
MASLDHLPSSFIDTIIVPVKLYRFIGLQFFDDDRARFKNYCKLILFILLSFIFSCGLILFFIKINEIDAGILEIANATPCLC
LVIQSLLKLSLLRKKHLIRSVVYEIAEMWPGEMENREQKELMDNWLHRNKLICDSILKFTIFGLLIYNGVSLVIYFI
LRILDKNPAYVFPFELYYPFEIDSVWKYVAVYLMHILATTIIYECSYLSCDMFLFSLTVNVSMLLRLLHYDLVNIDV

RRRGQEADESLANLKNIVKRHQKLLKLAEDLDRIFSAVMFTVLVFSSLIISFFGFLTIVIKGKFQQFMNLIAALEVL
FSVFYIMLPGQILSDTSSGVADAAYQSLWYNSDERFRKIIIIMIARSQKPCILRAMGYADINFETFYKICGTTWSY
LSVVNQMYQDSL*

>CpomOR71
MVHLKNVSFWRKKEYGVRKRYYDLEDYDATFAIPWSVQKWVGLRLTKTDPPHVRIFWDTYYWLENLNLWLA
AVLELIYMVVRSTEYVEIFLSMPCLCNLLLAIFKSYKMVVYRPVFNNLVWELRTMWPQGTVTEEEDRIVSRTLR
SLNMVVKGYYWCNILLVLIFLSPSFVALGYRAAGHDTPLILPYWYWYPFDPYEGGLGYAFALAFEDFHGCSAIC
FMVMGDLLFCIFLSHISIQFDLLAVRIQKLVPTIEPKHRLSAFTTEQMRRENCNSPEWEKTHLKELAAIIDRHRA
LIRLSGDVEEMFSGALLLNFLNSSMIFCFCGFCSVIVEKWNEFSYKSFLVTALAQTYLLCAHGQKLIDSSKGITNA
LYNCLWYNASKKVKGSVLITMHRSQKEIHVTTYGFSVINMASYATILKTAWSYLSLLLNVYK*

>CpomOR72
MSEFTNFEPMFQETYKFILDRIKSNQIYIMDEVSWRGHLCWIKLVVIILAAISHTAGVFERIGQGADLVELSTDL
SAVLILWQVTLLYIQFCLNRKLLKNFILHMGSNWRTDDQLRPDMIAVKHDYVTTFLSWITVFYKAVNIYLFLYL
MPRLLYIAVKHFILKDSVAFVTPFYVKMPFKFDDNFLLYCLVYLADSKILQDVGYLVTFDLLFMNAAMHHLRLM
FVMLQADLRHLHDVGVEQAENTLKKLIPHHQNLLNLMVELSNAFGAIFIIHLAFFSGTMCFFGFAARIHCSPES
IKNLFAANIILVCIFTCCYYGQNLTDASVDIAQAAYESQWHLKSQEYKKCILFIMLRSQKAQYIKSTSFTDVSLQTF
TKILNVTWSFLSLITKVYEA*

Supplementary Table S5.

Novel Chemosensory Gene Informatics

**Gene Name**	**ORF Status**	**ORF Size**	**Best Blastp Hit in NCBI nr database**	**E-value**	**Identity**
CpomOR5	Complete	426	*Cydia pomonella* OR6	7.00E-163	64%
CpomOR7	Complete	422	*Ctenopseustis herana* OR6	5.00E-103	44%
CpomOR8	Complete	419	*C. herana* OR45	1.00E-104	44%
CpomOR9	Incomplete	403	*Planotortrix excessana* OR1	1.00E-105	44%
CpomOR12	Incomplete	405	*Ctenopseustis obliquana* OR12	0	65%
CpomOR16	Complete	397	*P. excessana* OR16	0	71%
CpomOR21	Incomplete	403	*Ostrinia furnacalis* OR8	7.00E-87	37%
CpomOR26	Complete	397	*C. obliquana* OR26	0	68%
CpomOR29	Complete	398	*Planotortrix octo* OR29	0	78%
CpomOR31	Complete	400	*P. octo* OR5	0	72%
CpomOR32	Complete	417	C. *obliquana* OR32	0	67%
CpomOR38	Incomplete	404	*P. octo* OR38	0	62%
CpomOR41	Complete	389	*P. octo* OR5	2.00E-73	32%
CpomOR44	Complete	438	*P. excessana* OR44	0	83%
CpomOR47	Complete	450	*C. obliquana* OR47	0	79%
CpomOR49	Complete	402	*P. octo* OR4	0	72%
CpomOR60	Complete	398	*C. obliquana* OR58	2.00E-143	51%
CpomOR62	Complete	416	*C. herana* OR63	0	64%
CpomOR67	Complete	457	*C. obliquana* OR67	0	74%
CpomOR68	Complete	401	*C. obliquana* OR68	5.00E-112	45%
CpomOR71	Complete	379	*Bombyx mori* putative odorant receptor 85b-like	2.00E-162	54%
CpomGR1¶	Incomplete	389	*Helicoverpa armigera* GR1	0	91%
CpomGR2¶	Complete	428	*H. armigera* GR2	0	88%
CpomGR3¶	Complete	490	*H. armigera* GR3	0	78%
CpomGR4¶	Complete	406	*H. armigera* GR5	2.00E-151	56%
CpomGR6¶	Incomplete	260	*H. armigera* GR6	7.00E-128	75%
CpomGR9¶	Incomplete	433	*C. pomonella* GR8	2.00E-54	48%
CpomGR10¶	Incomplete	365	*C. pomonella* GR8	6.00E-47	49%
CpomGR29	Incomplete	301	*B. mori* GR30	2.00E-04	25%

CpomGR30¶	Incomplete	350	*B. mori* GR30	2.00E-17	26%
CpomGR55	Incomplete	228	*B. mori* GR57	4.00E-04	23%
CpomGR58	Incomplete	244	*B. mori GR58*	4.00E-04	25%
CpomGR60	Incomplete	217	*Helicoverpa assulta GR12*	2.00E-11	27%
CpomGR61	Incomplete	311	*B. mori GR58*	5.00E-14	24%
CpomGR63	Incomplete	329	*B. mori GR63*	1.00E-58	41%
CpomGR68.1¶	Incomplete	219	*B. mori GR68*	2.00E-51	45%
CpomGR68.2¶	Complete	406	*B. mori GR68*	2.00E-79	37%
CpomGR68.3¶	Complete	407	*B. mori GR68*	6.00E-66	34%
CpomGR68.4¶	Complete	408	*B. mori GR68*	1.00E-71	36%
CpomGR68.5	Incomplete	331	*B. mori GR68*	5.00E-43	35%
CpomIR41a.2	Complete	602	*C. pomonella* IR41a.1	0	72%
CpomIR60a	Complete	653	*Plutella xylostella* Uncharacterized Protein LOC105387407	0	49%
CpomIR64a	Complete	603	*O. furnacalis* IR64a	0	51%
CpomIR75d	Incomplete	319	*Danaus plexipus* Hypothetical Protein KGM_16080	0	61%
CpomIR75p.1	Complete	608	*B. mori* iGluR Delta-1 like	0	48%
CpomIR75p.3	Incomplete	440	*Anopheles gambiae* AGAP005466-PA	7.00E-83	36%

¶ indicates presence of conserved insect GR C terminus motif MYhhhhhQF

Supplementary Data S6 - CpomOR non-annotated fragments

>c90726_g1_i1
QRLFLPFQKKYCLVVKKFILEFNLIYYMHKTDFAKRMYHRVNKICSVVYLVSQWQVVLVIILYNIVPLYNNYAAG
MFSKNKPQNTTFEHAVDLELPLNQYTTYRDYFIVSMYNWYTTYNAAVGICMYDVLIFTIVFHIWGHLNILIYDL
KYFSRYFERESPLHSAQEDISSRLKDIIKHYQTIKEFMSITTEAFSSSLFCYLVFNQLSGSNCSGDIWSGDCNCIPA
VN

>c83602_g1_i1_g3_i1
TDTFFFAFCVYMRMHFRILQRRFQDAVGEREGSQFVEVVKRHQDLIHLADQVEILYSKSXXXXXXXXXXXLICLS
AFNVTSVEDMGVVISFMAFLSMSLTQIFLLCYFGDMLMKSSMEISDAIYNSAWYQADQRTKRNVLLVLTKSR
RPCKLTAARFADVNLTAFTTILS

>c33385_g1_i1
ITPIGVYLYRRIQNQPSDLGYVWHATYPFDKTKSIYHEFVYAFEVFSGTSSVWGMLGSDMMFTTMAR

>c109758_g1_i1
AADGRARMALLVLMQRSQKPLYFTALKFGPITMTTYRSIITNSYSYFTLLYTVYRSD-

>c69714_g1_i1
SLKLFVERAKHPIIITGLNMFPLSLPTYISIMKTAYSCFAMVRILQDRQESAIGT-

>c79332_g2_i1
PGLTPKYETPYYEATFVITCVAAAFSAINQTGYIVLFVTLVAHELGHFYVITEIFNEIFKLSSVKTEKKSSDSRKEIDE
KLMFCIRHHQFLIKYHGKISELYQAIFGAQFLMMIIVLVTTLQTMYYWDFSNTILTGVTGIMPLTIYCFGGELMIS
AAENMSEAIYSCGWEMMEPRQARAVGLILCISQRPLHLTAAGFTTMNRDTFAN

>c86602_g1_i1
DCLFYTCSTSICTQFRLLQNDIETIIPERNFDEIVFREEFKKLAVRHEGIMQFVFS

>c59210_g1_i1
LFWLVEGIQMGKGFIELSYIAPCATICLLSSVKATFFYVHQDVLIEVVKKIKSIHPEVDDKRIIDEKTIEDKDLNDVV
QRNVNESNKFLKKLIKLDLAIC

>c100780_g3_i7
TIFNLIYMKNLSSLFKNANFTDGNFVTIITIWPEVHDQSLLAGVGRVGIYILGWLWMLRITIAYLLVIPVNTSLSH
QYMNLQVYFKSIAGTFDERISQSEKEEKFERALKLGIRLHATTIWCAQQVQRTCGNVFSGHIIVNICVLVQLMS
QFQNSDRTLSQMMPILTTFASMLFSTGVIMLSAGDITVEAENLPTAIFQSGWQNCTNQSSYRIRRLMLISMA
QSQKPVIMKSCGFIELSYQSYLSIVKASYSLFSVLY-

>c98680_g2_i4_i4
TMTLLNTVKYYINKEDFDWGRSDITLQLFHPQFELFFAINGIFFNNRESIIRFIWPVLSTLITLIATAFEMMFIXXX
XXXXXXXXXXXXXXXXXXXXXXXXXXXXXXXXXXXXXXXXXXXXXXXXXXXXXXXXXXXXLVWY
WLIFVCGVASLYIANTIFYLLWQSIFATLDEHTVRPLMFPIWLPKDDPHRTPNYEVFMTFEIILIFIVLCTFGVYVYI
LFHLLLHYYNLMDVIL

>c73220_g1_i1

KRLCFRAGPCNEMSLNTFIGILKTSYSFLTLLKGTK-

>c63154_g2_i1_g3_i1_g1_i1
YWWIKKREILRSVLKKAKESDTSTIQAGLFVDKYDRSLSMIKRILLVFYTVNTINEFTTYLPKRADLNENTFSMTP
CVGIEPLMSSPQREICIALASIQEITILNTTHSFQSMMLLLIAHTSVMYRLLSDEITTFSTLLTDPRNYDFVKGRLP
VIIYXXXXXXXXXXXXXXXXXXXXXXXXXXXXXXXXXXXXXXXXXXXXXXXXXXXXXXXXXXXXXXXXXXXXXXXXX
XXXAVYNSGWELMRIKERRAIVSMLLQSQKEVDLLAADLIPVNMSTFATTCQGIYKFATVFKLQRN

>c12852_g1_i1
QYGSAPRIKKRLTENSFDNLLWLVMVGPSLVGYQITRKKIFGSGILFIYLIAVLVVFT

```
CpomOR2b     1  MILNQVYDVLKKRLDDGYVNSPLDFKYVASLQFVMTTIGSWPYKQFGRNRLAAILSTYNA
CpomOR2a     1  MIIKQVYDVLKKRFDDGYVNTPLDFKYVAQLQFVLTTVGSWPYKQFGRNRLAAILSTYNA
CpomOR2c     1  --MNQVYEVLKKKFDDGYVDSPLDFKYVVQLQFLMNTVGSWPYKQFGRNRVAAILSTYNA

CpomOR2b    61  FLILLGTVLCVLGLIYMRESVVKLSFFDLGHNILCLIFDTMYLQRLLTARTKKYQEVIKD
CpomOR2a    61  FLILVSTNLCVLDLIYIRVNRVKLSFFDLGHNILCLIFTFLYLQRLLTARTSKYQEVIKD
CpomOR2c    59  FLILVGTTVCGLGLIYMRVNIVKLSFFDLGHNILCWLLEILYLQRLITARTNKYQETIKG

CpomOR2b   121  YLLHFNLFYFKGRSPYAAKVHAQIHIISGMFTIYVMWQMFIGLLLFIFMPWFNNYNRGMF
CpomOR2a   121  YLLDFNLFYLKGRSPYAAKVQAQTHIISGMFTIYVMWQMVIGVSLFIFMPWFNNYNRGMF
CpomOR2c   119  YLLDFNLFFFKGRSPYAAKVHAQIHIISGMFTIYVMWQMVIGVSLFMFMPWFNNYSRGMF

CpomOR2b   181  GENRPQNSTFEHSVYYYLPDDIDTTEEEYWLLFIFNMLLSYMTTICMCVFDLLLILIVFQ
CpomOR2a   181  SENRPQNSTFEHSVYYYLPDAVYTTEEGYWILFIFNIPISYVTTIGLCVFDLLLILIVFQ
CpomOR2c   179  GENRPQNSTFEHSVYYYLSPGIYTTEEEYWLLFIFNFSLSYVTTIGMCVFDLLLILIVFQ

CpomOR2b   241  IWGHLRILKHNLQNVPLPENSIMYSVEENNNIRMLLKENILHHNFIIQFVNRCSDAFSEY
CpomOR2a   241  IWGHLRILKHNLQNIPLPENSIMYSVEENNNIRMLLKENILHHNIIIQFVDRCSDAFSEY
CpomOR2c   239  IWGHLRILKHNLENIPLPENSIMYSVEENNNIRMLLKENILHHNLIVKFVDRCSDAFSEY

CpomOR2b   301  LFAFYLFMQFNTCILLLEVTTFTADSLAKYGALTVVMHQQLIQVSILFEMLNTKSEQLTD
CpomOR2a   301  LFAFYLFMQFITCILLLEVTTFTADSLAKYGPLTVVMHQQLIQVSILFEMLNTKSEQLID
CpomOR2c   299  LFAFYMFMQLITCILLLEVTSFTADSLAKYGPLTVVMHQQLIQVSIMFEMLNTKSEQLID

CpomOR2b   361  AVYAIPWEHMDTKNKRTVLFFLHRIQTPVSLKAAKVVPVGVNTMSAVLKTTFSYYMMLKA
CpomOR2a   361  AVYAIPWEHMDTKNRRTVLFFLHRIQTPVSLKAAKVVPVGVNTMSAILKTTFSYYMMLKA
CpomOR2c   359  AAYAIPWEHMDTKNRRTVLFFLHRIQTPVSLKAAKVVPVGVNTMFAVLKTTFSYYMMLKT

CpomOR2b   421  LAGER
CpomOR2a   421  LAGER
CpomOR2c   419  LAGER
```

Supplementary Figure S8. Box-Shade amino acid alignment of CpomOR2 isoforms. Representation of full length ORF for three CpomOR2 receptor types. For each receptor type, at least five clones were sequenced. Presence of white background at each position indicates non-conserved sequence across receptor types. Presence of grey background indicates conserved residue change, and presence of black background indicates identical residues.

```
CpomOR2a      1 ATGATTATAAACAAGTGTATGATGTTTTGAAGAAGAGATTTGATGATGGTTACGTCAACACGCCTCTGGATTTCAAATA
CpomOR2b      1 ATGATTTTAAACCAAGTGTATGATGTTTTGAAGAAGAGACTTGATGATGGTTACGTGAACTCGCCTCTGGATTTCAAGTA
CpomOR2c      1 ------ATGAATCAAGTGTACGAAGTTTTGAAGAAGAAATTTGATGATGGTTACGTCGACTCGCCTCTGGATTTCAAGTA

CpomOR2a     81 TGTGGCTCAACTGCAATTCGTGCTGACGACCGTAGGCTCCTGGCCATATAAACAATTTGGACGAAATCGATTAGCGGCCA
CpomOR2b     81 TGTGGCTTCACTGCAATTCGTGATGACAACCATAGGCTCCTGGCCATATAAGCAATTTGGACGAAATCGTTTAGCGGCCA
CpomOR2c     75 TGTGGTTCAACTTCAATTCTTGATGAACACTGTAGGCTCCTGGCCATATAAGCAATTTGGAAGAAATCGAGTAGCGGCCA

CpomOR2a    161 TTTTGTCTACGTACAACGCCTTTCTAATACTCGTTAGCACCAATTTGTGCGTTTTGGAYTTAATCTATATACGTGTGAAT
CpomOR2b    161 TTTTGTCTACGTACAACGCCTTTCTAATACTCCTTGGCACCGTTTTGTGCGTTTTGGGCTTAATCTATATGCGTGAGAGT
CpomOR2c    155 TTTTGTCTACGTACAACGCCTTTCTAATACTTGTTGGCACCACTGTGTGCGGTTTGGGATTAATCTATATGCGTGTGAAT

CpomOR2a    241 AGAGTAAAACTATCGTTYTTTGATTTGGGRCATAATATATTGTGTTTGATTTTTACTTTCTTGTATCTGCAAAGACTATT
CpomOR2b    241 GTAGTAAAACTGTCATTTTTTGATTTGGGCCATAATATTTTGTGTTTGATTTTCGATACCATGTATCTGCAAAGACTATT
CpomOR2c    235 ATAGTAAAACTGTCGTTTTTTGATTTGGGACATAATATTTTGTGTTGGCTTTTAGAAATCTTGTATCTGCAAAGATTGAT

CpomOR2a    321 GACGGCAAGGACAAGTAAATATCAAGAGGTAATAAAGGATTATTTACTTGACTTTAACTTGTTTTACTTSAAGGGCCGGT
CpomOR2b    321 GACGGCAAGGACAAAGAAATATCAAGAGGTAATAAAGGATTATTTACTTCACTTTAACTTGTTTTACTTCAAGGGCCGGT
CpomOR2c    315 AACAGCAAGGACAAATAAATATCAAGAAACAATAAAGGGTTATTTACTTGACTTCAACTTGTTTTTCTTCAAAGGCCGGT

CpomOR2a    401 CACCATACGCTGCAAAGGTACAGGCGCAAACACACATTATATCAGGAATGTTCACGATCTACGTGATGTGGCAGATGGTC
CpomOR2b    401 CACCATACGCTGCAAAGGTACACGCGCAAATCCACATTATATCAGGAATGTTCACGATCTACGTGATGTGGCAGATGTTC
CpomOR2c    395 CACCATATGCTGCAAAGGTACATGCGCAAATCCACATCATATCAGGAATGTTCACGATCTACGTGATGTGGCAGATGGTC

CpomOR2a    481 ATAGGAGTGTCTCTGTTCATATTCATGCCGTGGTTCAACAATTATAACAGAGGCATGTTCAGTGAAAACCGGCCCCAAAA
CpomOR2b    481 ATAGGATTGTTACTGTTCATATTCATGCCGTGGTTCAACAACTATAACAGAGGCATGTTCGGCGAAAACCGGCCCCAAAA
CpomOR2c    475 ATCGGAGTGTCTCTGTTCATGTTCATGCCGTGGTTCAATAACTATAGCAGAGGCATGTTCGGCGAAAATCGGCCCCAAAA

CpomOR2a    561 CAGCACGTTCGAGCACTCTGTATACTATTATCTGCCTGATGCCGTTTATACTACTGAAGAGGGATACTGGATATTATTCA
CpomOR2b    561 CAGCACGTTCGAGCACTCTGTATACTATTATCTACCTGATGACATTGATACGACTGAAGAGGAATATTGGCTATTATTTA
CpomOR2c    555 CAGCACGTTCGAGCACTCTGTATATTATTATCTATCTCCTGGCATTTATACAACTGAAGAGGAATATTGGCTATTATTCA

CpomOR2a    641 TCTTCAATATTCCAATTTCCTACGTCACCACAATTGGTCTTTGCGTTTTTGATCTCTTACTGATCTTGATAGTGTTCCAA
CpomOR2b    641 TCTTCAATATGTTACTTTCCTATATGACCACAATTTGCATGTGCGTTTTTGATCTCTTACTGATCTTGATAGTGTTCCAA
CpomOR2c    635 TATTCAATTTTTCACTTTCCTATGTAACCACAATTGGCATGTGCGTTTTTGATCTCTTACTGATCTTGATAGTTTTCCAA

CpomOR2a    721 ATTTGGGGGCACCTGAGGATTCTGAAGCATAATTTGCAAAACATTCCACTGCCGGAAAACAGTATTATGTACTCAGTTGA
CpomOR2b    721 ATTTGGGGGCACCTGAGGATTCTGAAGCATAATTTGCAAAACGTTCCACTGCCGGAAAACAGTATTATGTACTCAGTTGA
CpomOR2c    715 ATTTGGGGGCACCTGAGGATTCTGAAGCATAACTTGGAAAATATTCCACTGCCGGAAAACAGTATTATGTACTCAGTTGA

CpomOR2a    801 AGAGAATAATAACATACGAATGTTATTAAAGGAGAATATATTACATCACAACATTATTATACAATTTGTGGACAGATGCT
CpomOR2b    801 AGAGAATAATAACATACGAATGTTATTGAAGGAGAATATATTACATCACAACTTTATTATACAATTCGTGAACAGATGCT
CpomOR2c    795 AGAAAATAATAATATACGAATGTTGTTAAAGGAGAATATATTACATCACAATCTTATTGTAAAATTCGTGGACAGATGCT

CpomOR2a    881 CGGACGCATTCAGCGAATATTTGTTCGCGTTCTACCTGTTCATGCAGTTTATCACCTGCATCTTACTGCTGGAAGTCACT
CpomOR2b    881 CGGACGCATTCAGCGAATATTTGTTTGCGTTCTACTTGTTCATGCAGTTCAACACCTGCATCTTACTGCTGGAAGTCACT
CpomOR2c    875 CGGACGCATTCAGTGAATATTTGTTCGCATTCTACATGTTCATGCAGCTTATCACCTGCATCTTACTACTGGAAGTCACG

CpomOR2a    961 ACGTTCACCGCGGACTCGCTGGCGAAGTACGGCCCCCTAACCGTCGTCATGCATCAGCAGTTAATACAAGTGTCCATATT
CpomOR2b    961 ACGTTCACCGCGGACTCGCTGGCGAAGTACGGCGCCCTAACCGTCGTCATGCATCAGCAGTTAATACAAGTGTCCATATT
CpomOR2c    955 TCGTTCACCGCAGACTCGCTGGCGAAGTACGGCCCCTTAACCGTCGTCATGCATCAGCAGTTAATACAAGTGTCGATAAT

CpomOR2a   1041 GTTCGAGATGTTRAACACCAAGAGTGAGCAGCTGATCGACGCTGTATACGCCATCCCGTGGGAGCATATGGACACGAAGA
CpomOR2b   1041 GTTCGAGATGTTGAACACCAAGAGTGAGCAGCTGACCGACGCTGTATACGCCATCCCGTGGGAGCATATGGACACGAAAA
CpomOR2c   1035 GTTCGAGATGTTGAACACCAAGAGTGAGCAATTAATCGACGCCGCATACGCCATCCCGTGGGAGCATATGGACACGAAGA

CpomOR2a   1121 ACAGGAGAACTGTGTTGTTTTTCCTGCACAGGATTCAGACCCCAGTCAGTCTGAAGGCCGCCAAGGTCGTACCTGTGGGA
CpomOR2b   1121 ACAAGAGAACTGTGTTGTTTTTTCTGCACAGGATTCAGACCCCAGTCAGTCTGAAGGCCGCCAAGGTCGTCCCTGTGGGG
CpomOR2c   1115 ACAGGAGAACTGTGTTGTTTTTTCTGCACAGGATTCAGACCCCAGTCAGTCTGAAGGCCGCTAAGGTCGTCCCTGTGGGG

CpomOR2a   1201 GTCAACACTATGTCTGCAATTCTGAAGACCACGTTCTCGTACTACATGATGCTGAAGGCTTTAGCTGGGGAGCGATAA
CpomOR2b   1201 GTCAACACTATGTCCGCAGTTCTGAAGACCACGTTCTCGTATTACATGATGCTGAAGGCGTTGGCTGGGGAGCGATAA
CpomOR2c   1195 GTCAACACTATGTTTGCGGTTCTGAAGACTACGTTCTCGTATTACATGATGTTGAAGACTTTAGCTGGGGAGCGATAA
```

Supplementary Figure S9. Box-Shade nucleotide alignment of CpomOR2 isoforms. Representation of full length ORF for three CpomOR2 receptor types. For each receptor type, at least five clones were sequenced. Presence of white background at each position indicates non-conserved sequence across receptor types. Presence of grey background indicates synonymous nucleotide change, and presence of black background indicates identical residues. A=Adenine, G=Guanine, C=Cytosine, T=Thymine, R=Purine (A or G), Y=Pyrimidine (C or T).

```
CpomOR2a_12-3     1 ATGATTATAAAACAAGTGTATGATGTTTTGAAGAAGAGATTTGATGATGGTTACGTCAAC
CpomOR2a_13-1     1 ATGATTATAAAACAAGTGTATGATGTTTTGAAGAAGAGATTTGATGATGGTTACGTCAAC
CpomOR2a_3-4      1 ATGATTATAAAACAAGTGTATGATGTTTTGAAGAAGAGATTTGATGATGGTTACGTCAAC
CpomOR2a_13-2     1 ATGATTATAAAACAAGTGTATGATGTTTTGAAGAAGAGATTTGATGATGGTTACGTCAAC
CpomOR2a_2-2      1 ATGATTATAAAACAAGTGTATGATGTTTTGAAGAAGAGATTTGATGATGGTTACGTCAAC
CpomOR2a_12-1     1 ATGATTATAAAACAAGTGTATGATGTTTTGAAGAAGAGATTTGATGATGGTTACGTCAAC
CpomOR2a_2-1      1 ATGATTATAAAACAAGTGTATGATGTTTTGAAGAAGAGATTTGATGATGGTTACGTCAAC

CpomOR2a_12-3    61 ACGCCTCTGGATTTCAAATATGTGGCTCAACTGCAATTCGTGCTGACGACCGTAGGCTCC
CpomOR2a_13-1    61 ACGCCTCTGGATTTCAAATATGTGGCTCAACTGCAATTCGTGCTGACGACCGTAGGCTCC
CpomOR2a_3-4     61 ACGCCTCTGGATTTCAAATATGTGGCTCAACTGCAATTCGTGCTGACGACCGTAGGCTCC
CpomOR2a_13-2    61 ACGCCTCTGGATTTCAAATATGTGGCTCAACTGCAATTCGTGCTGACGACCGTAGGCTCC
CpomOR2a_2-2     61 ACGCCTCTGGATTTCAAATATGTGGCTCAACTGCAATTCGTGCTGACGACCGTAGGCTCC
CpomOR2a_12-1    61 ACGCCTCTGGATTTCAAATATGTGGCTCAACTGCAATTCGTGCTGACGACCGTAGGCTCC
CpomOR2a_2-1     61 ACGCCTCTGGATTTCAAATATGTGGCTCAACTGCAATTCGTGCTGACGACCGTAGGCTCC

CpomOR2a_12-3   121 TGGCCATATAAACAATTTGGACGAAATCGATTAGCGGCCATTTTGTCTATGTACAACGCC
CpomOR2a_13-1   121 TGGCCATATAAACAATTTGGACGAAATCGATTAGCGGCCATTTTGTCTACGTACAACGCC
CpomOR2a_3-4    121 TGGCCATATAAACAATTTGGACGAAATCGATTAGCGGCCATTTTGTCTACGTACAACGCC
CpomOR2a_13-2   121 TGGCCATATAAACAATTTGGACGAAATCGATTAGCGGCCATTTTGTCTACGTACAACGCC
CpomOR2a_2-2    121 TGGCCATATAAACAATTTGGACGAAATCGATTAGCGGCCATTTTGTCTACGTACAACGCC
CpomOR2a_12-1   121 TGGCCATATAAACAATTTGGACGAAATCGATTAGCGGCCATTTTGTCTACGTACAACGCC
CpomOR2a_2-1    121 TGGCCATATAAACAATTTGGACGAAATCGATTAGCGGCCACTTTGTCTACGTACAACGCC

CpomOR2a_12-3   181 TTTCTAATACTCGTTAGCGCCAATTTGTGCGTTTTGGATTTAATCTATATACGCGTGAAT
CpomOR2a_13-1   181 TTTCTAATACTCGTTAGCACCAATTTGTGCGTTTTGGATTTAATCTATATACGCGTGAAT
CpomOR2a_3-4    181 TTTCTAATACTCGTTAGCACCAATTTGTGCGTTTTGGACTTAATCTATATACGTGTGAAT
CpomOR2a_13-2   181 TTTCTAATACTCGTTAGCACCAATTTGTGCGTTTTGGACTTAATCTATATACGTGTGAAT
CpomOR2a_2-2    181 TTTCTAATACTCGTTAGCACCAATTTGTGCGTTTTGGACTTAATCTATATACGTGTGAAT
CpomOR2a_12-1   181 TTTCTAATACTCGTTAGCACCAATTTGTGCGTTTTGGACTTAATCTATATACGTGTGAAT
CpomOR2a_2-1    181 TTTCTAATACTCGTTAGCACCAATTTGTGCGTTTTGGATTTAATCTATATACGTGTGAAT

CpomOR2a_12-3   241 AGAGTAAAACTATCGTTTTTTGATTTGGGACATAATATATTGTGTTTGATTTTTACTTTC
CpomOR2a_13-1   241 AGAGTAAAACTATCGTTTTTTGATTTGGGACATAATATATTGTGTTTGATTTTTACTTTC
CpomOR2a_3-4    241 AGAGTAAAACTATCGTTCTTTGATTTGGGGCATAATATATTGTGTTTGATTTTTACTTTC
CpomOR2a_13-2   241 AGAGTAAAACTATCGTTCTTTGATTTGGGGCATAATATATTGTGTTTGATTTTTACTTTC
CpomOR2a_2-2    241 AGAGTAAAACTATCGTTCTTTGATTTGGGGCATAATATATTGTGTTTGATTTTTACTTTC
CpomOR2a_12-1   241 AGAGTAAAACTATCGTTCTTTGATTTGGGGCATAATATATTGTGTTTGATTTTTACTTTC
CpomOR2a_2-1    241 AGAGTAAAACTATCGTTTTTTGATTTGGGACATAATATATTGTGTTTGATTTTTACTTTC

CpomOR2a_12-3   301 TTGTATCTGCAAAGACTATTGACGGCAAGGACAAGTAAATATCAAGAGGTAATAAAGGAT
CpomOR2a_13-1   301 TTGTATCTGCAAAGACTATTGACGGCAAGGACAAGTAAATATCAAGAGGTAATAAAGGAT
CpomOR2a_3-4    301 TTGTATCTGCAAAGACTATTGACGGCAAGGACAAGTAAATATCAAGAGGTAATAAAGGAT
CpomOR2a_13-2   301 TTGTATCTGCAAAGACTATTGACGGCAAGGACAAGTAAATATCAAGAGGTAATAAAGGAT
CpomOR2a_2-2    301 TTGTATCTGCAAAGACTATTGACGGCAAGGACAAGTAAATATCAAGAGGTAATAAAGGAT
CpomOR2a_12-1   301 TTGTATCTGCAAAGACTATTGACGGCAAGGACAAGTAAATATCAAGAGGTAATAAAGGAT
CpomOR2a_2-1    301 TTGTATCTGCAAAGACTATTGACGGCAAGGACAAGTAAATATCAAGAGGTAATAAAGGAT

CpomOR2a_12-3   361 TATTTACTTGACTTTAACTTGTTTTACTTCAAGGGCCGGTCACCATACGCTGCAAAGGTA
CpomOR2a_13-1   361 TATTTACTTGACTTTAACTTGTTTTACTTCAAGGGCCGGTCACCATACGCTGCAAAGGTA
CpomOR2a_3-4    361 TATTTACTTCACTTTAACTTGTTTTACTTGAAGGGCCGGTCACCATACGCTGCAAAGGTA
CpomOR2a_13-2   361 TATTTACTTCACTTTAACTTGTTTTACTTGAAGGGCCGGTCACCATACGCTGCAAAGGTA
CpomOR2a_2-2    361 TATTTACTTCACTTTAACTTGTTTTACTTGAAGGGCCGGTCACCATACGCTGCAAAGGTA
CpomOR2a_12-1   361 TATTTACTTGACTTTAACTTGTTTTACTTCAAGGGCCGGTCACCATACGCTGCAAAGGTA
CpomOR2a_2-1    361 TATTTACTTGACTTTAACTTGTTTTACTTGAAGGGCCGGTCACCATACGCTGCAAAGGTA

CpomOR2a_12-3   421 CAGGCGCAAACACACATTATATCAGGAATGTTCACGATCTACGTGATGTGGCAGATGGTC
CpomOR2a_13-1   421 CAGGCGCAAACACACATTATATCAGGAATGTTCACGATCTACGTGATGTGGCAGATGGTC
CpomOR2a_3-4    421 CAGGCGCAAACACACATTATATCAGGAATGTTCACGATCTACGTGATGTGGCAGATGTTC
CpomOR2a_13-2   421 CAGGCGCAAACACACATTATATCAGGAATGTTCACGATCTACGTGATGTGGCAGATGTTC
CpomOR2a_2-2    421 CAGGCGCAAACACACATTATATCAGGAATGTTCACGATCTACGTGATGTGGCAGATGTTC
CpomOR2a_12-1   421 CAGGCGCAAACACATATTATATCAGGAATGTTCACGATCTACGTGATGTGGCAGATGGTC
CpomOR2a_2-1    421 CAGGCGCAAACACACATTATATCAGGAATGTTCACGATCTACGTGATGTGGCAGATGGTC
```

```
CpomOR2a_12-3   481  ATAGGAGTATCTCTGTTCATATTCATGCCGTGGTTCAACAATTATAACAGAGGCATGTTC
CpomOR2a_13-1   481  ATAGGAGTATCTCTGTTCATATTCATGCCGTGGTTCAACAATTATAACAGAGGCATGTTC
CpomOR2a_3-4    481  ATAGGAGTGTCTCTGTTCATATTCATGCCGTGGTTCAACAATTATAACAGAGGCATGTTC
CpomOR2a_13-2   481  ATAGGAGTGTCTCTGTTCATATTCATGCCGTGGTTCAACAATTATAACAGAGGCATGTTC
CpomOR2a_2-2    481  ATAGGAGTGTCTCTGTTCATATTCATGCCGTGGTTCAACAATTATAACAGAGGCATGTTC
CpomOR2a_12-1   481  ATAGGAGTGTCTCTGTTCATATTCATGCCGTGCTTCAACAATTATAACAGAGGCATGTTC
CpomOR2a_2-1    481  ATAGGAGTATCTCTGTTCATATTCATGCCGTGGTTCAACAATTATAACAGAGGCATGTTC

CpomOR2a_12-3   541  AGTGAAAACCGGCCCCAAAACAGCACGTTCGAGCACTCTGTATACTATTATCTGCCTGAT
CpomOR2a_13-1   541  AGTGAAAACCGGCCCCAAAACAGCACGTTCGAGCACTCTGTATACTATTATCTGCCTGAT
CpomOR2a_3-4    541  AGTGAAAACCGGCCCCAAAACAGCACGTTCGAGCACTCTGTATACTATTATCTGCCTGAT
CpomOR2a_13-2   541  AGTGAAAACCGGCCCCAAAACAGCACGTTCGAGCACTCTGTATACTATTATCTGCCTGAT
CpomOR2a_2-2    541  AGTGAAAACCGGCCCCAAAACAGCACGTTCGAGCACTCTGTATACTATTATCTGCCTGAT
CpomOR2a_12-1   541  AGTGAAAACCGGCCCCAAAACAGCACGTTCGAGCACTCTGTATACTATTATCTGCCTGAT
CpomOR2a_2-1    541  AGTGAAAACCGGCCCCAAAACAGCACGTTCGAGCACTCTGTATACTATTATCTGCCTGAT

CpomOR2a_12-3   601  GCCGTTTATACTACTGAAGAGGGATACTGGATATTATTCATCTTCAATATTCCAATTTCC
CpomOR2a_13-1   601  GCCGTTTATACTACTGAAGAGGGATACTGGATATTATTCATCTTCAATATTCCAATTTCC
CpomOR2a_3-4    601  GCCGTTTATACTACTGAAGAGGGATACTGGATATTATTCATCTTCAATATTCCAATTTCC
CpomOR2a_13-2   601  GCCGTTTATACTACTGAAGAGGGATACTGGATATTATTCATCTTCAATATTCCAATTTCC
CpomOR2a_2-2    601  GCCGTTTATACTACTGAAGAGGGATACTGGATATTATTCATCTTCAATATTCCAATTTCC
CpomOR2a_12-1   601  GCCGTTTATACTACTGAAGAGGGATACTGGATATTATTCATCTTCAATATTCCAATTTCC
CpomOR2a_2-1    601  GCCGTTTATACTACTGAAGAGGGATACTGGATATTATTCGTCTTCAATATTCCAATTTCC

CpomOR2a_12-3   661  TACGTCACCACAATTGGTCTTTGCGTTTTTGATCTCTTACTGATCTTGATAGTGTTCCAA
CpomOR2a_13-1   661  TACGTCACCACAATTGGTCTTTGCGTTTTTGATCTCTTACTGATCTTGATAGTGTTCCAA
CpomOR2a_3-4    661  TACGTCACCACAATTGGTCTTTGCGTTTTTGATCTCTTACTGATCTTGATAGTGTTCCAA
CpomOR2a_13-2   661  TACGTCACCACAATTGGTCTTTGCGTTTTTGATCTCTTACTGATCTTGATAGTGTTCCAA
CpomOR2a_2-2    661  TACGTCACCACAATTGGTCTTTGCGTTTTTGATCTCTTACTGATCTTGATAGTGTTCCAA
CpomOR2a_12-1   661  TACGTCACCACAATTGGTCTTTGCGTTTTTGATCTCTTACTGATCTTGATAGTGTTCCAA
CpomOR2a_2-1    661  TACGTCACCACAATTGGTCTTTGCGTTTTTGATCTCTTACTGATCTTGATAGTGTTCCAA

CpomOR2a_12-3   721  ATTTGGGGGCACCTGAGGATTCTGAAGCATAATTTGCAAAACATTCCACTGCCGGAAAAC
CpomOR2a_13-1   721  ATTTGGGGGCACCTGAGGATTCTGAAGCATAATTTGCAAAACATTCCACTGCCGGAAAAC
CpomOR2a_3-4    721  ATTTGGGGGCACCTGAGGATTCTGAAGCATAATTTGCAAAACATTCCACTGCCGGAAAAC
CpomOR2a_13-2   721  ATTTGGGGGCACCTGAGGATTCTGAAGCATAATTTGCAAAACATTCCACTGCCGGAAAAC
CpomOR2a_2-2    721  ATTTGGGGGCACCTGAGGATTCTGAAGCATAATTTGCAAAACATTCCACTGCCGGAAAAC
CpomOR2a_12-1   721  ATTTGGGGGCACCTGAGGATTCTGAAGCATAATTTGCAAAACATTCCACTGCCGGAAAAC
CpomOR2a_2-1    721  ATTTGGGGGCACCTGAGGATTCTGAAGCATAATTTGCAAAACATTCCACTGCCGGAAAAC

CpomOR2a_12-3   781  AGTATTATTTACTCAGTTGAAGAGAATAATAACATACGAATGTTATTAAAGGAGAATATA
CpomOR2a_13-1   781  AGTATTATTTACTCAGTTGAAGAGAATAATAACATACGAATGTTATTAAAGGAGAATATA
CpomOR2a_3-4    781  AGTATTATGTACTCAGTTGAAGAAAATAATAACATACGAATGTTATTAAAGGAGAATATA
CpomOR2a_13-2   781  AGTATTATGTACTCAGTTGAAGAAAATAATAACATACGAATGTTATTAAAGGAGAATATA
CpomOR2a_2-2    781  AGTATTATGTACTCAGTTGAAGAAAATAATAACATACGAATGTTATTAAAGGAGAATATA
CpomOR2a_12-1   781  AGTATTATGTACTCAGTTGAAGAGAATAATAACATACGAATGTTATTAAAGGAGAATATA
CpomOR2a_2-1    781  AGTATTATGTACTCAGTTGAAGAGAATAATAACATACGAATGTTATTAAAGGAGAATATA

CpomOR2a_12-3   841  TTACATCACAACATTATTATACAATTTGTGGACAGATGCTCGGACGCATTCAGCGAATAT
CpomOR2a_13-1   841  TTACATCACAACATTATTATACAATTTGTGGACAGATGCTCGGACGCATTCAGCGAATAT
CpomOR2a_3-4    841  TTACATCACAACATTATTATACAATTTGTGGACAGATGCTCGGACGCATTCAGCGAATAT
CpomOR2a_13-2   841  TTACATCACAACATTATTATACAATTTGTGGACAGATGCTCGGACGCATTCAGCGAATAT
CpomOR2a_2-2    841  TTACATCACAACATTATTATACAATTTGTGGACAGATGCTCGGACGCATTCAGCGAATAT
CpomOR2a_12-1   841  TTACATCACAACATTATTATACAATTCGTGGACAGATGCTCCGACGCATTCAGTGAATAT
CpomOR2a_2-1    841  TTACATCACAACATTATTATACAATTCGTGGACAGATGCTCGGACGCATTCAGCGAATAT

CpomOR2a_12-3   901  TTGTTCGCGTTCTACCTGTTCATGCAGTTTATCACCTGCATCTTACTGCTGGAAGTCACT
CpomOR2a_13-1   901  TTGTTCGCGTTCTACCTGTTCATGCAGTTTATCACCTGCATCTTACTGCTGGAAGTCACT
CpomOR2a_3-4    901  TTGTTCGCGTTCTACCTGTTCATGCAGTTTATCACCTGCATCTTACTGCTGGAAGTCACT
CpomOR2a_13-2   901  TTGTTCGCGTTCTACCTGTTCATGCAGTTTATCACCTGCATCTTACTGCTGGAAGTCACT
CpomOR2a_2-2    901  TTGTTCGCGTTCTACCTGTTCATGCAGTTTATCACCTGCATCTTACTGCTGGAAGTCACT
CpomOR2a_12-1   901  TTGTTCGCGTTCTACCTGTTCATGCAGTTTATCACCTGCATCTTACTGCTGGAAGTCACT
CpomOR2a_2-1    901  TTGTTCGCGTTCTACCTGTTCATGCAGTTTATCACCTGCATCTTACTGCTGGAAGTCACT
```

```
CpomOR2a_12-3   961  ACGTTCACCGCGGACTCGCTAGCGAAGTACGGCCCCCTAACCGTCGTCATGCATCAGCAG
CpomOR2a_13-1   961  ACGTTCACCGCGGACTCGCTAGCGAAGTACGGCCCCCTAACCGTCGTCATGCATCAGCAG
CpomOR2a_3-4    961  ACGTTCACCGCGGACTCGCTGGCGAAGTACGGCCCCCTAACCGTCGTCATGCATCAGCAG
CpomOR2a_13-2   961  ACGTTCACCGCGGACTCGCTGGCGAAGTACGGCCCCCTAACCGTCGTCATGCATCAGCAG
CpomOR2a_2-2    961  ACGTTCACCGCGGACTCGCTGGCGAAGTACGGCCCCCTAACCGTCGCCATGCATCAGCAG
CpomOR2a_12-1   961  ACGTTCACCGCGGACTCGCTGGCGAAGTACGGCCCCCTAACCGTCGTCATGCATCAGCAG
CpomOR2a_2-1    961  ACGTTCACCGCGAACTCGCTAGCGAAGTACGGCCCCCTAACCGTCGTCATGCATCAGCAG

CpomOR2a_12-3  1021  TTAATACAAGTGTCCATATTGTTCGAGATGCTGAACACCAAGAGTGAGCAGCTGATCGAC
CpomOR2a_13-1  1021  TTAATACAAGTGTCCATATTGTTCGAGATGCTGAACACCAAGAGTGAGCAGCTGATCGAC
CpomOR2a_3-4   1021  TTAATACAAGTGTCCATATTGTTCGAGATGTTAAACACCAAGAGTGAGCAGCTGATCGAC
CpomOR2a_13-2  1021  TTAATACAAGTGTCCATATTGTTCGAGATGTTAAACACCAAGAGTGAGCAGCTGATCGAC
CpomOR2a_2-2   1021  TTAATACAAGTGTCCATATTGTTCGAGATGTTAAACACCAAGAGTGAGCAGCTGATCGAC
CpomOR2a_12-1  1021  TTAATACAAGTGTCCATATTGTTCGAGATGTTGAACACCAAGAGTGAGCAGCTGATCGAC
CpomOR2a_2-1   1021  TTAATACAAGTGTCCATATTGTTCGAGATGCTGAACACCAAGAGTGAGCAGCTGATCGAC

CpomOR2a_12-3  1081  ACCGTATACGCCATCCCGTGGGAGCATATGGACACGAAGAACAGGAGAACTGTGTTGTTT
CpomOR2a_13-1  1081  ACCGTATACGCCATCCCGTGGGAGCATATGGACACGAAGAACAGGAGAACTGTGTTGTTT
CpomOR2a_3-4   1081  GCTGTATACGCCATCCCGTGGGAGCATATGGACACGAAGAACAGGAGAACTGTGTTGTTT
CpomOR2a_13-2  1081  GCTGTATACGCCATCCCGTGGGAGCATATGGACACGAAGAACAGGAGAACTGTGTTGTTT
CpomOR2a_2-2   1081  GCTGTATACGCCATCCCGTGGGAGCATATGGACACGAAGAACAGGAGAACTGTGTTGTTT
CpomOR2a_12-1  1081  GCTGTATACGCCATCCCGTGGGAGCATATGGACACGAAGAACAGGAGAACTGTGTTGTTT
CpomOR2a_2-1   1081  GCTGTATACGCCATCCCGTGGGAGCATATGGACACGAAGAACAGGAGAACTGTGTTGTTT

CpomOR2a_12-3  1141  TTCCTGCACAGGATTCAGACCCCAGTCAGTCTGAAGGCTGCCAAGGTCGTACCTGTGGGA
CpomOR2a_13-1  1141  TTCCTGCACAGGATTCAGACCCCAGTCAGTCTGAAGGCTGCCAAGGTCGTACCTGTGGGA
CpomOR2a_3-4   1141  TTCCTGCACAGGATTCAGACCCCAGTCAGTCTGAAGGCCGCCAAGGTCGTACCTGTGGGA
CpomOR2a_13-2  1141  TTCCTGCACAGGATTCAGACCCCAGTCAGTCTGAAGGCCGCCAAGGTCGTACCTGTGGGA
CpomOR2a_2-2   1141  TTCCTGCACAGGATTCAGACCCCAGTCAGTCTGAAGGCCGCCAAGGTCGTACCTGTGGGA
CpomOR2a_12-1  1141  TTCCTGCACAGGATTCAGACCCCAGTCAGTCTGAAGGCCGCCAAGGTCGTACCTGTGGGA
CpomOR2a_2-1   1141  TTCCTGCACAGGATTCAGACCCCAGTCAGTCTGAAGGCCGCCAAGGTCGTACCTGTGGGA

CpomOR2a_12-3  1201  GTCAACACTATGTCTGCAATTCTGAAGACCACGTTCTCGTACTACATGATGCTGAAGGCT
CpomOR2a_13-1  1201  GTCAACACTATGTCTGCAATTCTGAAGACCACGTTCTCGTACTACATGATGCTGAAGGCT
CpomOR2a_3-4   1201  GTCAACACTATGTCTGCAATTCTGAAGACCACGTTCTCGTACTACATGATGCTGAAGGCT
CpomOR2a_13-2  1201  GTCAACACTATGTCTGCAATTCTGAAGACCACGTTCTCGTACTACATGATGCTGAAGGCT
CpomOR2a_2-2   1201  GTCAACACTATGTCTGCAATTCTGAAGACCACGTTCTCGTACTACATGATGCTGAAGGCT
CpomOR2a_12-1  1201  GTCAACACTATGTCTGCAATACTGAAGACCACGTTCTCGTACTACATGATGCTGAAGGCT
CpomOR2a_2-1   1201  GTCAACACTATGTCTGCAATTCTGAAGACCACGTTCTCGTACTACATGATGTTGAAGGCT

CpomOR2a_12-3  1261  TTAGCTGGGGAGCGATAA
CpomOR2a_13-1  1261  TTAGCTGGGGAGCGATAA
CpomOR2a_3-4   1261  TTAGCTGGGGAGCGATAA
CpomOR2a_13-2  1261  TTAGCTGGGGAGCGATAA
CpomOR2a_2-2   1261  TTAGCTGGGGAGCGATAA
CpomOR2a_12-1  1261  TTAGCTGGGGAGCGATAA
CpomOR2a_2-1   1261  TTAGCTGGGGAGCGATAA
```

Supplementary Figure S10. Box-Shade nucleotide alignment of CpomOR2a variants. Full length ORF for seven cloned variants of CpomOR2a receptor subtypes. Presence of white background at each position indicates non-conserved sequence across receptor types. Presence of grey background indicates synonymous nucleotide change, and presence of black background indicates identical residues. Distinct SNPs found in multiple clones are indicated with red text.

```
CpomOR2a_12-3     1  MIIKQVYDVLKKRFDDGYVNTPLDFKYVAQLQFVLTTVGSWPYKQFGRNRLAAILSMYNA
CpomOR2a_13-1     1  MIIKQVYDVLKKRFDDGYVNTPLDFKYVAQLQFVLTTVGSWPYKQFGRNRLAAILSTYNA
CpomOR2a_2-2      1  MIIKQVYDVLKKRFDDGYVNTPLDFKYVAQLQFVLTTVGSWPYKQFGRNRLAAILSTYNA
CpomOR2a_13-2     1  MIIKQVYDVLKKRFDDGYVNTPLDFKYVAQLQFVLTTVGSWPYKQFGRNRLAAILSTYNA
CpomOR2a_3-4      1  MIIKQVYDVLKKRFDDGYVNTPLDFKYVAQLQFVLTTVGSWPYKQFGRNRLAAILSTYNA
CpomOR2a_2-1      1  MIIKQVYDVLKKRFDDGYVNTPLDFKYVAQLQFVLTTVGSWPYKQFGRNRLAATLSTYNA
CpomOR2a_12-1     1  MIIKQVYDVLKKRFDDGYVNTPLDFKYVAQLQFVLTTVGSWPYKQFGRNRLAAILSTYNA

CpomOR2a_12-3    61  FLILVSANLCVLDLIYIRVNRVKLSFFDLGHNILCLIFTFLYLQRLLTARTSKYQEVIKD
CpomOR2a_13-1    61  FLILVSTNLCVLDLIYIRVNRVKLSFFDLGHNILCLIFTFLYLQRLLTARTSKYQEVIKD
CpomOR2a_2-2     61  FLILVSTNLCVLDLIYIRVNRVKLSFFDLGHNILCLIFTFLYLQRLLTARTSKYQEVIKD
CpomOR2a_13-2    61  FLILVSTNLCVLDLIYIRVNRVKLSFFDLGHNILCLIFTFLYLQRLLTARTSKYQEVIKD
CpomOR2a_3-4     61  FLILVSTNLCVLDLIYIRVNRVKLSFFDLGHNILCLIFTFLYLQRLLTARTSKYQEVIKD
CpomOR2a_2-1     61  FLILVSTNLCVLDLIYIRVNRVKLSFFDLGHNILCLIFTFLYLQRLLTARTSKYQEVIKD
CpomOR2a_12-1    61  FLILVSTNLCVLDLIYIRVNRVKLSFFDLGHNILCLIFTFLYLQRLLTARTSKYQEVIKD

CpomOR2a_12-3   121  YLLDFNLFYFKGRSPYAAKVQAQTHIISGMFTIYVMWQMVIGVSLFIFMPWFNNYNRGMF
CpomOR2a_13-1   121  YLLDFNLFYFKGRSPYAAKVQAQTHIISGMFTIYVMWQMVIGVSLFIFMPWFNNYNRGMF
CpomOR2a_2-2    121  YLLHFNLFYLKGRSPYAAKVQAQTHIISGMFTIYVMWQMFIGVSLFIFMPWFNNYNRGMF
CpomOR2a_13-2   121  YLLHFNLFYLKGRSPYAAKVQAQTHIISGMFTIYVMWQMFIGVSLFIFMPWFNNYNRGMF
CpomOR2a_3-4    121  YLLHFNLFYLKGRSPYAAKVQAQTHIISGMFTIYVMWQMFIGVSLFIFMPWFNNYNRGMF
CpomOR2a_2-1    121  YLLDFNLFYLKGRSPYAAKVQAQTHIISGMFTIYVMWQMVIGVSLFIFMPWFNNYNRGMF
CpomOR2a_12-1   121  YLLDFNLFYFKGRSPYAAKVQAQTHIISGMFTIYVMWQMVIGVSLFIFMPCFNNYNRGMF

CpomOR2a_12-3   181  SENRPQNSTFEHSVYYYLPDAVYTTEEGYWILFIFNIPISYVTTIGLCVFDLLLILIVFQ
CpomOR2a_13-1   181  SENRPQNSTFEHSVYYYLPDAVYTTEEGYWILFIFNIPISYVTTIGLCVFDLLLILIVFQ
CpomOR2a_2-2    181  SENRPQNSTFEHSVYYYLPDAVYTTEEGYWILFIFNIPISYVTTIGLCVFDLLLILIVFQ
CpomOR2a_13-2   181  SENRPQNSTFEHSVYYYLPDAVYTTEEGYWILFIFNIPISYVTTIGLCVFDLLLILIVFQ
CpomOR2a_3-4    181  SENRPQNSTFEHSVYYYLPDAVYTTEEGYWILFIFNIPISYVTTIGLCVFDLLLILIVFQ
CpomOR2a_2-1    181  SENRPQNSTFEHSVYYYLPDAVYTTEEGYWILFVFNIPISYVTTIGLCVFDLLLILIVFQ
CpomOR2a_12-1   181  SENRPQNSTFEHSVYYYLPDAVYTTEEGYWILFIFNIPISYVTTIGLCVFDLLLILIVFQ

CpomOR2a_12-3   241  IWGHLRILKHNLQNIPLPENSIIYSVEENNNIRMLLKENILHHNIIIQFVDRCSDAFSEY
CpomOR2a_13-1   241  IWGHLRILKHNLQNIPLPENSIIYSVEENNNIRMLLKENILHHNIIIQFVDRCSDAFSEY
CpomOR2a_2-2    241  IWGHLRILKHNLQNIPLPENSIMYSVEENNNIRMLLKENILHHNIIIQFVDRCSDAFSEY
CpomOR2a_13-2   241  IWGHLRILKHNLQNIPLPENSIMYSVEENNNIRMLLKENILHHNIIIQFVDRCSDAFSEY
CpomOR2a_3-4    241  IWGHLRILKHNLQNIPLPENSIMYSVEENNNIRMLLKENILHHNIIIQFVDRCSDAFSEY
CpomOR2a_2-1    241  IWGHLRILKHNLQNIPLPENSIMYSVEENNNIRMLLKENILHHNIIIQFVDRCSDAFSEY
CpomOR2a_12-1   241  IWGHLRILKHNLQNIPLPENSIMYSVEENNNIRMLLKENILHHNIIIQFVDRCSDAFSEY

CpomOR2a_12-3   301  LFAFYLFMQFITCILLLEVTTFTADSLAKYGPLTVVMHQQLIQVSILFEMLNTKSEQLID
CpomOR2a_13-1   301  LFAFYLFMQFITCILLLEVTTFTADSLAKYGPLTVVMHQQLIQVSILFEMLNTKSEQLID
CpomOR2a_2-2    301  LFAFYLFMQFITCILLLEVTTFTADSLAKYGPLTVAMHQQLIQVSILFEMLNTKSEQLID
CpomOR2a_13-2   301  LFAFYLFMQFITCILLLEVTTFTADSLAKYGPLTVVMHQQLIQVSILFEMLNTKSEQLID
CpomOR2a_3-4    301  LFAFYLFMQFITCILLLEVTTFTADSLAKYGPLTVVMHQQLIQVSILFEMLNTKSEQLID
CpomOR2a_2-1    301  LFAFYLFMQFITCILLLEVTTFTANSLAKYGPLTVVMHQQLIQVSILFEMLNTKSEQLID
CpomOR2a_12-1   301  LFAFYLFMQFITCILLLEVTTFTADSLAKYGPLTVVMHQQLIQVSILFEMLNTKSEQLID

CpomOR2a_12-3   361  TVYAIPWEHMDTKNRRTVLFFLHRIQTPVSLKAAKVVPVGVNTMSAILKTTFSYYMMLKA
CpomOR2a_13-1   361  TVYAIPWEHMDTKNRRTVLFFLHRIQTPVSLKAAKVVPVGVNTMSAILKTTFSYYMMLKA
CpomOR2a_2-2    361  AVYAIPWEHMDTKNRRTVLFFLHRIQTPVSLKAAKVVPVGVNTMSAILKTTFSYYMMLKA
CpomOR2a_13-2   361  AVYAIPWEHMDTKNRRTVLFFLHRIQTPVSLKAAKVVPVGVNTMSAILKTTFSYYMMLKA
CpomOR2a_3-4    361  AVYAIPWEHMDTKNRRTVLFFLHRIQTPVSLKAAKVVPVGVNTMSAILKTTFSYYMMLKA
CpomOR2a_2-1    361  AVYAIPWEHMDTKNRRTVLFFLHRIQTPVSLKAAKVVPVGVNTMSAILKTTFSYYMMLKA
CpomOR2a_12-1   361  AVYAIPWEHMDTKNRRTVLFFLHRIQTPVSLKAAKVVPVGVNTMSAILKTTFSYYMMLKA

CpomOR2a_12-3   421  LAGER
CpomOR2a_13-1   421  LAGER
CpomOR2a_2-2    421  LAGER
CpomOR2a_13-2   421  LAGER
CpomOR2a_3-4    421  LAGER
CpomOR2a_2-1    421  LAGER
CpomOR2a_12-1   421  LAGER
```

Supplementary Figure S11. Box-Shade amino acid alignment of CpomOR2a variants. Full length ORF for seven cloned variants of CpomOR2a receptor subtypes. Presence of white background at each position indicates non-conserved sequence across receptor types. Presence of grey background indicates conserved residue change, and presence of black background indicates identical residues. Distinct SNPs found in multiple clones are indicated with red text.

a

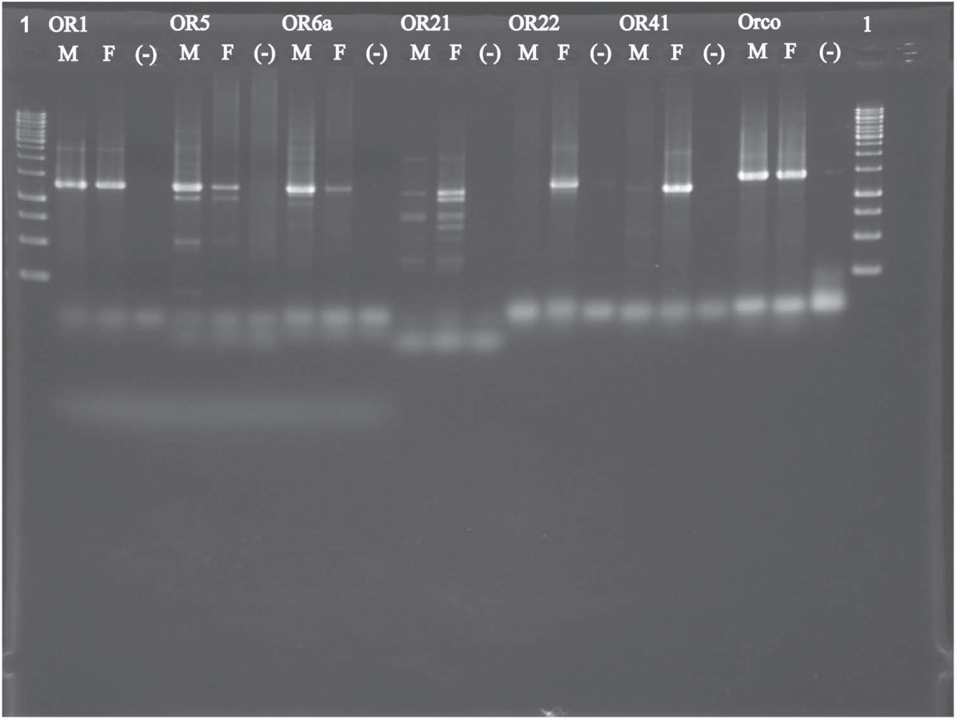

b

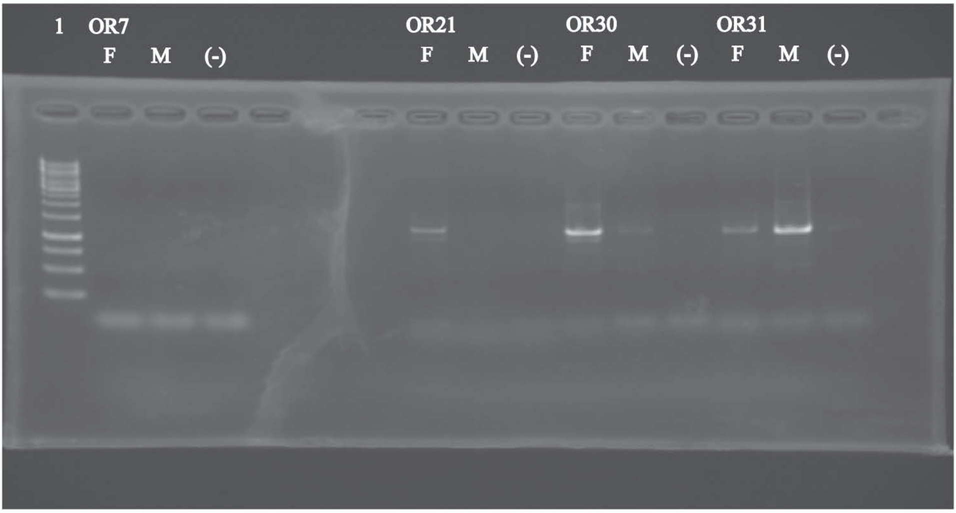

Supplementary Figure S13. Raw gel images showing sex biased OR expression patterns. Representative PCR amplification of full length ORFs for ORs that displayed sex biased FPKM expression values. “1” indicates that the GeneRuler 1kb DNA Ladder was used, with 1kbp and 1.5 kbp bands running in the fourth and fifth positions,from bottom, respectively for both gels. For each assay, “M” indicates male antennae, “F” indicates female antennae and “(-)” indicates “no template control”. For some assays, multiple amplification bands were observed, however, in all cases (excluding OR7), specific amplification of a band of predicted size was observed (between the 1.0 and 1.5 kbp ladder bands); these bands served as source image for Figure 3. Gels in a) and b) contain PCR assay products that were amplified and run on different days, but for all assays, cDNA input and thermocycling amplification conditions were the same. OR21 technical replicates were performed for both gels, and Orco is included as a positive control.

Supplementary Data S14 - CpomGR Protein Fasta Sequences

>CpomGR1
MGVMPIMRVPRDAQTTKRTTYNWISKATFWAYLVWSLESIIVVKVGRERYENFQKSSNKRFDEVIYNIIFLSILI
PHFLLPIASWRHGPQVAIFKNMWTHYQLKYLKITGTPIVFPNLYSLTWGLCFFSWGLSFAVILSQHYLQDDFEL
WHSLAYYHIIAMLDGFCSLWYINCNAFGTASKGLAQNLHKALEADHPALMLAQYRHLWVDLSHMMQQLGR
AYSNMYGIYCMVIFFTTTISLYGALSEILEHGLSYKEMGLFVIVGYCMTLLFIICNEAYHASRKVGHEFQVRLLNV
NLGAIDHSTQREVEMFLVAIAKNPPIMNLDGFTNINRELFTANISFMSTYLIVLMQFKLTLLRQGARKAVRAIVK
AIFNTTTMLPDEEYEDEE*

>CpomGR2
MIPDHYFDEGLNGSLYPDDMKQLNTVKLVYEKTQADYEQEQRDMLSSQDGDTCETHDQFYRDHKLLLVLFR
ALAVMPITRSRPGTITFSWKSRATMYAISFYIVATVVVLMVGYERVMILRSIKKFDEYIYAVLFVAFLVPHFWIPF
VGWGVAHQVAIYKTSWGKFQVRYYRVTGENLQFPNLQTQIVIISVGCLLLAVCFLLSLCALMDGFLLRHTTAYY
HIIIMINMNCALWFINCKGIKIASQSLSECFRRDVNVEISAKLISRYRFLWLNLSELLQSLGNAYARTYSTYCLFMF
FNITIAVYGALSEIVDHGFGFSFKEMGLFVDAAYCSTLLFIFADCSHKSTLKVAAGVQDTLLGIDVLAIDRPAQKEI
DHFIQAIEMNPAVVSLKGYAHVNRELLTSAISMIAIYLIVLLQFKISLPKADS*

>CpomGR3
MAFYTNNSLFPNQPPIPNGIAAQMDEKSKNKIIFLDVTPNRTPRLPTPNNAIAPIQDNLINPDITRDIIYENIKP
VFTLLKIMGVLPLSRPVPGVTQFQPTSPSMLYSVVVYCSLIGYLLYLSLNKVQIVRTGAQEGKFEEAVIEYLFTVYL
FPMIAVPILWYETRKIAEVLNGWVEYEIAYKKLSNRVLPVGLYKKALAMSIVIPALSTASVIITHVTMVHFKLLQII
PYVFLEILTYMLGGYWYLLCETLSICAHILAEDFQQALRNIGPAGKVAEYRALWLRLSKLARDTGIANCYTFTFM
SLYLFLIITLSIYGLLSKISEGFGVKDIGLALTAFCSIMLLFFICDEAHYASHNVRLNFQKKLLMIELSWMNADALTE
VNMFLRATEMNPSQISLGGFFDVNRTLFKSLLATMVTYLVVLLQFQISIPDDSRVQEADDDDDFVNATASVTE
APTTLTTITTLLTTLAKKKKKH*

>CpomGR4
MNASMTIFFQIMAKIFRFSRWFGVAGSGNVLWKTFGLCILLLLGVIEGVAIWRVVKALAGLAIDIEGHRSVTAR
LAGATFYASSITTLILSWKLSSSWETIASYWASIDRSIAINVSSDKKIKTRMITVTSVMVTCVVVEHAMSMMSQ
VGFECPPSLILKRYTLMSHGFLLLRTDYSIWFAVPLLFMSKIATILWNYQDILIVLISMGLTSRYNTLNQYVAKFST
LSKDPWNPHGECSKGHTWRRIREAYVKQAQLVRQLDQSLGGLILLSNLVNFYFICLQLFLGITQGLSGDLIKRLY
YVVSLVWLCVRVSCVVLAAADINVHSTKALRHLHASDRHYYNVEIVRLQNQLSKDYVALTGLGFFSLNRTVLLQ
MAGAIITYELVLIQFDDHGFTEHKSALNSTTF*

>CpomGR6
MRISYPWVFSFMPYSIPLGIITQFLHFQATFIWNFSDLFVICTSYYLTSRLDNVNRKLLIAQGKYLPASFWRSARE
EYSRVTQLIRKVDQVISGIVFISFANNLFFVCLQLFNTLENGIKGTGACRASRLKGTTLFAGYEGPAYFIFSLVYLIS
RSVAVSLIASQVNSASLLPAPVLYDVPSPVYCIEVQRFIDQVNADNVALTGLQFFTVTRELLLTVAGTIVTYELVM
LQLTPSAQVPTANGTMT

>CpomGR8
MKSTQSYNFLEIKKRNNYVEDEELHAEGALALLLRLCCWARVAPRRVHTRSGWRFETFASLASLQAFGMTLL
NAAIITSIILDFYQEPEKRLRVGATFLKTVIWLTEIALVMGIASLAVYTGPAQVECLKRVLKQLQKINSDLNLNNPS
AKTEKIKSIIMVILLTWVVFIMIMDVVFYYPHSLEEGTVCILCLQLPFYVAHLLWWQAVLRWALTVEAVHGAAA
TVNHRLQSFRLATMKPVSMTLDEFLSKPRSIGTLLNCIRDPALTAKTTELGIQNFTYPAQVKTLIRRLALSYERIG
DIMRQMNETNGLLLMIILTTTFMKLVVTPYYMLIYALDDESNVVFDMMLSLNWSLAILAILVLTIEPCHRVHSQ

RERTEVLLRQLTTHLAPSRQLSKELEQFTKLIVLNKPRFTALGIYTLDRPLMAMMLSGITTYLVIIIQFQKFSHKLE
YD*

>CpomGR9
EGPRICIDVPPVCVVSGALALLLRASSYAGVCPLRFTQTHDGWRPSPSAPLAAVQRLIMTIFNALMLAAFILDIC
QEPGQYIRIGETTLKMFVWCSDMLLMMMIASVAVYMAPKRMNHLVHMLDQLRQVSTELKMNPSARNEKI
KSIAIVFIPLWAASILIADFYSFLGPLMNGKMWYIMCMYGPYYVGNFMGILVLLQWSCAVLAVHATVVAVND
ELATLRRAKFDLGPTTTLEDLLRPPKPERNTLVGCFTKPAKSPGNSMTLPQAQATIRRLAFSHERISELMRQLNA
SNGVFLMFVLMSTFIRLVLTPYYLLQRFDHDERILYELLLQINWTLFHVITLLLTIEPCHWTQEQRERTQILLSHLI
VHLAPKCERLSKELDQFAKQILLSGAKYMPLGVYTLARPLMATILGGVTTYLVIIIQFQKISDQL*

>CpomGR10
AALILDFQEDPSKRIHVGESAVTACVWISDLGLVLAIASLAVYRGSARMKKFIKLLRELHKINDDLHNTKCVKME
KIGVIAVTSFLMSAMIIQVAQIYLLTKLFINRGCNWSIMLMYSSYYVANCLGLLALLQWGFVVLAVYSAAATVN
QHLLRLHHVKLKAKETAMSEVYLSPPKPRVDCFIEGHYDIAPPDFAAYPLQLQSMVRRLASSYGHIGELMRQM
NETNGTIIIAILMAVFLHLLVTPYYSLRALNSNANVLEVVLVPLCWTFLQIAILLLTVEPCHWTHEQRETTKFLLSR
VTVRLAPKSKLLARELDHFAKQICLSNIKFSPLGVLTLGRPLVASMFGGVATYLIILVQFYSHTDD*

>CpomGR29
MGYTSDLVLRFLRNYFPLKGILLVRAFFGHYFSFKCPKIYLKLHKVYCVVVTLVCVIIIFIMTNEWRKWVIFELVIM
TLASILIEGDCCGKFLSFVECTDHSFGLGRRNLASPRLYAAFFIITSIRLYIDYINIHAFFVNPVLYSAFTFLYTGLDLN
HLLRIIVFDILYERAKHLRNHFESVFSRVNGDDNMISEVKRGILLYKELIGSVTMLEKIQVTYLLALVARFAANVA
DIHLILCVEKRDEMLLGKMCRLGSESLYLAALVCAPAVIMELVHNEVDKITSILTIQHAIATDRELGA

>CpomGR30
RYFTYTIRLFNLKCTNFYKVFFPIRLLFNRDVIFFNNFAMEGILNFLKIYLPLNRILFIRAIFGHHFSFDCRMLYLNIH
KLYCIFVSFIFPLIIYSFSDVLESTRYAIFMEFFVCIWITLIVEDNCFQEYLTSIKRTDQLITHGRFNLASYRLYMVYFFI
TLLRMIIHFMSVKFFSISLYKFLTYAFVYMTLDLSNIIRVLIFEALYQRMIFLRNHFESIFDRPTNDCRNIISEVRRGL
LIYGQLLDSVKLVNKIQVTLFITLALRFVGFALKLNMMLFKVNSIWDHTRMFMYTFESAFILALDLTPAVFSELTH
NEVEKIETMMSHKHSMCTHRSLRAALSKGILYFKLRPFEFKIWRVIPVDSTLIFSFLSVFLTVSLLIFQFHRMM*

>CpomGR55
VNYKNRDVEALLKQQMKRFRIQLALSISIVNSITLMYFIEQYKSGKFSEMISTLIVIVFDLTLEYRFYFENIVFFVLI
DILVELLKYLNQSILSSIEKLNKDDIDEVGDTGRIANELEVWSEIIRLLAIACHRLQVCFGGQVLLSFFTTILYYIMYF
YQGILYSIKQELLWDSSISSVIMALSMYIAMKFVIVWSGQRAQNEAETLEANLTKLQTLLVKKRNLSRVLK

>CpomGR58
MNKNYKNNNGHVGCLLENYIEKEILDANWPLFFCQSVLLMPPFCVTNGYVTPVDKKYYIKILLGVCLHIAARV
YYYICLYESFLQISMNPFVIFMATANSATYCIAIIVIYFVNVIQSHNNLQVMLKLRQALFTINLEKQEILKDHKIWN
FIYIIGLVSIEVVVSICYCRMEQRMSFIFSKITFLVCDINLVCLYRTVSFGASLLASWNRKMMIYTNQIVTKKEIES
MFNAYMHIIDALGLCKK

>CpomGR60
MILDNVIDREFQMMLLPLNILEILYCQPKFRITETFITPNGIRENLLCTLGVLLMILANVGYVSINSYIPGNDEISDI
IHSFICTDAAFYIVYCLLMYAMNIIYKNQIVQLIIKMQKAYRVLQNENGLRRFQKSNWIFVVAVFLFYFTYNLSYT
IFNIYRVTHLLYDVVLFYFDVNIIVAIRIVKFLEYELILWKKELNKFLKTCSTTNHNQLVKYL

>CpomGR61
MENSRNIIDEKILDALSPLIFAQNFFLFPKFMITERCIAPIAPRSYTSSFVGAVLMLLIRIYRLVTVCFYNYFGENSD
ALLLANFVVGCFGTIFSYVINVVQSANAVYMVIELQEALWCLSSNIKQSLSDYKFWNIVNIACIFGGYILYTGLFG
VANQETHGEASFLVSHLVSITYDLNIILATRTVILTASILEAWNSKMSEILSEETEVRENCSQDMFSAYEKIINAFN
LCKKAYQFGIFYHTFQTFHSILYSMQLFLEYAKSASHEELKVFGLLRGVTYFAWNSKNFLLLVNVSVACERFYAAL
RDAE

>CpomGR63
MRVSGMRVISADRRSTELQNTFKPIKTLTSIVSLNCSGPNKSWQLFWIVLKALASASVLGCLTFYCLYIKIRYHYN
DVILSIKLTDVIQMSYDYSQYLVDLFFVFKYGRDTYAEYDKQLINIDQILISTNYSAIKRRHINLIVYFIAIWIFSSVC
DFTAWAVSYGSLLPTLYSTSYIYLLIKMISTLDLMSHVMHVEYRLKGIVNQLQECYCDTKPFPGDFSDPIGKKFW
FYCESPSKPGNTNETPPDRTLVCNSPQAVRWLSRCYLLLCEQCVFINSMFGTRILLNSLSLLIDMIRFTNIAVRLV
IGSQPTMYASGNYPAAANVLRMV

>CpomGR68.1
MIQFTVISFYYVLVLMVVGVLKNINEQMKSIYCSNRVNAQFIKVEKIITLNQIEVVYVHMLEMKREINRAFQASI
LATAIQCFHSIVSESHILYHGLVVEHTLTTHDVCNCSIWIVYQLIKIYIISCSGSMLKEQVSKIGRSLHNILPGKDDA
RLYLEVQHFSSMILYQNAEMTVYDFFPLDATFTFNVISAAVMYIVMLVQFDATKKS

>CpomGR68.2
MFTSLQKYFSPVVNEDEELCFLQIFKPLYIVLSALGLFPQAVRFPDGIQNTTLNIKNSVINSMCTLFMIVIVHAFL
VFHLQELNISSKDNSMTEDNMTLMNYIIGLVLEILFCTVSYFCVIRDRNLYITMLNDMAVCWDKLAMGKRRLI
LGRLRVHINCVVLTTVLAMILVLAVATYTSYLGVWKMILITLTFVLPDLIQFTMIAFYLVLMLMVVALFKNIEEEF
KVISLVKNNAPNDLVEAHLVVSIREIREIYVKTMEIKRRINEAFQAPILVAMMVCFLELVSMPHMIYHGLSFQAN
FTMHDAVECTIWVLNQLLKMYALAKSGALLNSQVNEIGRTIHNIPISGDKDLKLYLDVLHFSSLMTYQDTAITIY
GYFPLDSTLVFNIVASAAMYLVILVQFDKPE

>CpomGR68.3
MSSALTKYFSPVVNHEEELCLLQIFKPLYVLLSALGLFPLSIKFPDGIYKTNVDLKNSTINSAFTIFMIIVIHGSFVFH
LQELNISSKDNAMTESKMTLINFTVGLIIQVLFCTVSFFRVMYDRKIYITILNDMADCWERMAMGKRRLILGRL
RVQVNCVVLPSVLLSFLLILISQYTEIDLNIWKLILISLTFDLPELIQIAMLTFYFVLVLIIVALLKNIEEELILILHVAKN
NRNYPVEADMRMDMGEIMKVYVKTLGLKRQVNAAFQTSILVALMSTFHLLVSLPHLMYHGLTFQTNFSTHA
IIECSAWAVNQLIKLYFLSRSGDLMTSQVNEIGRTIHNIPISGDLDWKVILEVQHFSSLMTYQDAKMTVYGFFPL
DATLSFNMFASAAMYLVILVQFDKPE

>CpomGR68.4
MFSSLRLYFSPFVKKNEELRLLQIFKPLYLVLSFLGLIPCSLDLPQGNVDCIILHKSAFKHSCSAFLTLLIVYVFFGLH
VYEVLTSHEENILADDKMAKANYIIELVTQFTFCNATYFCAFRYKEIYVSILKEITRSWDDLPYVNRGIILGHLRVK
VNCGVIGTICLILLTLTAVTYAGSSSLWKRILITMSFNLPEMIQFILVAFYYVFVLMVVALLKNIEDHCRMFMKAR
RSIKNCSKVELGRIPITLSQMQCVYVKALRVKRQINVVFQAPIMFSLLQCFHTMVSESYDICQGLLYQDNFTTH
NLVECSYWVLLQMLKIYALARSGSLLKLEALKIGRTIHNIRSDDEEIKLLVEIQHFSTLMAFQSTEITIFGYFPLEAP
LMFNMVAAAAMYLIILVQFAKTH

>CpomGR68.5
LKIHKYLFLIIYFSISSSCTKHARNMFAALKKYFSPVVHQNEKLSLLQIFKPIYVLLSTLGLFPQAILFSDDGQDATFA
WCRALFMIILIHSFYIFHLHELYIFNKDNSITQGNMTLTNYIIDLSLQVLSCTVSYFHVIRDRNLYSRMLKDMAGL
WDRLAKGRRRQILGQLRVQMCALLCPGTVIIPLLLAITYRGSLRVWKKILFTVTFILPELIQFLMISFYLVMILMIV

ALLKNIEEEIKILALRNNICYNLLEADELGMSILKIKNVYVKTLKIKRQVNAAFEALILVALTVCFHELVGLPHMIYH
GTVYVPNFSINNTIGLSLWVFTQLLKMSALAISGALLKSQVRSLRS

Supplementary Data S15 - CpomGR non-annotated fragments

>c91528_g2_i1
IYFSGSELSILTGEVSVKKAETLIQRLALSYERMSNMMRQMNEANGLFLIVMLLSTFLRLVITPYYMLFLHEVYG
TIFSINWILVHLAVLALTIEPCHWTHTQRENTAILLSQLIVYQAPKCERLSRELDQFAK

>c67766_g2_i1
LLLRSVNYSPLGMATLDRPLVASVIGAVTTYLVILIQFQRYDS

>c2691_g1_i1
DHPVIIRKNVNGDPRLIVPPCEAIRRLAALHGSLCEVVQRIDRSYGLPLVVILLSTLLHLIVTPYFLIMEIIVSTNRIH
FLVLQFLWCVTHMLRMFVVVEPCHYTITEGKR

>c58408_g1_i1
FFCFLKLFTDSNPNLSANSALVFCVTNCITTLLFLRVATRWPRLCQLISKTEASDPSIDRTLIKKCRVSCVLVLTMA
LHYRHSIHV

>c5256_g1_i1
DTIRKKIIIITCVVSACAILEHVLSMMAATGFDCPPEEYFKRYILTSHGFLLNSYEYRLFLGVLIFFLSKL

>c21480_g1_i1
PSQRCRLIAWLEGHTLVFLGFFGLAPLKFETQSNGFAVTISSAMCIYSYALVTILVICTIFGLATEINIGVELSVRMS
SRMSQVVSTCDVLVVVITAGAGVYGAPRRMREMLKFMDNVASVDNSIGAQYCPANERKLCAILLAILIFFTVLI
TDDFCFYVLQAKKINREWDIVTNYIAFYLLWYVVMVLELQFAFTALSMRSRFRA

>c109326_g1_i1
PILFTLLQCFHTTVSECCDLCLGLFYKDNFSVQHIIECSFWVMLQLIKVYYVLARSGSLLKSAFRTEHCL

>c29823_g1_i1
FSLFVCVQADNIGRVIHDIPNSEEDDIDLFMEIQHFSTLMSFQDTEISIYGYFVLEAPLMFNMVAAAAMYLVILV
QFAA

>c91543_g1_i1
FSSSVLCFILYIAIVIIHLLWRNAENIRIVSNFAEIDSRLNLTLMENFYKTTRRYNYTIIFATLVLNSVYFVLYSLSRNIT
NYIYFYIVGSIYTLHDFEILFVCVLVHMLKVRLVIINNSLDRLVKENEKKHKAFATTIRQDKMETKLHELSQVYDVI
GETSRLINNLYNFQIFMSLISTFINVLLSIWVLLYSFQKEMI

>c92908_g1_i2
KHKFFSYSITNLTIMVHDAEQVFFGTMLRAIFERVRIVKAHVIKVCDRTSEETRKPKKPDKVEALSRKSQHAISSL
HEVYESLHKCSEQLNSV
>c92908_g2_i1_c92908_g3_i2
IFQANEESKDLLLLVYIFIRCLKYTILVIIPCYYSSKTTTQVSIIRKTLHDALNNDQFDKIDCRRLKAFFQLTRDSEFAY
ALWGVINLNMSLPLSYISLCTTYLVIIIQFSKFID-

>c71038_g1_i1
LYVQLNLSTEMKSDRVVKNKMEINRRFQTEYLMNNILDQDFQAMLFPLNFVQSYFLMPRFSIINNFITPDNH
VSFYIKSIIGCIILTSSHVFRFIYYSEVNK

>c75532_g1_i1
FILYFQIIISTCCSFFSFIINGYYVSLRMKYSRDSGVGVDHASIILIIGFVLIVVPSCLPAVFCELTHREIDKLKTAVGDL
LLFCEDDELQSELQLAIEYLDHRPFRVTIYHVWSLDSSFILAFVGLCTTYLITIVQIFGII-

>c91425_g2_i1
ASREPAHTLYFVPSTAYSVEVQRFLDQIHGDTIALSGLKFFNVKRGLVLTVSKYT-

>c4965_g1_i1
CERAYRQRDTALAVIDHILVGKDPDADLRSELTVLRGLIQSRPIQFHTAFFFRLEYGFLASMMSVMVTYTIILIQS
VN-

Supplementary Data S17 - CpomIR and iGluR Protein Fasta Sequences

>CpomIR7d
SVDMPIDGELNDDLMLFNKNITAAQNLAINAAKIALNNFEWRYVTMVFHNSSILLGLTAFMQIYRKSVIVGKG
TFLHGKESSADRISQFVIFGSDLVDIMCTLDWMRKREFDNTGKFIVICNNCDERKAMDIFWNHKILNVVFIND
SSGTSSLIGFTYSIYDNQKCVISPPEPLLDSCIHNSCMGVYPLKLRNLHKCQIIVSTFEQVPFMSLKTGTPIGADG
DLLLLIAEALNATLKVMTPHRGAGWGQLDKDGNWLGSLADVYHDLANFSMTSAAITLTRFKAFHLSTDYHSI
NMAWVTHPAVPLPGWQKLLRPFKMKARISLAVTFVLIILVAVFVKSNLWAKLSKRINTSARPQTCVLFYSWTIC
MGMPATSLPSKPTFLTMFLLWMFYCFMIRTFYQTSLIHAMKDNLNYPEFENLQDILNSGYPFGGVPALKDFYI
DDPEVYNNWKSINSTEINDMMVSLSRGMKYVLAMNKVTAQSFILKHYGDIHIVPQMIVTSPTVLYFKKFSPM
VQSLNLILDRLVEGGFTEKLYKNHASTHARKKTDSTAPMNFEQYMGCYVVLAAGWIVSILVFICEVYCYKFSV

>CpomIR8a
MDFCCLFLAIFIFNLGCVASELSLRFVFIIESHEQDLPQLIGRALKFAEEAQPDLRVSEAIVSLDRENEDESYRQLC
SALSNSVSIIVDLSWSPWDSLEELSSTAGVPLVRARLGSQHLVRAVDEYLESRNATDAALLMESEADVDRTLYEL
LGESNIRVWVHAGLTRDSARALKTMRPEPSFFVVVGSGAFATDTYKRAVKEKLVRRDYRWNLVLTDYSNLELQ
PVKPAMVLQVDAAECCKVMGQKDGCSCSQDFERKQPILSALLQLLAETYSKLDDDDFTTRVDCDNLVPENGT
RSKVYRQLAEELGASNESLFYWDGERSGIFLRSRFILSTLKPDIGPQHAAIWSADDEYKLLPGVTLEPLRQFFRIG
TAPAVPWTMPKLDSNTGEPMFNEDGEPMYEGYCIDLIQKLSESMDFDYEIITPKTGTFGRRLANGTWDGVV
GDLMRAETDIAVSALTMTAEREEVIDFVAPYFEQSGILIVIRKPTRKTSLFKFMTVLRTEVWLSIVAALVLTGFMI
WLLDKYSPYSARNNPDAYPYPCREFTLKESFWFALTSFTPQGGGEAPKALSGRTLVAAYWLFVVLMLATFTAN
LAAFLTVERMQTPVSSLEQLARQSRINYTVVEGSTIHQYFINMKFAEDTLYRVWKEITLNATSDQSQYRVWDY
PIREQYGHILLAINASMPVPDAKTGFRQVDEHTDADFAFIHDSAEIKYEVTLNCNLTEVGEVFAEQPYAIAVQQ
GSRLQEELSRALLDLQKERLLEQLAAKYWNETARQQCPDADESEGITLESLGGVFIATLFGLGLAMITLAWEVF
YYKRKEKNKVRQEDEETKPKKAFEKDLEKKIAGGVARLRKRDKKEKKGQVTIGDTFKPVSEKDGVSYISVYPKTE
YKP

>CpomIR21a
MRFLRTALFNYILLHYVISQEIEYYPSQASSFARKLVSEFNSEPYQHKHDLFKREAQWRKFNNNDDTEFTKNKT
QKRAVDPVFHGHPKTREELWNERIINESLAFDQTPSLISLIHNITLTYLNDCIPIILYDSEVKSKESYLFLNLLKDFPI
AYVHGYINENNELAEPKLVRATRECIHFIAFLSDVTKSAKILGKQAESKVVIIARSSQWAVQEFLAGPQSRMFINL
IVIGQSFKDGDDDTLEAPYILYTHKLYTDGLGASQPVVLTSWSHGKFSRQVNLFPRKMTEGYAGHRFVVAAAN
QPPYIFRTIKTDADGGNPRVVWDGIEVRLLTLLSQMNNFSIEIKEPREPHLGSGESVLKEITGGRADIGVAGIYLT
SDRIRDTDMSFSHSTDCAVFVTLMSTALPRYRAILGPFHWTVWLALTLTYLFGIFPLAFSDKHTLKHLLHNSGEI
ENMFWYVFGTFTNCFTFVGKNSWSKTTKITTRLLIGWYWLFTIIITSCYTGSIIAFVTLPVFPETIDSIQQLLDGF
YRVGTLDRGGWEKWFLNSSDPKTNKLLKKLQLVGDVPSGIRNTTKTFFLLPFAFLGSRAELEYIIQSNFTKTKKS
KKAQLHISNECFVPFGVSLTFPNNSLYSSKLSGDIARILQSGLMDKIENEVKWEMQRTPSGKFLSAGSGTLKLG
AITEKGLTLADTQGMFLLLAAGFVLAAAALISEWMGGCSRKCRPQKKEDEPSSAHSREHLIPTPKSDVDSEIKV
ISDSAESRFRLNPRPDSEDSRDSLEGTIINVTKESIIIHNNYHTSNWDSRRSSSVDIDKEVQEIFEKDEKRRRINSG
TVPLKDNQREATASKGAFGDHLSDH

>CpomIR25a
MASLIILLLFLFVPDSFSQTTQNINVLLINEENNALAEKSFEVAKEYVRRNPTLGLAVDPVIVVGNRTDAKAFLEN
VCRKYNDMLSAKKTPHVVLDFTMTGVGSETIKSFTAALGLPTISGSFGQAGDLRQWRNLDANQTKFLLQVM
PPADILPESIRAIVTKQDITNAAIIFDEFFVMDHKYKSLLQNIPTRHVITPVKSFNRDEIKTQLRSLRELDIVNFFVV
GSLRTIKNVLDAADENQYFGRKTAWFALTLDKGDISCGCKDATIVYMKPTPDAKSRDRLGKIKTTYSMNGEPEI
TSAFYFDLSLRTFLTVKSLLDSGKWPNDMRYISCDDYDGKNTPNRTLDLKTAFHEIKETPTYAPFFIPEDDPMN

GRSYMEFNTDLSAVTVKDGASIGSRNLGSWKAGLSNPLSLTDPQNMSDYSAQLVYRVVTVEQKPFIIRDDEA
PKGFKGYCIDLIEEIRQIVKFDYEITLVPDGNFGTMDENGNWNGIIKELVEKRADIGLTSLSVMAERENVVDFTV
PYYDLVGITILMKLPRTPTSLFKFLTVLEDDVWLSILAAYFFTSFLMWVFDKWSPYSYQNNREKYKDDEEKREF
NLKECLWFCMTSLTPQGGGEAPKNLSGRLLAATWWLFGFIIIASYTANLAAFLTVSRLDTPIESLDDLSKQYKIQ
YAPLNGSAAMTYFERMAHIEVRFYEIWKEMSLNDSLSDVERAKLAVWDYPVSDKYSKMWQAMKEAGLPNS
IEEAIQRVRDSESSSEGFAWLGDATDVRYYVLTSCDLQMVGDEFSRKPYAIAVQQGSPLKDQFNNAILQLLNKR
KLEKLKENWWNNNPEAMKCEKQEDQSDGISIQNIGGVFIVIFMGIGLACITLGVEYWWYKWRKRPIIGDVT
QVEPSKTTRNNADNSTTKIGEGFTFRSRNMGLSNFRSKF

>CpomIR41a.1
MIMPSKLFPVEILLNILINEHLQEYFCLTFVTETKLTVNIPINMSLMIIQPNNSVLAEQILDASEKGCSDYIIQMHE
PENFMIAFEKVNHLGDIRRSVKKLIFLPVQDDMNNRSVLTNILALRETGFVANILLVVPSLQSSGDCKVYDMIT
HTFVGSDEDVQKPLYLDRWDSCTGHFERGVNLFPHNMSNLYGKTVKVAAFTYKPYVLLDLDPSLNSLGRDG
MEMRIIDEFCRWVNCTVEIVRDDEHEWGEIYENNTGVGVLGNVVEDRADIGITALYSWYDEFRVLDFSAPIIR
TAITCVAPAPRILTSWDLPLVPFTWTMWMCLVFTFFYASFALSIAQRSTDNVFLDTFGMMITQTREDATSWRI
RSITGWMLVTGLVIDNAYSGGLASSFTVPKYEASIDTVEDLVDRKMEWGATHDAWIFSIMLSEEPLIKSLLSQF
KTYPADILRQKSFSRSMAFSIEHLPAGYFAIGEYITKEAAMDLEIMLDKIYYEQCVVMLRKSSPYTAKLSELVGRL
HQSGLMLSWETQVALKYLDFKVQLEVRLSRARKDLEEIEPLSIKQLLGIYIFYFGGVVIALLVFFGELLSKCSKPSIV
L

>CpomIR41a.2
MVKMLIPSTIYFPIEILLNTIINNYLQTSFCLTFVTETELMINLPLNMSSMRIIPNNSELVQQILETSEKACTDYIIQ
MDEPRNFMIAFDKVNHVGDVRKSDKKLIFLPLEDEFYNPSVLTDLLSLKETGYVPNILLITPTGQKSSDCKVYD
MITHTFVGAEEQIQNPLYLDRWDCCTEVFEKEVNLFPHDMSNLYGKKVKVGAFTYKPYVLLDLEPSLAPLGRD
GIDIRFIEEFCRWINCTVEIVRPDDGQEWGEIYENNTGIGLVGNLVEDRTEIGITSLYSWYEEYRALDFSAPIIRTA
VTCIAPAPRILSSWDLPLVPFSWLMWMCLIATFFFASFALFVAQRSTDDIFFVTFGNMIGQSPGDSSSWRIRSIS
GWMLVTGLVIDNAYSGGLASSFTVPKYEASVDTIQDLVDRKMEWGAPVDAWLYSMILSEEPLIKSAISQFKVY
PPETLTKKSFTRSMAFSIERLPAGSFAIGEYITKEGAKNLELMVEDMYYEQCVVMTRKSSPYTAKLTELVGRLQQ
SGLLLCWETQIALKYLDFKVQLEVRLSRTKKDIDGVEPLNVKQLLGIYLLYFGGLSISIVVFIAELLIKRGKAVIVI

>CpomIR60a
GFYVQATMLKIICLLSIGVNAKVNPHGPTVVSDFSSCVSEIIDKNFAQSGLLFFVDTFNVSTPVAGIRNGIIKSVH
TKLKYSVKIAIPTKKDKGICVNNDKTAIEISVKSRMDHFEATSLADYFILIIEDYKDFSYIASRLIRAISWNPRALFILV
YFSISNSDDQNIRHAEDMLFCLFKVNVINAVVIIPEVNNVRRANIYSWRPYAPPKYCGHYNESIRNRLIVENVC
ERGKIKYAKKIFESKIPSDMMGCSLKVLALERQPFISHNPLDPNIESLLINQVAKRYNLSLRYEILNSFRGEKLFDG
DWTGALKELTYKKGHLLLGGIFPDDEVHEDFECSSNYLADSYTWVVPRALPKPAWLALFVIFQKTVWLTVITCF
VFIALSWMVLAKLSKDPTYRTNLDHYFINTWLSNLGFCAFSRPITNSLRLFFVFINIYCILLLTAYQTKLIDVLTNPS
FEYQISTVEELVESGLKFGGSEELHDIFENSTDSIDNYFLDGWIDIADIRDALRDVAIHRNFSLMCSRLELAYVSAI
IPELSDQFGKYMYYAFPTNVFTVPLEAVSMKGFPFMKGFSRTLTHFEQHGVNNGVIVYFGGYLLRQRALLLNK
FKIEHNSRDPLSIQTLQGGYLALMFGSVCGTFVFIVEIILNTKFVKKLKIL

>CpomIR64a
MNLTTYSALFTIFSTAEINLITDVFKHKHLHFGTIFHCSKPENAIFLQKHLKKMDLRFSTIMMHSNASHFKQTND
SRVGIVLKTSCENWTQVFEHFNCNLFEKSLYSWLIFTDDLSSASEALSRYPIEVDSDVAIIYRQEKSYYIYEVYNTG
YFTNGRYHVEPVGYWYYKLRIKGHRRTNLDGIVLRSAVVVTHSIGHQTFEEYISRLKPEVDSLHKLKYFTLLNYL
RDMYNFSLIVQRTNSWGYVTNGSFDGMVGTLQRGETDIGGTPVFIRADRAKFIYYVTATWPSKPCFIFRHPK
HPGGFLTIYTRPLSYNVWLCIIALLVFAGSLLCVLIKLRVTRTAGDDGDLSASLALLSIWSAVCQQGTTVNLSANS

VRLVLFFSFLFSLFVYQYYNALVVSTLLRAPPVTIRSLEDLLRSKLKAGVEDVLYNKDYFRRTTDPIALELYSRKIASS
PRPNFLPPDRGMALVKQGGYAYHADTAYSYPIIRRTFTEREICELQEVELFPPQTMFAVMKKGSPYIKHLSYGIR
KMAESGLMQRLKTIWDEPKPLCVRTPDSSIFSVTLREFITPLLLLCLGMLAATVVFMAEHVFYRLQWKRIQFR
H

>CpomIR75d
LQVQTRIFAGMELVSFALAYFAAKRLSLLTAFLCWRPEELSALCRDAQRQGMRISIADWTHLPPLEPYATHREG
MLLDVTCPDAPLVLEKASSTRAFNLRHTWLLLHNAPFNASLMEVTLDSTLVLPDADVAWVANDQFLDVYRIK
HDQALITMPLGDDAARVALPAAPTRRRDLNNVYLRSSTIISQPQFFKGWNDLTVRQIDTFPKLTWPLMHLLA
DDLHFRYNLIQVDLYGENRNGSFDGLAGQLQRQEIEVGITSMFLRADRMQVLHFCSETVELRGAFMFRQPSK
SAVSNVFLLPFSRGVWIATALTLVLAAVTLALLARRPRLXXAVDASLEQLSIGEAVIFTVGTACQQGFHIVPELAS
ARVVMFCALMTALFAFTAYSAKIVAILQTPSDAIRTIDDLTNSPMTMGVQETTYKRVYFAESTQPATQRLYRHKL
LPLGDRAYLSVVDGVAAMRTGLFAFQVEEPSGYDIISKTFTEREKCGLMQIQAFKLPMVAVPIRKHSGYKELFA
TRLRWQRETGLMDRTRRIWLASKPRCDANSGGFVSVGIIDILPALHVLAAGMVASVLFLVLERSMARLKCCGV
RGS*

>CpomIR75p.1
MDIWKLGAIIWLFKSHVEGRAGIGKFLTSFVDNERKPTTVVFHGICWNNSVKLHVMKELSKAGIRSSQSMSK
RTSLIDHTVLLLADLNCTGTDDLIINATQRELHRLPYRWLVLSDAPRFGRSSLWDLFLVDSELVLATVDGAGYSM
TEVYKPSPTSPAILTPRGTFHHVLTDTRPHRELFRRRRDLMGVPLTITNTIQESNSSIYHLLQEDSLELEHDLISKN
SYTLAKVAFLTLNSTPVATFTNSFGYLQNGQWTGVIKELLEYNADIGTNVGMSQSRLKQVMFLDPLDNGRAR
FIFRQPALSLTANIFSLPFSPDVWIATGLSSFVAGVAYYLSTRLIKTRAEKGTVRDAYLLTMSALSQQGCEVQPRH
VSARIVLWVVFTSMMALYAAYGANIVVLLQAPSTSVNSLATLAKSKLALGAADVNYNHFLFRASSDPVRNDIA
KRINSDKGPKAFYGLTEGVEKIRKGLFAFHSVVEPVYRQIDRTFQEKEKCDLMELDYIGYAAFHVPGSKKSPYLEL
LRVTFKRLREVGIKSAVNFRYEARRPSCKESIAMFSSVGITEMRPVLIFMAYGVALSVAVTAAELLVFHANRYRL
RQQRLAVQLGRI

>CpomIR75p.2
MKILFSFVIVLFLSLGKAFDDNDINMIVSFVTLDERATAVLTPYVCWSTYELTSLAKSLHDTGISMAASLQPKRPE
LFLQNLVIVADLRCRRTDDFLIKASDEGFFKSPYRWLLISQDQTELNVLDQLAMLVDSDVVIAQRRGADYQYVE
VYKIVENSQLIYNTRALWRPIDKNNNTAIITYYNKSKVVANKYGAVEDYRKSKILSTRRMDIRKHTLTMVNVITD
SNDTRKHMDDRLNLHQDSITKMSYMVVKICFEMMNSTEKLIFTNTWGYVDKNGSWNGIIERLIKKEGDIGTL
TIFTQERLKIIDYIAMVGTTAVRFVFREPPLAYVSNIFALPFTGAVWLAVFICVLACALFLYITSKWEATMGIHPM
QLDGSWADVLILIIGAVLQQGCTLEPRRAAGRIVTLLLFIALTILYAAYSANIVVLLRAPSSSIRSLQDILNSPIKLGA
SDFSYNRYFFKKLNEPLRKEIYNKKIAPKGKKANFYTMKEGIEKIRKGLFAFHMELNPGYRLIQETYQEDEKCDLV
EIDYINEIDPWVPGQKRSPYKDLFKINFIKIRESGIQNCIHQRLHVGKPRCLGAVNTFSSVGIMDMYSAMLATLY
GMFMAPAVLLLEIAYKRLMVAREKRMQHNNSHSHT

>CpomIR75p.3
VRRRDLRGADVVLPTVLLHNQSLEDLPDYLHRERDTLTKVVYYMCTHLVEWVNGTKIWNRTTSWGYLQPDG
QWDGIVREMQDGRADIAGSCMMAQKERVKYVNYVLAPSKIEAMFVFKKPALASVTNIYVLPFDIGVWVSII
VLIIISSSTLFLSSFGEDRMKNYYSKSWLQKMSDGFFDTLCLMFQQGTAADPLSIASRQILLLGLMAFMFLYTAY
SANVVALLQSPTNDINSIETLLTSPVACGSQDVVYAWQMFGHESRPIHRLLADRKINSQGKKGFLSVEDGIRKV
REGMFAFHVEQTAGFDQIQKTFLEDEKCNLGFIKYMSTTSPFVAFSQITPIKEMLRIGANRIMEVGVQSRSARR
LVPERPRCGASAAMFNAVRLSDVAPAFRVLLAFYALTLPILGLEILVKRREMKRGIFESKSVDTATAEITNNIII

>CpomIR75q.1

MYTSLTIVCAIFICSSFALSISKENDIKVIVDVIQSFNKPTDVISNVCWTNIYKKKLTANLAAADNPRSIKFVNDIHA
KDLVHPEKVTFLIDVRCKDSGGFLNKAASRKYFGRPHRWFIINTPANEVSVPLVIDKMHLLPDSEVYVMQLIN
NSYSINLIYKIKPNREWIIENYGNWSTENGLTISRRAKKVALVMRRRNLARASIVTSMVITDNGSFADLETLRYK
QIDSVTKGGFHQLTALYEFMNASREFVFTDQWGHHVNGTWHGMVGHLADGTAELAGAILFITKERMPLIEY
MSHPMESSIKFLFREPPLSYQNNLYLLPFQASVWYCVGSFVLVLIFAMYFSAYWEAKKVADEKQKADDTTVLV
PTISDVTIFVMCAISQQGSTVELKGMLGRFMILILFLVFLFLYTAYSASIVVLLQSSSNQIRTLTDLLNSKLELGVED
TPYNRYWFMNEKEPIRRAIYEKKIAPSGSKPKFFDLTEGILQLQKKPFALNCNLGVAYKVMERYFYEHEKCGLQ
EISYLQNNNPWQAVRKGSPYREIFKIGLLRNAEFGLNDRTNRIMFSKKPVCSVRGGSFVSVSLVDCYPILLLLLY
GMILGVMLLLVEILYYRKMNTRP

>CpomIR75q.2
MVADVIRAMQRPSAVIAMLCWSSNLKLQLYSALEGENVTQITMMQFLKAGTVPERHAQDQHIVFLADLDC
PDIISYFQTSSLNKHFRSPFRWILIDSGNNDTSQSYIPNAVGNFDILVDSEVILAHHLGDGSYRLHLIYRIGNNTD
WKKEFYGTWDERRRLQKQVMEGEIILRRIDLESYELPICYVLTDNDSINHLYDNVNDHIDTITKVNFPTTNHLLD
FLNASRKYVFANTWGYRVNGTWNGMTGYLVREEVEIGGSPMFFTSERISIVDYISSPTPTRSKFVFRQPKLSYE
NNLFLLSFRASVWYSSIALLLLLVIVLFIVTIWEWKKTRGHEDKKLEADSGILRASVVDVVLLIFGAACQQGSTVE
LKGSLGRIVMLILFLALMFLYTSYSANIVALLQSSSSQIKTLEDLLHSRIKFGVHDTVFNKYYFSTATEPVRKAIYET
KVAPSGSKPRFMPMDEGVKKMQKGLFAFHMETGVGYKFVGKYFQESEKCGLKEIQYLQVIDPWLAVRKNTP
YKEMFKLGTKRIQEHGLQSRENRLLYEKRPKCSGQGGSFVSVSMVDCYPALLVLFYGAVFSVGLLFIEILTKRRN
DILRKISRAKTLGVDVEDY

>CpomIR76b
MTGLELIVSSICNATFCEVVYDNPITDTLLPAQKKELLKIAEDLNGKHLKIGTYDNYPLSWVHTEDNGKLTGRGV
AFVVLDILRERFNFTFDVVTPLKNFEIGIEGRMEDSLIGLVNSSQVDMAAAFLPIVYKYQQFVDFSSILDKGVW
MMMLQRPKESAAGSGLLAPFEIQVWYLILAAVLSYGPCITLLTYLRSKLVRDGEKNISLTPSFWFVYGALLKQGT
TLAPEANTTRILFTTWWLFIILLSAFYTANLTAFLTLSKFTLDVEYPEDLYKKNYRWVAPEGSTVQYVVNDADEN
LHFLSKMVANGRAEFRSVNADRQYLPYVMGGAVLVKEQTAIHHLMFEDYLKKTKAKVPETKRCTYVVAPNPF
MEKLRSFAFPKNSKLKLLFDPVLTYLLQSGIVTFLEFRDLPSTKICPLDLQSKDRKLRNSDLSMTYMLMGVGLAT
AIAVFGGEMIIRYYVRIKIRKNRGERTRTKTVKTSKHRRFRIQDDSHPPPYDSLFGQNSRYKMNGDSTTKIINGR
EYWVVGTVSGDIRFIPVRTPSAFLYQRDK

>CpomIR87a
MLLLRTIHSGIKYSVMVKDSFYKHANASHFPEKAKNYMLILEEKSELVRNILQLNKLPTWNPLAKAIIYYQLLPD
EDGETIAKKFINELREYKLLKSIVFIYSPDDAGLISYTWAPYSDTNCGGECDSVYILDTCKNSIVKQKNAQREMFP
LNMKKCPLVTQAIISEPYVMPPVRQLTNTSYPDAYEFQKGGEINLVKLISEFTNMSLIVRISDVPENWGLIYPNG
TATGAYGILRNDSVDLVIGDIEVTRTIRKWFHPTVSYTQDEMTWCVPKSAQASTWNNLVIIFQWTTWVATLLS
IVTMGLIFHYIYYRENDRKVTKLPTNSLLNTFSMILGWGASFKPKTATFRILIFAWLFFGMIMSISYESFLRTFLM
HPRYEKQISSETDLIQSGIPLGGRAIYRSYFETNNASSFYLYRKYISTSFSEGIKRAALERNFAVVASRRQAEYQDQ
KLGKGEQLLYCFKEGNNLYKYGVVLLARRWFPILERFNNIIRSVSENGLIEKWNQELFIHTVGVDGTSKVVPLGI
RHLLGAFIFIGIMYAASVIVFVVELLLNVSKKRKGNKPICRACLSSKYSSRR

>CpomIR93a
MRIWVVLICVVGVRGEEFPSLITANASIAVVLDRQYLGEQYQPLLDTLKDYIKELARVELKHGGVVVHYYSWST
ISLKKGFIAVFSIASCEDTWSLFSRAEEEELLLFALTEVDCPRLPPDSAITITYTDPGQELPQLLLDLRTTRAFNWKS
AVILHDDTLNRDMVSRVVQSLTSQIDDEDVPTISVTVFKMRHEINEYLRRKEMHRVLSKLPVKHIGENFIAIVTS
DVMSTMAETARDLFMSNTQAQWLYVISDTSIRNSNLSSFVNALYEGENIAYIYNITDDREDCKNGLMCYSEE
MMNAFISALDSAVQEEFDVAAQVSDEEWEAIRPTKIQRRDTLLKHMQQHIAVNSVCGNCSTWQAMAANT

WGSTYGGNVQADNVAAPDNETNEAIQKIELLQVAYWRPSDGLRFTDFLFPHIVHGFRGKVLPIITYNNPPWTI
LKANESGSISSYSGLIFDIVDQLAKNKNFTLKLIFPGDMKDVLSNKTVTNDMYSQSAKLTMMAVARKQAAFAA
AAFTVLSDRNPGINYTIPVSTQSYAFIIARPRELSRAMLFLLPFTTDTWLCLGFAVVLMGPTLYVVHRLSPYYEA
MGVTRQGGLATIHNCLWYIYGALLQQGGMYLPRADSGRLVVGTWWLVVLVVVTTYSGNLVAFLTFPKQEVP
VTTVSELLENRAVYTWSISRGSYLEFELKNSDEPKYVSLLKGAELTSDSSGLEGNLASGSPLLSRVRQDRHVIID
WKLRLSYLMRAEHLATDKCDFALSAEEFLDEQVAMIVPAGSPYLPVFNKEINRMQKAGLITKWLSAYLPKRDR
CWKTSSVTQEVDNHTVNLSDMQGSFFVLFLGFFSASSVLLMEWFYHRRKSQKEDVAIKPYVE

>CpomIR1
RMTMWRLLFLVASAASLPTDWPHMAVDYFQHKHVKYVAHLSCKDAAEIKGVLRLLMNEGIRAAVGLIDQGP
MNIMPLLYQYEASVGVLVDGDCINTRDILNNASESMMFDDTHFWLVMNDNCSMGFVEDTFLDLKLSVDAD
VVVASYCGDIYQLTDVFNFGRVQGNVLETRELGAWTSERGLEIVLQGFKYYNRWDFHNLTLRAVSVIRNSSKE
FHEGMLYEPGFTVGVAAMTKISSQLLNLLKEMHNFRFNYTIVGRWIGTPERNSTKAMSNMLLWRDQDISST
CTRLFSNWLDWMDPFFPSVTELETKFYYTISEKGIGDYENQFLTPMSPEVWWCAAATGVVCALXXXXXXXXX
XXXXXXXXGVFSVLAAGFQQDYEDGHQTKKDSSSRKLALLVVGLTSMLMYNYYTSSVVSWLLNAAAPSLDSL
DGLIKSDFELVFEDIGYTRQWLDNPGFFYYMGYKNEKEDELRAKKVTNVKRTLPLFESIEDGIELMRTGKCAFH
TEPYTASQVISRTFADKDLCSLAGLQIMPPSYVYVMGQKNSPYRQFFVWSMMRLLERGHTRATRARVGGQI
PPCSGLTPRSFKIFSI

>CpomIR2
MEEETFTVASTPDCMTVLQDERNDPLWRETQVGCKVLLLLQEIHKFNITYSKGPYHYNERGFYEYVDEVDIYA
KPRSLWSPVMVNYTPIAPIMDWKFGYILRHPFNIQHFYSLAFSKPAWHFIVAMILLVSVLFYILNRAEQKLTGE
NLKCYFWSELLIAFGIICQHYISINPMELTSRRIAFISFFMFSYILYSYYTSTLLSDLVYDRDNEMDLETLAESDYEH
AVLDSVTSVFKVLVEQLRNNNKMFPQRQSFTENKLINHRVVNISTGLAEVKMSKTALLSDYVSIHSGVIQWFS
ESEVCDLIKVDIFSNVLKYLVTSKKFKYIDEFKISTLRAYEAGVLQRLLSPHPIQHFSPTCVSSHFQADIGLVRKPFIL
LALGYVLCGFILLVERVYYNRYKVWPYVN

>CpomIR3
LSAKMVGKSILFFLLSSVAGLEDRDIDFSVDFLKARDVKYICMLTCGDRTWNKKFAKNASKSSIAVSYVRIDDSL
SDLDSVRVCLSPEFTDVGVLIDTKCPLYEEVLMYASENLLFDANHKWLIIDIDTWISNISTVFNVETNENFSWLM
NTLEKLNMSIDANVMLSLQKGSENNIYEVYNFGKLRGGNVVVKKLGNWRNRADLIQHLNAYKYYRRWDFE
NFTINYVAVMSTPPKVFDVNMLVGDTPAPGVAVMTTTVTRVLLEIAELHNIRYNYTIVDRWIGKFERNTTPVV
ATLLYFKEQDITPVLRVTSEVFQRVDMVSPPITSIETRYYYRIPTTGPGKFENQFLRPLTKGAWGCVIAVISLCALV
LFLTVRAETRPAALQYAVFSVAATFCQQFFEDGGYDDPRRESSARQLTILVTGASCVLIYNYYTSSVVSWLLNGP
PPSINSLQELLESPLSLIYQDIGYTRSWLQNPKYYYNKKNSEVEDQLRKLKVFKKKKGEPLLVPLEEGIEMVKAG
GYAYHTEVYNANMLISRSFNQEELCELGSLQSMEETPVYIAIPKDSPYKEFFNWKLVAKNELKFIGKMKHRILQ
GHE

>CpomIR4
MIIPAVAAFFKYKIVSSIIIFTCGNEFEQIRLVRQLSLQGMRATVSCDPGILNEEHKTLQGVLYFNRPNDTLLDETS
WEHFSMWYKWLIIGNEVPSRLNHTTRYDADITLLGLRQLGAIDAIDNSSVAYHESILFEDLYVHLRDGVSRHP
WAVWTPAGFQPLYELERIRRRHDLKRYTMRIPTPVGHYDDSYEGTFADYVMDNSQPGRDSAIRCGYGTSSLIL
EWLQAKEVILQMEQWSTDAGNKSMFTRLAQGTSELSGGILRMQHKRLLKLDYVIPLWIFKVGFTYVAERESS
SNMFVIPFTGTTWAACAVVTLVLAIAQRATAKQESEKEGAFVAVMATWLQQDASAVPEGASGRITFMALSIC
SMLVYAYYSSAIVSALMSAGSSGPTTLRALGDSRYRLASEDYEWIRAQMFDVYIPNWPEMEYLKRKKLQSMA
NFYLDWQAGMQLVKSGTTAYHAEYNHVYPLMSVLSDDQVCKLQYVDTVPPIMSWLVTTRRGQWTNLLRIG
GDWLHETGLVKRMLSRWQLKPPPCRAALLAERVSYGDVAPLIILTVVGLLTSVAVLFLERAVAKWRAKKTDKSS

SDKIQDEEIIDTVMS

>Cpom-iGluR1
MRSLLGIIAALSCWQLASADRDQRRYSNPTYYNVGGVLSSNESIAFFKDTISNLNFKDQYVPRGVTYHDYSILM
DPNPIKTALNVCKDLIGHRVYAVVVSHPLTGDLSPAAVSYTSGFYHIPVIGISSRDSAFSDKNIHVSFLRTVPPYSH
QADVWVDVLKHFNYMKVIFIHSSDTDGRAILGRFQTTSQSVDEDVDRKVMVEQVIEFEPGLDSFSDKLIEVKT
ARSRVFLMYASKTDAEIIFRDATFLNMTTTGYVWVVTEQALDAANAPEGLLGLRLVNATNEHAHIQDSIYVLA
SAIRDMNTTEEINAPPSDCDNSGSTWNTGRHLFDYIRKQTLESGATGHVAFDDHGDRVNAEYDMVNVRAQ
GEHVAVGKYFYSKDVRKMRLELKEQEIIWMGRSPSKPEGFMIPTHLKVLTIEEKPFVYARRIDDGSDCTAEEIPC
PHYNTTDDTDQMYCCKGFCMDLLKHLATWINFTYSLALSPDGQFGHYVIKNYSAPSPKKEWTGLIGELVYER
ADMIVAPLTINPERAEFIEFSKPFKYQGITILEKKPSRSSTLVSFLQPFSNTLWILVMVSVHVVALVLYLLDRFSPFG
RFKLANIDGTEEDALNLSSAIWFAWGVLLNSGIGEGTPRSFSARVLGMVWAGFAMIIVASYTANLAAFLVLERP
KTKLTGINDARLRNTMENLTCATVKGSGVDMYFRRQVELSNMYRTMEANNYDNAEQAIQDVKNGKLMAFI
WDSSRLEFEAAQDCELVTAGELFGRSGYGVGLQKGSPWADKVTLAILDFHESGIMESLDNNWILRNNLLNCE
ENEKTPNTLGLKNMAGVFILVLAGIIGGIVLIVIEVVYKRHQIKKQKRLEIARHAADRWRGTIEKRKTMRASIMP
SQRRAKSNGVKEAGSISLAVDRGAIRRRDEPRVPRYLPAYTPDVSHLVV

>Cpom-iGluR2
MAYERKVRCSVGAGLLLLYVGVAAQPFSVEKIVVGAIFDQNTEEIQNVFKYAMNTHNQNISSRRLELQAYVDV
INTADAFKLSRLICNQFARGVFGMLGAVTPESFDTLHSYTNTFQMPFVTPWFPEKVIPPSSGLIDHAVSMRPD
YHKAIVDTIVWYGWTEIIYMYDSHDGLLRLQQLYQSMQPGRTVFRISLVKRITNASEAMDFLLALEQHDRWG
NKRIVLDCNAKNAKSILVEHVRKVQLGRRTYHYMLSGLVMDDHWENEVTEYGAVNITGFRIVDHSRKIVRDF
MDGLRRMDPRFKGTISAQAALMYDGVQVLMDALGRLSRKKPDAFRNALRRAAGQANSTKVIDCNPGKSW
VIPFEHGDKISRLIKKTDIEGLTGNISFNEEGHRHNFTLQVVEMTVQSAMLTVGTWTDANGLSVSSPKQVQLK
SPASYDTNKTYIVTTILQEPYLMQKSKTGHAMPETQYHGFCKDLVDLLAQNLKIKYELRLVQDGTYGSETPPTG
WSGIVGEIVRKEADIAVAPLAVTPEREMVVDFSEAFLSIDYPINHAKTPKQLADTFSFLRPLSKEIWLCVLFSFFA
VSIVLFLVSRFSPHEWRSVSITDTHLDHPISSANEIILHNEFSIWNSFWFSLGSFMQQGSDVVPKSLSGRIVGTV
WWFFALILVCSYTANLAAYLIVERLSEPSQSIISPPSMPQTEQNSIEGTVLNEDLAEYSFEKEGQLVCGSMPRAC
RYKHVDFAIATAKGSPLREVINLAIVEMKASGVISKLWRKWVLETRKSDCDEGKDEETSITEMTLSQVAGIFYVL
VGGLALALGVA

>Cpom-iGluR3
FRIMFRMIQIVLLVLIIMYFNSVMGGQVRDKDKGEKVPIGVVFDQNTEEIQNAFKFAIMQHSTLNKTSKLDFQ
LYVDVINTADAFKLSRLICNQFARGVIAFLGAVTPDSFDTLHSYTNTFQMPFVTPWFPEKVIPPSSGLIDYAVSM
RPDYHRAVIDTITHYGWKHVIYIYDSHDGLLRLQQLYQSLQPGNATFRISNVKRVNNASDVVVFLAALERLDR
WSNKYVVLDSTTQLAKEALIQHVRDVQLGRRNYHYFLSGLVMDDRWEKEVAEFGAINITGFRVLDFSRKMV
RDFIDEWKRNSISAQAALTYDAVQVLVDAILRLLRKKPDIFRSPIRRNANVNASRILDCNPKGDKIIPYEHGDKIS
RMIKKTEIDGLTGVVRFNEEGHRKNFSLQVMEMTVEGEMIKIATWYDNKGFVPVVPKLPGPSIPGVYNRNKT
YIVSTIEEPPYIMRQNSDYAEFTPNDPYKGFCVDLAKMLSDKLEIKYEIRVVKDGKYGSENPKIIGGWDGMIGEI
LRKEVDMAIAPLTVTVDRETVVDFSKPFLSFDIKPSLKNIADEPGAIFSFLDPLSTEVWLCLMFSVLAVTVVLFIV
SRFSPYEWRVVSYTDTQSSEHTEVATTKTTVVNEFSFWNSMWFSVGSFMQQGSDITPRSVSGRIVGTVWW
FFTFIVISSYTASLASYLTLQRINEPSQTYSKVAACPEDTSEGIKSIAIPRSRHNWLSFLLDHSSAGDDADKPCEML
VTVTNSGVKDFAVALQKGSELR

>Cpom-iGluR4
VVPHKAFGARDYTKAEKAALAKLPRKLKLFSQVRLNVTLSMQGLTPSPMSILDSLCKEFLAVNVSAILYLMNHE
QYGRSTASAQYFLQLAGYLGIPVIAWNADNSGLEKRASHAALRLQLAPSIEHQTAAMLSILERYKWHQFSVVT

SAIAGHDDFIQAVRERVTALQDRFKFTILNAVVVKKPTDLNELVTSEARVMLLYATREEAADILSTAGDLHLTSEN
FVWIVTQSVLGSMQQPNKFPVGMLGIHFDTSSSSLIAEIATAVKVFAYGVESYILAPENARHPLGTRLSCSGAG
ASEARWSTGERFYQHLKNVSVESEASRPSIEFTPDGELRAAELKIMNLRPAIGEQLVWEEIGTWNSYPKERLDI
KDIVWPGGLHTPPQGVPEKFHMRITFLEEPPYINLAPPDPISGRCILDRGVICRIAPEADVAGLEAGTAHRNSSL
YQCCSGFCIDLLQQLAEQLGFTYELSRVEDGRWGTLHHGKWNGLIADLVNKKTDMVLTSLIINSDREAVVDFS
VPFMETGVAIVVAKRTGIISPTAFLEPFDTASWMLVGAVAIQAATFSIFFFEWLSPSGFDCSTGQDSNRAPQNR
FSLCRTYWIVWAVLFQASVHVDSPRGFTARFMTNMWAMFAVVFLAIYTANLAAFMITREEFHELSGLDDPRI
SRPLTIRPPLKFGTVPWSHTDATLAKYFQEPHAYMSQFNRSTVGAGVQGVLTGELDAFIYDGTVLDYLVSQDE
DCRLLTVGAWYAMSGYGLAFARNSKYLSMFNKRLLDLRSNGDLERLRRYWMTGTCKPNKQEHKSSDPLALE
QFLSAFLLLMAGILLAALLLLLEHVYFRYMRGHLAASTVG

>Cpom-iGluR5
MWAWCGWLVAVHAVAAVSPVIKIGAILTDEARGGSTELAFKYAVYRINKERSVLPDSTLVYAIQYIPAGDSFRSY
KKACHEIKSGAVALFSSGGPTLSRSLNALCRSLNIIHLSSSADTASQEDNDPDSFTVNLYPARQVMDRAFADLTA
YLNWTRMGVIYEDYGYGELNIANIAKDGRDMYCVRASHEYRRALTLLKAQGVTHIIVDTDPKKLRQLARAILQ
LQMNNENYHYIFTSFDMELFDLEDFYYNRVNMSGWRLVDRDSDKVKDTLLVMEKFHPIGASILTGGHIKTEP
ALLYDAIQVLAMALAASKEVGPTNASCDDETIWSNGKDIMENINKIHAHGLTGPIQFQNGVRTNFSLQLMRL
VGGEKGGTVVSGHWNPGDGLTITDPAAYKRDPPPNVTLTIVTVEEKPYVMVKEGWNLQGNARFEGFCIDLL
ARVAAQAGFAYRLRLVPDNMYGARDPDTGQWNGIVRELVDRKADIAVASMTINYAREAVIDFTKPFMNLGI
GILFKVPTSQPTRLFSFLNPLAIEIWLYVLAAYILVSFTLFVMARFSPYEWSSSTHVCGHETKLLTNQFSVCNSLW
FITGTFLRQGSGLNPKATSTRIVGGIWWFFTLIILSSYTANLAAFLTVERTVLPIQSAADLAAQNSVQYGTLNGG
STMTFFRDSNIDIYQKMWQHMSTTSPPALVSSYEEGVRRVLQGNYAFLMESTMLDHRVQRDCNLTQIGGLL
DSKGYGIATWKGSPWRDRISLAILELQEKGVIQILYDKWWKNTGDVCNRDGKDSKANPLGVQNIGGVFVTLL
CGLALAIVVAILEFCWHTKKNASHGRQSLCSEMGQELRTAMRGGSS

>Cpom-iGluR6
MDRYKLLFILALVLQICQIKAITSARSQFPIWGLFTRLSDDKTVEVFRKFANDSRRSYFGKEKISRSPDSFTVSLE
MCDEGFRSSAIVDGRPTRGICDNVCLRSNKLQIPHLTLDWEPAVSAPKEGFTISYYPPPEVISKVYATYIKKKDW
DRFSFMYEDEGSFIRLQEVINTWENDKKPILFKKIYPEGDNRETFKHVFKVARLSYHIIDCKADNIKKYLEEITKV
VNYTAYQNILLTSLDAYTVDLTSINIEGNVTTWHLTMEQKDKWTDNRIDNPKKIETYLAADALSHLEKSIKYML
DNIPHSSYNIPQPPPLCYDGQTQPWEMGPNLRDALLSTNTAISQGFTGNVEFDDKGKRVNFILHYSKLNNES
QFVYVGNWNSTTDKIEEINLNNVQDVRSNVTFRVVTRKEAPYYMVPKNGDPPKGYVIDLMDEIFKHIREKEH
PGWFHVVDDPGLKVGGPIEGSRRWSGLIGEILEHKAHFAVSDLTITSERNAVVDFSIPFMSLGISMLFKVDPPP
DPDMFSFVNPLSTDVWLYLAVVYIITSLVLLICARMSQEDWVNPHPCDRNPKELQNIWSLYNCMWLTMGSI
MTQGCDILRGAGSRWIAGTWWFFAMIVTASYTANMSTFMSNNRRSNNIENAKDLSEQTAVSYGAMLNGST
HKFFQSSNDPIYQKLYQGMSTANPTAFTANNDEGMERVLRSKGKFVFFMESTTIEYFMQQDCRLKMVGGKL
DSKDYGIAMPKNSPHRMAVNNAILYLQEKGKLQDLKNKWWVKDPDSPDIDECKNEDSSGKKEKEDSGSVQ
MKNTSGIFLVLGVGGILGFIVAIIGFFLHSHEIAVKEGISYKEALASEWRVSLDPRVLSKPAAPPRSAAPSIKSTSPS
RERSRSRAASVLSFAQSFINLNEVY

>Cpom-iGluR7
MIKVLVLGVLAQCVFIQSQFVIGGLFYEGDELDVTLTNSAEHFGFQVLIRKVSRKGELIEAGEHVCNLANEGVIG
IIDGTSGKVTAHVQAICDLLDIPHILIDHNDLIKEDWFHLNLHPSPQAYNMVISKLVELKKWTNVTVMYEKGYS
LMRVSDVLEMASKTMTVSIRELSGKDYRDVLINSKRNGYTNFIVDCPSKKLEQFLKHAQQVGLMADEHSYIFL
SLDLFYKDLTSYRYGGVNMTGFMISQTTDWTMDQSYNEIKQYLIHDAVKLFNETVKMVKDMTLDPGTVNCQ
DYNSWDYGSSILNFMKTNKIEESTGPLIFDEFGQRSDVHINILELTSAGSQPMGDWNFKENDITINRPLLMVP
DITGESIMRNLTFKILVAMVSPFCYLKESATTLTGNDRYEGFAIDLFEKLADELGFQCEFEVTSLKYGGWNETLN

DTDGIAGEIDHGKADFGICDFTITSNRLKAIDFLTPFMSLGIAILYREPSKQPPAMFSFMEVFAPEVWYYMVLIQ
LLLGVIMILVGRLSHKEWQNPQPCIEDPEELSNQFSFANSVWLIIGSVMQQGSEIAPIAFAPRMITSIWWFFT
MIMVASYVGTLVAFLVVEKNVLPFENAYELGESKTIMYGAKDTGSTIQFFKDSQDQKYQDMYKKMVDKHWN
APGNDEGVQKAETMNYAFFMESPSIEYYKQRHCSLMQIGDLLDSKSYGIGIKKGSPYKKVMDDALLKLQENG
ELQKLKDLWWKEKRGGGKCGQETKEEEKQLGMKHMTGVFVVLGVGCILGIVISILDMLWGVFQRSVKYKTT
FKYELVEELKFALKFSGDVKPVKRPAKTDE

>Cpom-iGluR8
MVNLCVTGGLFHPEDDKQEVAFRYAVERVNADRAILPRAKLLAQVETISPQDSFHASKRVCHLLRSGVAAIFG
PQSAPAAAHVQSICDTMELPHLETRWDYRTRRESCLVNLYPHPAALSRAYVDLVRAWGWKSFTIVYENSDGL
VRLQELLKAHGPLELPVAVRQLPDSHDYRPLLKQIKNSAESHIVLDCATDRIRDVLQQAQQIGMMSDYHSYLIT
SLDLHSVDLEEFKYGGTNITSLRLLDPERADVQRVVRDWVYDEARKGRKLQLGHTSAKENMTFIKTETALMYD
AVHLFAKALHDLDTSQQIDVRPLSCEAEDTWPHGYSLINYMKIVEMKGLTGVIKFDHQGFRSDFTLDIIELTRD
GLQKAGIWNSSEGVNYTRSYGENQKQIVEILQNKTLIVTTILSAPYCMRKEASEKLTGNAQFEGYAIDLIHEISKIL
GFNYTFKLAPDGRYGSFNRETKEWDGMIRELLEQRADVAIADLTITYDREQVVDFTMPFMNLGISVLYRKPIK
QPPNLFSFLSPLSLDVWIYMATAYLGVSVLLFILARFSPYEWDSPRNCLDEPQVLENQFTLLNSLWFTIGSLMQ
QGSDIAPKAVSTRMVAGMWWFFTLIMISSYTANLAAFLTVERMDSPIESAEDLAKQTKIKYGALKGGSTAAFF
RDSNFSTYQRMWSFMESARPSVFATSNKEGEERVMRGKGAYAYLMESTTIEYVVERNCDLTQVGGMLDSKG
YGIAMPPNSPYRTAISGAVLKLQEEGKLHILKTKWWKEKRGGGSCRDETSKSSSTANELGLANVGGVFVVLM
GGMGVACVIAVCEFVWKSRKVAVDERKEEASLC

>Cpom-iGluR9
SPSHLHLSYTIANEILPMEIRLEITARETLNALRATLLHTHWHTFTVLAEEDIYSTLKLRKDLSSILDSQPLDPNWL
FLPSKFSQHAIFRRLAKISRLTRGVVVLICDIHYAKLVMDEAKRFNMLDGHFFWLWIDASREIDVFRNIGNRTQ
YSDADNLDFDSVIDKEEALRSEEFERSKRGEVDNNLLKNINDHVPAKYLDEVLNGSSNDENRLLVNNSSRNSFF
HYLSRNKNKSKAHSNNKKILNISFSQSYESRRNISMESVKNNNVNSKGVETSKIENNSIEKDKLSFHVRNNGRR
NSYSKMKNESFNKYVNSINKNNVYNEMKTLLQRDINTEEDVHLRHRLALTSDTSDFLMNPTVQTSTYKLRESI
EKRGEKLNKEYDIDDRSEAMKDNITTIIDSLPIGLLALHPQPMKIDRSFIRAAVRMAVGALRXXXXXXXXXXXXX
XXLSDAAASCWEEPSDAAADFSADFVRETRITSAAALAGGTERPEPALTSSFSFFNLVPGPTGGNVWRQVGHI
HGRMVRLHTIVWPGGRLVAHGQSSGARTIYRIVTALAPPFVMEGELDEDGQCLRGLPCHRPQTSDKDNLTLA
FNDLERDDDDHQPTDFFFPTPKPTILPKMATHCCYGLAMDLLENIAQELEFDFHLYLVEDGLYGSRKLVRSFSKL
HEFTNFLNDEPMFTMSEHLNYRAQFRNGFKANSKEQYSDPNYDDNIDEESQKWNGIVGDLVSGSAHMSFA
ALSVSAARAEVIDFSQPYFYSGISLLAAPNQKADIPLLAFLLPFSTELWIAIFLSLNVTAIAVAIYEWLSPFGLNPW
GRQRSKNFSLSSALWVMWGLLCGHLVAFKAPKSWPNKFLINVWGGFSVIFVASYTANIAALIAGLFFHNAVD
DYQGRNNWLSLRVGTAKSSISEYYVQRGNPQLAQRMRGYALQNIEEGIQRLRNRTLDLLIADTPVLDYYRATD
HGCKLQRVGDHAFIEDTYAIGMAKGFPLQKSISAAIAKYSTNGYMDILTDKWYGGLPCFKLSQDYGIQPKPLG
VAAVAGVFLLLLVGMIVGFLILILEHLFYKYTLPVLRHQPKDAVWRSRNVMFFSQKLYRFINCVELVSPHHAARE
LVNTIRQGHFTSLFQKSVKRKEHEQRRRRRSKAQFFEMIQEIRRVQQGRDQSLDSIKEHVAVETSEVSEELTES
KFLSPSPEVPSRSPRPGRSPRQLRSPRGRRKRCSLAGLNVRRFSTDSVLGSDSGSIYERTSLNIGRRLSRDVSCLT
SSPPDINTRLRTPSPMVRRAEGSSTRSYQDVSEKSERYLSSDGPRSRASVEILVSEEQDVPPAPPYPRVSPTGGR
SELSQLSEEELIRLWRSSEREVREALLAALQERRANLDPKQDPG

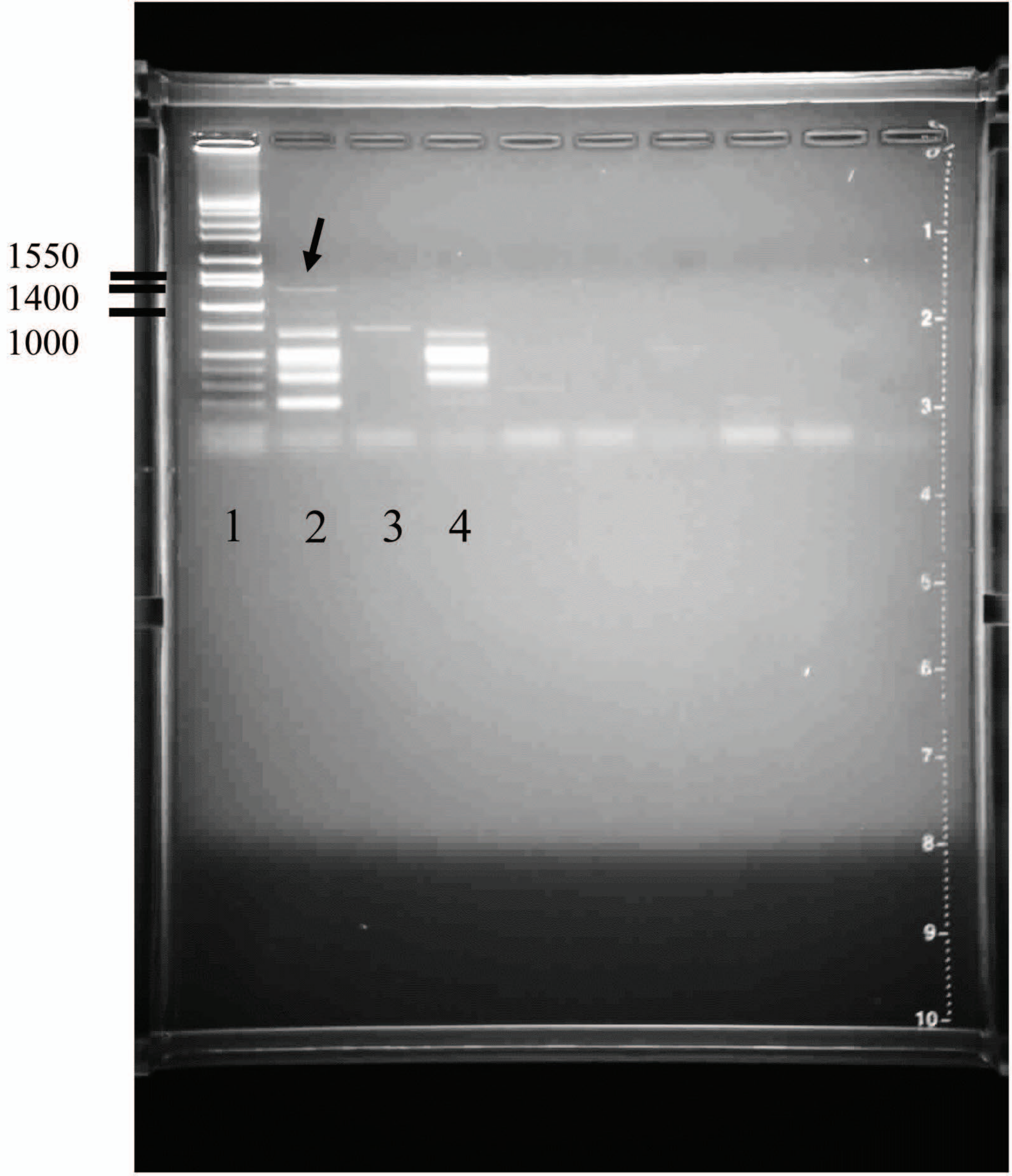

Supplementary Figure S19. Raw gel image showing neonate larval head expression of CpomOR3. Representative PCR amplification of full length ORF of CpomOR3. DNA Molecular Weight ladder is shown in Lane 1, with 1000, 1400 and 1500 bp fragments highlighted. Lane 2 contains PCR product from neonate larval head cDNA with forward and reverse primers. Lane 3 contains PCR product from neonate larval head cDNA with forward primer only. Lane 4 contains PCR product from neonate larval head cDNA with reverse primer only. The arrow points to the full length ORF of CpomOR3; this PCR product has been cloned, sequenced and confirmed as CpomOR3.
